# A generalized interval-valued p,q Rung orthopair fuzzy Maclaurin symmetric mean and modified regret theory based sustainable supplier selection method

**DOI:** 10.1038/s41598-024-64765-3

**Published:** 2024-06-28

**Authors:** Shuang Chen, Jian Ren, KeTing Ye, FeiYan Li

**Affiliations:** 1 School of Advanced Interdisciplinary Studies, Hunan University of Technology and Business, No.569, Yuelu Avenue, Changsha, 410205 China; 2 Xiangjiang Laboratory, Hunan University of Technology and Business, No.569, Yuelu Avenue, Changsha, 410205 China; 3 Research Center for Smart Management of Resource and Environment, No.569, Yuelu Avenue, Changsha, 410205 China; 4 Social Laboratory for Artificial Intelligence in Changsha, No.569, Yuelu Avenue, Changsha, 410205 China

**Keywords:** Interval-valued p,q Rung orthopair fuzzy sets, Generalized Maclaurin symmetric mean, Sustainable supplier selection, Regret theory, Multiple attribute group decision making, Engineering, Mathematics and computing

## Abstract

A novel interval valued p,q Rung orthopair fuzzy (IVPQ-ROF) multiple attribute group decision making (MAGDM) method for sustainable supplier selection (SSS) is proposed in this paper. This study mainly contains two research points: (1) tackling the interrelation between attributes; and (2) describing the psychological state and risk attitude of decision makers (DMs). For the first research point, we introduce the Archimedean operation rules for interval valued p,q Rung orthopair fuzzy sets (IVPQ-ROFSs), then the generalized interval valued p, q Rung orthopair fuzzy Maclaurin symmetric mean (GIVPQ-ROFMSM) operator and the generalized interval valued p, q Rung orthopair fuzzy weighted Maclaurin symmetric mean (GIVPQ-ROFWMSM) operator are defined to reflect the correlation between attributes. For the second research point, we introduce the positive ideal degree (PID) and negative ideal degree (NID) based on projection of IVPQ-ROFSs, and modified regret theory. Both of them consider the best alternative and worst alternative, so as to reflect the psychological state and risk attitude of DMs. Finally, a SSS problem is presented to manifest the effectiveness of the designed method. We also provide sensitivity analysis and comparative analysis to further demonstrate the rationality and validity of the proposed method.

## Introduction

With the awareness of the lack of resources and the emphasis on environmental protection, people pay more and more attention to sustainable supplier chain management (SSCM)^1^. As an important tool for enterprises to obtain core competitiveness and maintain sustainable development, SSCM provides a new method for enterprises to construct sustainable supplier chain and many forward-looking companies are already building their own sustainable supplier chain (SSC)^2^. The establishment and development of SSC requires the full cooperation of upstream and downstream enterprises in the whole chain. Selecting excellent and appropriate suppliers, which can not only reduce the damage to the environment from the source, but also improve the overall sustainable development performance of the enterprise supply chain, so as to reduce costs and increase efficiency for enterprises, has attracted extensive interest from experts and scholars^3–5^.

In the Sustainable supplier selection (SSS) process, the decision makers (DMs) usually evaluate the supplier based on a set of related evaluation attributes, such as: cost, location, credibility and so on^6^. Therefore, SSS problem can be viewed as a multi-attribute group decision making (MAGDM) problem^7^. In the study of MAGDM methods for SSS problems, there are two main research points. The first is the representation of evaluation information, and the second is the design of MAGDM models. Further, DM’s psychological state and risk attitude are also important elements in this process as the DM’s behavior will be influenced by them. Hence, the main research objective of this article is to select the appropriate information form to construct a decision model which both considers the correlation between attributes, the psychological state and the risk attitude of DMs.

For representation of evaluation information, real numerical number is employed to represented the evaluation value in early stage. However, with the increasing complexity and uncertainty in real SSS process, most of the assessment detailed information is unknown, many factors are influenced by uncertainty, and human thinking is always ambiguous^8^. To overcome the uncertainty and ambiguity in SSS process, fuzzy sets (FSs) theory^9^ is widely used in SSS problem, such as Intuitionistic fuzzy sets (IFSs)^10^, Pythagorean fuzzy sets (PFSs)^11^, Fermatean fuzzy sets (FFSs)^12^ and Q-Rung orthopair fuzzy sets (Q-ROFSs)^13^. Chai et al.^14^ introduced intuitionistic fuzzy SSS approach based on cumulative prospect theory. Rouyendegh^15^ designed an intuitionistic fuzzy TOPSIS ethmod for green supplier selection problem. Giri et al.^16^ proposed a pythagorean fuzzy DEMATEL method for supplier selection in SSCM. Wei et al.^17^ utilized the FFSs to represent the evaluation information in green supplier selection. Güneri and Deveci^18^ evaluated the sustainable supplier based on Q-Rung orthopair fuzzy EDAS method. The above studies are all successful applications of FSs theory in SSS problems, making great contributions to the research of FSs theory and SCM.

Even though the above fuzzy sets provide effective forms for the evaluation information in SSS problems, there is still a special case that cannot be solved by them. It is a normal phenomenon that there is a shortage in available information in real decision problems, and evaluation information given by DMs is usually in interval valued form rather than accurate numbers^19,20^. Therefore, many scholars have extended the above fuzzy sets, with Atanassov^21^ proposing interval-valued intuitionistic fuzzy sets (IVIFSs) and Peng^22^ introducing the interval-valued pythagorean fuzzy sets (IVPFSs). Further, FFSs and Q-ROFSs are also extended into interval-valued form^19,23^. Numerous studies have shown that compared to non interval-valued fuzzy sets (IVFSs), IVFSs can provide greater decision-making flexibility and better describe the uncertainty in the SSS process. For example, Perçin^24^ employed IVIFSs to select circular supplier. Afzali et al.^25^ introduced an interval-valued intuitionistic fuzzy-based CODAS for sustainable supplier selection. Yu et al.^26^ designed an interval-valued pythagorean fuzzy TOPSIS method to solve SSS problem. Wang et al.^27^ introduced a MAGDM method with q-rung interval-valued orthopair fuzzy information.

Nonetheless, Ali et al.^28^ argued that the IVQ-ROFSs is unable to fulfill a special requirement due to the fact that, in IVQ-ROFSs, the term levels of the upper bounds of membership degree (MD) and non-membership degree (NMD) are considered identical, i.e., $0 \le \left( {\mu^{U} } \right)^{q} + \left( {\nu^{U} } \right)^{q} \le 1$. For instance, if we consider $\mu^{U} = 0.8$ and $\nu^{U} = 0.7$, then clearly $0.8^{2} + 0.7^{2} = 1.13 > 1$. Therefore, we next check $0.8^{3} + 0.7^{3} = 0.855 < 1$. However, $0.8^{4} + 0.7^{2} = 0.8996 < 1$. Hence, to more effectively satisfy this requirement, Ali et al.^28^ introduced interval-valued p,q Rung orthopair fuzzy sets (IVPQ-ROFSs), which could degenerates into IVIFSs when $p = q = 1$, IVPFSs when $p = q = 2$, IVFFSs when $p = q = 3$, IVQ-ROFSs when $p = q$. Obviously, IVPQ-ROFS is a more generalized form that is worth using for SSS problems.

Even though IVPQ-ROFS introduced by Ali et al.^28^ is a more generalized form of IVFS, there are still some shortcomings in the study of Ali et al.^28^. First, the operational laws of IVPQ-ROFS in literature^28^ are defined based on Algebraic T-norm (TN) and T-conorm, which are special cases of Archimedean T-norm (ATN) and Archimedean T-conorm (ATCN). Second, the interval-valued p,q Rung orthopair fuzzy weighted average (IVPQ-ROFWA) operator^28^ cannot handle the correlation between attributes. Finally, the distance measure^28^ of interval-valued p,q Rung orthopair fuzzy numbers (IVPQ-ROFNs) only considers the distance between IVPQ-ROFNs, but ignores the angle between them. Hence, the theoretical basis of IVPQ-ROFSs needs to be further improved.

For the design of MAGDM models, there are often two types of methods to solve MAGDM problems. The one type is traditional methods, such as TOPSIS method^29,30^, VIKOR method^31^, TODIM method^32^ and so on^33,34^. The other one type is aggregation operators (AOs). The other one type is aggregation operators (AOs). Compared with traditional methods, AO could not only give the total ranking order of all alternatives, but also provide the comprehensive score values of them^35^. Therefore, many scholars have researched the application of AOs in MAGDM problems. The main research emphasizes of AO are usually composed of two aspects: the operational laws and the functions^35^. For the operational laws, various AOs are from the Algebraic operational laws which are critical members in the family of T-norm (TN) and T-conorm (TCN). It is worth noting that the Archimedean T-norm (ATN) and Archimedean T-conorm (ATCN) are the generalization of many TNs and TCNs. Various ATN and ATCN can be used to introduce the operational rules, such as: Einstein TN and TCN^36^, Hamacher TN and TCN^37,38^, Frank TN and TCN^39^, Dombi TN and TCN^40^. Aczel–Alsina TN and TCN^41–43^ are also very popular research point in MAGDM method. For the functions, the existing AOs can be classified into two categories: (1) The one type AOs^44,45^ only aggregate the input arguments into one. (2) The other one type AOs^46–50^ not only aggregate the input arguments but also consider the interrelationships between them. Obviously, the latter is more suitable than the former to deal with the MAGDM problem with correlation among attributes.

To solve MAGDM problems with interacted attributes, a lot of information AOs have been proposed like Bonferroni mean (BM)^51^ operator, Maclaurin symmetric mean (MSM)^52^ operator, Hamy mean (HM)^53^ operator, generalized Maclaurin symmetric mean (GMSM)^54^ operator. Further, the GMSM operator is a generalization of BM operator, MSM operator and HM operator since the GMSM operator can be transformed into different forms when setting corresponding parameter values^35^. Hence, it is meaningful and necessary to apply the GMSM operator to solve MAGDM problems with correlated attributes under IVPQ-ROF environment.

As we have stated before, the risk attitude and psychological state of DMs are also very important factors in the SSS process. In order to effectively describe the risk attitude of DMs, the prospect theory based-method^55^ and TODIM method^56^ are put forward to solve MAGDM problem. However, Wang^57^ indicated that DMs will not only feel the risk, but feel regret, when he knows the best plan is missed. As a representative behavioral theory, regret theory^58^ can effectively deal with this problem. Wang^57^ proposed a projection-based regret theory method under IT2F environment. Pan^59^ introduced a new regret theory-based risk decision-making method for renewable energy investment problem. Wang et al.^60^ construct a regret-based three-way decision model under interval type-2 fuzzy (IT2F) environment. Nonetheless, all of the above methods based on regret theory only compare with the best alternative, but ignore the worst alternative when calculating the regret-rejoice value, which may lead to undesirable consequences.

Based on the above analysis, the main research motivations of this paper are organized as follows:

1. Design a novel MAGDM method for SSS problems, which utilizes a more generalized form of FSs-IVPQ-ROFSs. This method can handle the correlation between attributes, describe decision-makers' risk preferences and psychological states.
2. Improve the relevant theories of IVPQ-ROFSs, such as operation rules and differences measures. Although Ali et al.^28^ defines the operation rules and distance formulas for IVPQ-ROFSs, there are still some shortcomings, such as the operation rules are designed based on Algebraic TN and TCN, which are the special case of ATN and ATCN. Further, the distance measure^28^ does not consider the angle between two interval-valued p,q Rung orthopair fuzzy numbers (IVPQ-ROFNs).
3. Extend the GMSM operator to IVPQ-ROF environments, and derive different forms of GMSM operators based on ATN and ATCN.
4. Modify the traditional regret theory^57–60^ which compare with the best alternative, but ignore the worst alternative when calculating the regret-rejoice value.

The structure of this paper is organized as follows. We briefly review several preliminary definitions and concepts of IVPQ-ROFS, ATN and ATCN, GMSM operator and regret theory in “Preliminaries” section. In “The operational laws of IVPQ-ROFN based on ATN and ATCN” section, we define the general operational rules of IVPQ-ROFNs based on ATN and ATCN, and study some particular cases when taking different generator. In “Some GIVPQ-ROFMSM operators based on ATN and ATCN” section, we introduce the GIVPQ-QOFMSM operator and GIVPQ-QOFWMSM operator, and study some particular cases and valuable properties of them. In “The PID and NID based on the projection measure of IVPQ-ROFNs” section, we define the positive ideal degree and negative ideal degree based on the projection measure of IVPQ-ROFNs. In “The created approaches to IVPQ-ROF MAGDM problems with regret theory” section, we propose a novel IVPQ-ROF MAGDM method with modified regret theory. In “Case study” section, an example of SSS is presented to demonstrate the application of the proposed method. Also, some sensitivity analyses are conducted. Finally, comparative analysis and concluding remarks are presented in “Comparative study” section and “Conclusions and future work” section, respectively.

## Preliminaries

In this section, we will introduce some basic concepts related to this paper, such as IVPQ-QOFS, ATN, ATCN, GMSM operator and regret theory.

### Interval-valued p,q Rung orthopair fuzzy set

#### Definition 2.1

^28^ Let $X$ be a nonempty fixed set, an IVPQ‐ROFS $\mathop Q\limits^{\sim }$ on $X$ can be expressed as follows:

$$ \mathop Q\limits^{\sim } = \left\{ {\left\langle {x,\left( {\left[ {\mu_{{\mathop Q\limits^{\sim } }}^{L} \left( x \right),\mu_{{\mathop Q\limits^{\sim } }}^{U} \left( x \right)} \right],\left[ {\nu_{{\mathop Q\limits^{\sim } }}^{L} \left( x \right),\nu_{{\mathop Q\limits^{\sim } }}^{U} \left( x \right)} \right]} \right)} \right\rangle |x \in X} \right\} $$

where $\left[ {\mu_{{\mathop Q\limits^{\sim } }}^{L} \left( x \right),\mu_{{\mathop Q\limits^{\sim } }}^{U} \left( x \right)} \right] \subseteq \left[ {0,1} \right]$ and $\left[ {\nu_{{\mathop Q\limits^{\sim } }}^{L} \left( x \right),\nu_{{\mathop Q\limits^{\sim } }}^{U} \left( x \right)} \right] \subseteq \left[ {0,1} \right]$ denote the membership degree and non-membership degree of $x \in X$ to $\mathop Q\limits^{\sim }$, respectively. Given some accurate numbers $p$ and $q$, then $\left( {\mu_{{\mathop Q\limits^{\sim } }}^{U} \left( x \right)} \right)^{p} + \left( {\nu_{{\mathop Q\limits^{\sim } }}^{U} \left( x \right)} \right)^{q} \le 1$ for all $x \in X$. The hesitancy degree is denoted by $\left[ {\pi_{Q}^{L} \left( x \right),\pi_{Q}^{U} \left( x \right)} \right] = \left[ {\sqrt[r]{{1 - \left( {\mu_{Q}^{U} \left( x \right)} \right)^{p} - \left( {\nu_{Q}^{U} \left( x \right)} \right)^{q} }},\sqrt[r]{{1 - \left( {\mu_{Q}^{L} \left( x \right)} \right)^{p} - \left( {\nu_{Q}^{L} \left( x \right)} \right)^{q} }}} \right]$, where $r$ is the least common multiple of $p$ and $q$. For convenience, we call $Q = \left( {\left[ {\mu^{L} ,\mu^{U} } \right],\left[ {\nu^{L} ,\nu^{U} } \right]} \right)$ an IVPQ-ROFN.

#### Definition 2.2

^28^ Let $Q_{i} = \left( {\left[ {\mu_{i}^{L} ,\mu_{i}^{U} } \right],\left[ {\nu_{i}^{L} ,\nu_{i}^{U} } \right]} \right)\left( {i = 1,2} \right)$ be any two IVPQ-ROFNs, $p,q \ge 1$ and $\eta > 0$, the operational laws for the IVPQ-ROFNs based on Archimedean TN and TCN can be defined as follows:

1. $$Q_{1} \oplus Q_{2} = \left( {\left[ \begin{gathered} \left( {\left( {\mu_{1}^{L} } \right)^{p} + \left( {\mu_{2}^{L} } \right)^{p} - \left( {\mu_{1}^{L} } \right)^{p} \left( {\mu_{2}^{L} } \right)^{p} } \right)^{\frac{1}{p}} , \hfill \\ \left( {\left( {\mu_{1}^{U} } \right)^{p} + \left( {\mu_{2}^{U} } \right)^{p} - \left( {\mu_{1}^{U} } \right)^{p} \left( {\mu_{2}^{U} } \right)^{p} } \right)^{\frac{1}{p}} \hfill \\ \end{gathered} \right],\left[ {\nu_{1}^{L} \nu_{2}^{L} ,\nu_{1}^{U} \nu_{2}^{U} } \right]} \right)$$
2. $$Q_{1} \otimes Q_{2} = \left( {\left[ {\mu_{1}^{L} \mu_{2}^{L} ,\mu_{1}^{U} \mu_{2}^{U} } \right],\left[ \begin{gathered} \left( {\left( {\nu_{1}^{L} } \right)^{q} + \left( {\nu_{2}^{L} } \right)^{q} - \left( {\nu_{1}^{L} } \right)^{q} \left( {\nu_{2}^{L} } \right)^{q} } \right)^{\frac{1}{q}} , \hfill \\ \left( {\left( {\nu_{1}^{U} } \right)^{q} + \left( {\nu_{2}^{U} } \right)^{q} - \left( {\nu_{1}^{U} } \right)^{q} \left( {\nu_{2}^{U} } \right)^{q} } \right)^{\frac{1}{q}} \hfill \\ \end{gathered} \right]} \right)$$
3. $$\eta Q_{1} = \left( {\left[ {\left( {1 - \left( {1 - \left( {\mu_{1}^{L} } \right)^{p} } \right)^{\eta } } \right)^{\frac{1}{p}} ,\left( {1 - \left( {1 - \left( {\mu_{1}^{U} } \right)^{p} } \right)^{\eta } } \right)^{\frac{1}{p}} } \right],\left[ {\left( {\nu_{1}^{L} } \right)^{\eta } ,\left( {\nu_{1}^{U} } \right)^{\eta } } \right]} \right)$$
4. $$\left( {Q_{1} } \right)^{\eta } = \left( {\left[ {\left( {\mu_{1}^{L} } \right)^{\eta } ,\left( {\mu_{1}^{U} } \right)^{\eta } } \right],\left[ {\left( {1 - \left( {1 - \left( {\nu_{1}^{L} } \right)^{q} } \right)^{\eta } } \right)^{\frac{1}{q}} ,\left( {1 - \left( {1 - \left( {\nu_{1}^{U} } \right)^{q} } \right)^{\eta } } \right)^{\frac{1}{q}} } \right]} \right)$$

#### Definition 2.3

^28^ Let $Q_{i} = \left( {\left[ {\mu_{i}^{L} ,\mu_{i}^{U} } \right],\left[ {\nu_{i}^{L} ,\nu_{i}^{U} } \right]} \right)\left( {i = 1,2,...,n} \right)$ be $n$ IVPQ-ROFNs, and $w = \left( {w_{1} ,w_{2} ,...,w_{n} } \right)$ be the weight vector with $\sum\limits_{i = 1}^{n} {w_{i} } = 1$. Then the IVPQ-ROFWA operator is defined as follows:

1 $$ \begin{gathered} Q = IVPQ - ROFWA\left( {Q_{1} ,Q_{2} ,...,Q_{n} } \right) \\ = \left( {\left[ {\left( {1 - \prod\limits_{i = 1}^{n} {\left( {1 - \left( {\mu_{i}^{L} } \right)^{p} } \right)^{{w_{i} }} } } \right)^{\frac{1}{p}} ,\left( {1 - \prod\limits_{i = 1}^{n} {\left( {1 - \left( {\mu_{i}^{U} } \right)^{p} } \right)^{{w_{i} }} } } \right)^{\frac{1}{p}} } \right],\left[ {\prod\limits_{i = 1}^{n} {\left( {\nu_{i}^{L} } \right)^{{w_{i} }} } ,\prod\limits_{i = 1}^{n} {\left( {\nu_{i}^{U} } \right)^{{w_{i} }} } } \right]} \right) \\ \end{gathered} $$

### Archimedean T-norm and T-conorm

The triangle operators are the intersection and union operators which are expressed by TN and TCN, respectively, and they have received a lot of attentions^61–63^. For the TN and TCN, we have the following:

1. If a monotonic decreasing function $g$ satisfies the following conditions:
  1. $$g\left( t \right):\left( {0,1} \right] \to \left[ {0,\infty } \right]$$ and $$g^{ - 1} \left( t \right):\left[ {0,\infty } \right] \to \left( {0,1} \right]$$;
  2. $$\mathop {\lim }\limits_{t \to \infty } g^{ - 1} \left( t \right) = 0$$;
  3. $$g^{ - 1} \left( 0 \right) = 1$$.
    Then, the function $g$ can be utilized to generate TN $T\left( {x,y} \right) = g^{ - 1} \left( {g\left( x \right) + g\left( y \right)} \right)$.
2. If a monotonic increasing function $h$ satisfies the following conditions:
  1. $$h\left( t \right):\left( {0,1} \right] \to \left[ {0,\infty } \right]$$ and $$h^{ - 1} \left( t \right):\left[ {0,\infty } \right] \to \left( {0,1} \right]$$;
  2. $$\mathop {\lim }\limits_{t \to \infty } h^{ - 1} \left( t \right) = 1$$;
  3. $$h^{ - 1} \left( 0 \right) = 0$$.
  Then, the function $h$ can be utilized to generate TCN $S\left( {x,y} \right) = h^{ - 1} \left( {h\left( x \right) + h\left( y \right)} \right)$.

We present some famous families of Archimedean T-norm (ATN), Archimedean T-conorm (ATCN) and their additive generators in Tables 1, 2, respectively.

**Table 1:** T-norms and corresponding additive generators.

Name	T-norms	Additive generators
Algebraic	$T_{A} \left( {x,y} \right) = xy$	$g\left( t \right) = - \log \left( t \right)$
Einstein	$T_{E} \left( {x,y} \right) = \frac{xy}{{1 + \left( {1 - x} \right)\left( {1 - y} \right)}}$	$g\left( t \right) = - \log \left( {\frac{2 - t}{t}} \right)$
Hamacher	$T_{H,\gamma } \left( {x,y} \right) = \frac{xy}{{\gamma + \left( {1 - \gamma } \right)\left( {x + y - xy} \right)}}$	$g\left( t \right) = - \log \left( {\frac{{\gamma + \left( {1 - \gamma } \right)t}}{t}} \right),\gamma > 0$
Frank	$T_{F,\tau } \left( {x,y} \right) = \log_{\tau } \left( {1 + \frac{{\left( {\tau^{x} - 1} \right)\left( {\tau^{y} - 1} \right)}}{\tau - 1}} \right)$	$$g\left( t \right) = \left\{ \begin{gathered} - \log \left( t \right),\tau = 1 \hfill \\ - \log \left( {\frac{\tau - 1}{{\tau^{t} - 1}}} \right),\tau \ne 1 \hfill \\ \end{gathered} \right.$$

**Table 2:** T-conorms and corresponding additive generators.

Name	T-conorms	Additive generators
Algebraic	$S_{A} \left( {x,y} \right) = x + y - xy$	$h\left( t \right) = - \log \left( {1 - t} \right)$
Einstein	$S_{E} \left( {x,y} \right) = \frac{x + y}{{1 + xy}}$	$h\left( t \right) = - \log \left( {\frac{1 + t}{{1 - t}}} \right)$
Hamacher	$S_{H,\gamma } \left( {x,y} \right) = \frac{{x + y - xy - \left( {1 - \gamma } \right)xy}}{{1 - \left( {1 - \gamma } \right)xy}}$	$h\left( t \right) = - \log \left( {\frac{{\gamma + \left( {1 - \gamma } \right)\left( {1 - t} \right)}}{1 - t}} \right),\gamma > 0$
Frank	$S_{F,\tau } \left( {x,y} \right) = \log_{\tau } \left( {1 + \frac{{\left( {\tau^{x} - 1} \right)\left( {\tau^{y} - 1} \right)}}{\tau - 1}} \right)$	$$h\left( t \right) = \left\{ \begin{gathered} - \log \left( {1 - t} \right),\tau = 1 \hfill \\ - \log \left( {\frac{\tau - 1}{{\tau^{1 - t} - 1}}} \right),\tau \ne 1 \hfill \\ \end{gathered} \right.$$

### Generalized Maclaurin symmetric mean operator

#### Definition 2.4

^54^ Let $x_{i} \left( {i = 1,2,...,n} \right)$ be a set of non-negative numbers and $k = 1,2,...,n$. Then, the GMSM operator is defined as follows:

2 $$ GMSM^{{\left( {k,\lambda_{1} ,\lambda_{2} ,...,\lambda_{k} } \right)}} \left( {x_{1} ,x_{2} ,...,x_{n} } \right) = \left( {\frac{1}{{C_{n}^{k} }}\sum\limits_{{1 \le i_{1} < ... < i_{k} \le n}} {\left( {\prod\limits_{j = 1}^{k} {x_{{i_{j} }}^{{\lambda_{j} }} } } \right)} } \right)^{{\frac{1}{{\lambda_{1} + \lambda_{2} + ... + \lambda_{k} }}}} $$

where $\left( {i_{1} ,i_{2} ,...,i_{k} } \right)$ traverses all k-tuple combination of $\left( {1,2,...,n} \right)$, $C_{n}^{k} = \frac{n!}{{k!\left( {n - k} \right)!}}$ is the binomial coefficient.

### Regret theory

Regret theory, proposed by Bell^53^ in 1982, is a behavioral decision theory which holds that people tend to care not only about what they can get, but also compare the results of the scheme to be selected with those of other alternatives. If decision makers find that they can get better results by choosing other schemes, they may feel regret in their hearts; On the contrary, they will feel happy.

#### Definition 2.5

**Figure 1:**
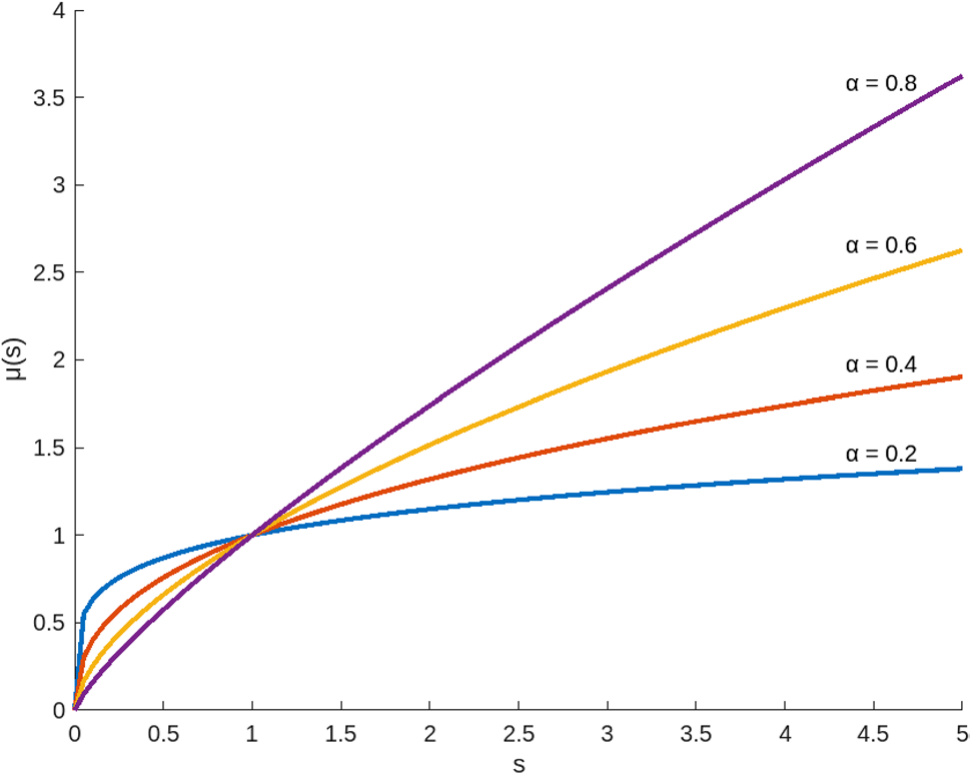
The utility function $u\left( s \right)$.

^57^ Let $s$ be the attribute value, then the utility function $u\left( s \right)$ which shown in Fig. 1 can be defined as follows:

3 $$ u\left( s \right) = s^{\alpha } ,0 < \alpha < 1 $$

where $\alpha$ denotes the risk aversion coefficient of decision maker, the smaller the value of $\alpha$, the lager the risk aversion value is. On the contrary, a lager value of $\alpha$ indicates that the decision maker prefers risk.

#### Definition 2.6

**Figure 2:**
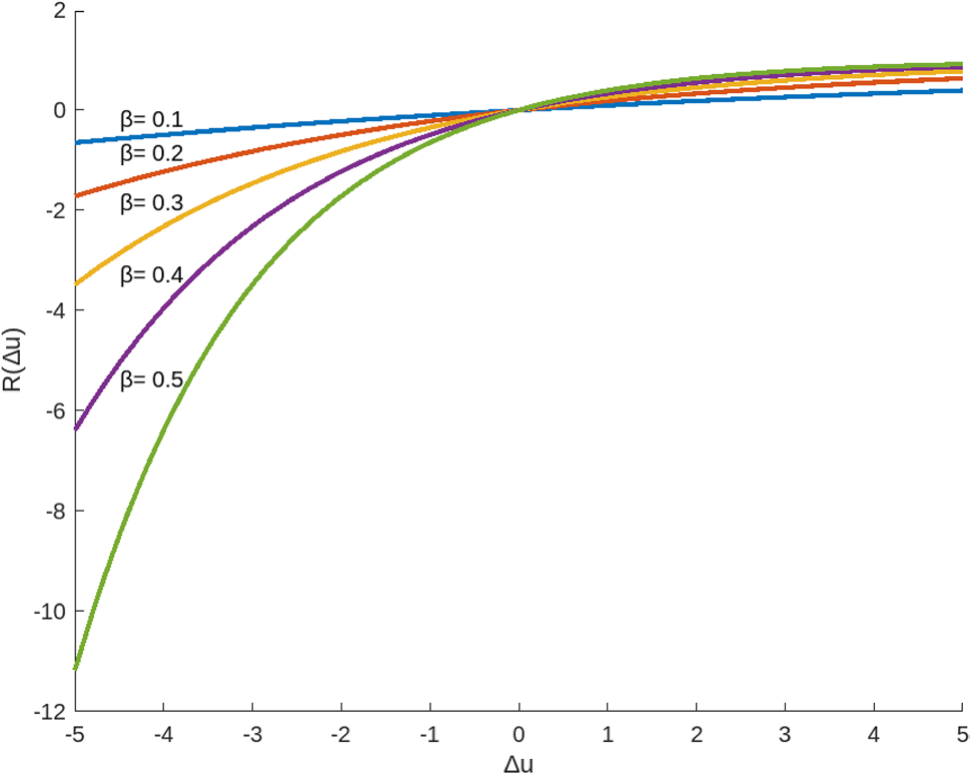
The regret-rejoice function $R\left( {\Delta u} \right)$.

^57^ Let $s_{1}$ and $s_{1}$ denote the evaluation value of object $o_{1}$ and $o_{2}$ respectively. The regret-rejoice function $R\left( {\Delta u} \right)$ which shown in Fig. 2 can be defined as follows:

4 $$ R\left( {\Delta u} \right) = 1 - \exp \left\{ { - \beta \left( {\Delta u} \right)} \right\},\Delta u = u\left( {s_{1} } \right) - u\left( {s_{2} } \right) $$

where $\beta$ denotes the regret aversion coefficient of decision maker and a larger $\beta$ represents a lager risk aversion value. The difference between utility values of $o_{1}$ and $o_{2}$ is denoted by $\Delta u$.

The regret theory was initially used to tackle binomial alternative problems, but the MAGDM problems in reality usually consist of multiple alternatives. Hence, some scholars^57–60^ modified and extended the regret theory to apply it to MAGDM problems. Liang^64^ further defined the regret value and rejoice value of alternative $o_{i}$ as follows:

1. 5 $$ {\text{Regret value}}{:}\;\;REG_{i} = 1 - \exp \left\{ { - \alpha \left( {u\left( {o_{i} } \right) - \mathop {\max }\limits_{i} \left( {u\left( {o_{i} } \right)} \right)} \right)} \right\} $$
2. 6 $$ {\text{Rejoice value}}{:}\;\;REJ_{i} = 1 - \exp \left\{ { - \alpha \left( {u\left( {o_{i} } \right) - \mathop {\min }\limits_{i} \left( {u\left( {o_{i} } \right)} \right)} \right)} \right\} $$

Then the regret-rejoice value of alternative $o_{i}$ is presented as follows:

7 $$ {\mathbb{R}}\left( {o_{i} } \right) = REG_{i} + REJ_{i} $$

## The operational laws of IVPQ-ROFN based on ATN and ATCN

In this section, we define the operational laws of IVPQ-ROFN based on ATN and ATCN. Then, some special cases about the operational laws of IVPQ-QOFN when taking different additive generators in Tables 1, 2 are introduced. We also give some computational examples under different operational laws of IVPQ-QOFN.

### Definition 3.1

$Q_{i} = \left( {\left[ {\mu_{i}^{L} ,\mu_{i}^{U} } \right],\left[ {\nu_{i}^{L} ,\nu_{i}^{U} } \right]} \right)\left( {i = 1,2} \right)$ be any two IVPQ-ROFNs, $p,q \ge 1$ and $\eta > 0$, the operational laws for the IVPQ-ROFNs based on Archimedean TN and TCN can be defined as follows:

1. $$Q_{1} \oplus Q_{2} = \left( \begin{gathered} \left[ {h^{ - 1} \left( {h\left( {\mu_{1}^{L} } \right) + h\left( {\mu_{2}^{L} } \right)} \right),h^{ - 1} \left( {h\left( {\mu_{1}^{U} } \right) + h\left( {\mu_{2}^{U} } \right)} \right)} \right], \hfill \\ \left[ {g^{ - 1} \left( {g\left( {\nu_{1}^{L} } \right) + g\left( {\nu_{2}^{L} } \right)} \right),g^{ - 1} \left( {g\left( {\nu_{1}^{U} } \right) + g\left( {\nu_{2}^{U} } \right)} \right)} \right] \hfill \\ \end{gathered} \right)$$
2. $$Q_{1} \otimes Q_{2} = \left( \begin{gathered} \left[ {g^{ - 1} \left( {g\left( {\mu_{1}^{L} } \right) + g\left( {\mu_{2}^{L} } \right)} \right),g^{ - 1} \left( {g\left( {\mu_{1}^{U} } \right) + g\left( {\mu_{2}^{U} } \right)} \right)} \right], \hfill \\ \left[ {h^{ - 1} \left( {h\left( {\nu_{1}^{L} } \right) + h\left( {\nu_{2}^{L} } \right)} \right),h^{ - 1} \left( {h\left( {\nu_{1}^{U} } \right) + h\left( {\nu_{2}^{U} } \right)} \right)} \right] \hfill \\ \end{gathered} \right)$$
3. $$\eta Q_{1} = \left( {\left[ {h^{ - 1} \left( {\eta h\left( {\mu_{1}^{L} } \right)} \right),h^{ - 1} \left( {\eta h\left( {\mu_{1}^{U} } \right)} \right)} \right],\left[ {g^{ - 1} \left( {\eta g\left( {\nu_{1}^{U} } \right)} \right),g^{ - 1} \left( {\eta g\left( {\nu_{1}^{U} } \right)} \right)} \right]} \right)$$
4. $$\left( {Q_{1} } \right)^{\eta } = \left( {\left[ {g^{ - 1} \left( {\eta g\left( {\mu_{1}^{L} } \right)} \right),g^{ - 1} \left( {\eta g\left( {\mu_{1}^{U} } \right)} \right)} \right],\left[ {h^{ - 1} \left( {\eta h\left( {\nu_{1}^{U} } \right)} \right),h^{ - 1} \left( {\eta h\left( {\nu_{1}^{U} } \right)} \right)} \right]} \right)$$

### Theorem 1

Let $Q_{1}$, $Q_{2}$ and $Q$ are three IVPQ-ROFNs, then

1. $$Q_{1} \oplus Q_{2} = Q_{2} \oplus Q_{1}$$
2. $$Q_{1} \otimes Q_{2} = Q_{2} \otimes Q_{1}$$
3. $$\eta \left( {Q_{1} \oplus Q_{2} } \right) = \eta Q_{1} \oplus \eta Q_{2}$$
4. $$\left( {\eta_{1} + \eta_{2} } \right)Q = \eta_{1} Q + \eta_{2} Q$$
5. $$\left( {Q_{1} \otimes Q_{2} } \right)^{\eta } = Q_{1}^{\eta } \otimes Q_{2}^{\eta }$$
6. $$Q^{{\eta_{1} }} \otimes Q^{{\eta_{2} }} = Q^{{\eta_{1} + \eta_{2} }}$$

### Proof

It can be easily deduced based on Definition 3.1, so omitted here.

Based on different additive generators, we can obtain the following special cases of the operational laws of IVPQ-ROFNs.

1. If $$g\left( x \right) = - \log x^{p}$$ and $$h\left( x \right) = - \log \left( {1 - x^{q} } \right)$$, the operational laws for the IVPQ-ROFNs based on Algebraic TN and TCN can be defined as follows:
  1. $$Q_{1} \oplus Q_{2} = \left( {\left[ \begin{gathered} \left( {\left( {\mu_{1}^{L} } \right)^{p} + \left( {\mu_{2}^{L} } \right)^{p} - \left( {\mu_{1}^{L} } \right)^{p} \left( {\mu_{2}^{L} } \right)^{p} } \right)^{\frac{1}{p}} , \hfill \\ \left( {\left( {\mu_{1}^{U} } \right)^{p} + \left( {\mu_{2}^{U} } \right)^{p} - \left( {\mu_{1}^{U} } \right)^{p} \left( {\mu_{2}^{U} } \right)^{p} } \right)^{\frac{1}{p}} \hfill \\ \end{gathered} \right],\left[ {\nu_{1}^{L} \nu_{2}^{L} ,\nu_{1}^{U} \nu_{2}^{U} } \right]} \right)$$
  2. $$Q_{1} \otimes Q_{2} = \left( {\left[ {\mu_{1}^{L} \mu_{2}^{L} ,\mu_{1}^{U} \mu_{2}^{U} } \right],\left[ \begin{gathered} \left( {\left( {\nu_{1}^{L} } \right)^{q} + \left( {\nu_{2}^{L} } \right)^{q} - \left( {\nu_{1}^{L} } \right)^{q} \left( {\nu_{2}^{L} } \right)^{q} } \right)^{\frac{1}{q}} , \hfill \\ \left( {\left( {\nu_{1}^{U} } \right)^{q} + \left( {\nu_{2}^{U} } \right)^{q} - \left( {\nu_{1}^{U} } \right)^{q} \left( {\nu_{2}^{U} } \right)^{q} } \right)^{\frac{1}{q}} \hfill \\ \end{gathered} \right]} \right)$$
  3. $$\eta Q_{1} = \left( {\left[ {\left( {1 - \left( {1 - \left( {\mu_{1}^{L} } \right)^{p} } \right)^{\eta } } \right)^{\frac{1}{p}} ,\left( {1 - \left( {1 - \left( {\mu_{1}^{U} } \right)^{p} } \right)^{\eta } } \right)^{\frac{1}{p}} } \right],\left[ {\left( {\nu_{1}^{L} } \right)^{\eta } ,\left( {\nu_{1}^{U} } \right)^{\eta } } \right]} \right)$$
  4. $$\left( {Q_{1} } \right)^{\eta } = \left( {\left[ {\left( {\mu_{1}^{L} } \right)^{\eta } ,\left( {\mu_{1}^{U} } \right)^{\eta } } \right],\left[ {\left( {1 - \left( {1 - \left( {\nu_{1}^{L} } \right)^{q} } \right)^{\eta } } \right)^{\frac{1}{q}} ,\left( {1 - \left( {1 - \left( {\nu_{1}^{U} } \right)^{q} } \right)^{\eta } } \right)^{\frac{1}{q}} } \right]} \right)$$
2. If $$g\left( x \right) = \log \left( {\frac{{2 - x^{p} }}{{x^{p} }}} \right)$$ and $$h\left( x \right) = \log \left( {\frac{{1 + x^{q} }}{{1 - x^{q} }}} \right)$$, the operational laws for the IVPQ-ROFNs based on Einstein TN and TCN can be defined as follows:
  1. $$Q_{1} \oplus Q_{2} = \left( {\left[ \begin{gathered} \left( {\frac{{\left( {\mu_{1}^{L} } \right)^{p} + \left( {\mu_{2}^{L} } \right)^{p} }}{{1 + \left( {\mu_{1}^{L} } \right)^{p} \left( {\mu_{2}^{L} } \right)^{p} }}} \right)^{\frac{1}{p}} , \hfill \\ \left( {\frac{{\left( {\mu_{1}^{U} } \right)^{p} + \left( {\mu_{2}^{U} } \right)^{p} }}{{1 + \left( {\mu_{1}^{U} } \right)^{p} \left( {\mu_{2}^{U} } \right)^{p} }}} \right)^{\frac{1}{p}} \hfill \\ \end{gathered} \right],\left[ \begin{gathered} \frac{{\nu_{1}^{L} \nu_{2}^{L} }}{{\left( {1 + \left( {1 - \left( {\nu_{1}^{L} } \right)^{q} } \right)\left( {1 - \left( {\nu_{2}^{L} } \right)^{q} } \right)} \right)^{\frac{1}{q}} }}, \hfill \\ \frac{{\nu_{1}^{U} \nu_{2}^{U} }}{{\left( {1 + \left( {1 - \left( {\nu_{1}^{U} } \right)^{q} } \right)\left( {1 - \left( {\nu_{2}^{U} } \right)^{q} } \right)} \right)^{\frac{1}{q}} }} \hfill \\ \end{gathered} \right]} \right)$$
  2. $$Q_{1} \otimes Q_{2} = \left( {\left[ \begin{gathered} \frac{{\mu_{1}^{L} \mu_{2}^{L} }}{{\left( {1 + \left( {1 - \left( {\mu_{1}^{L} } \right)^{p} } \right)\left( {1 - \left( {\mu_{2}^{L} } \right)^{p} } \right)} \right)^{\frac{1}{p}} }}, \hfill \\ \frac{{\mu_{1}^{U} \mu_{2}^{U} }}{{\left( {1 + \left( {1 - \left( {\mu_{1}^{U} } \right)^{p} } \right)\left( {1 - \left( {\mu_{2}^{U} } \right)^{p} } \right)} \right)^{\frac{1}{p}} }} \hfill \\ \end{gathered} \right],\left[ \begin{gathered} \left( {\frac{{\left( {\nu_{1}^{L} } \right)^{q} + \left( {\nu_{2}^{L} } \right)^{q} }}{{1 + \left( {\nu_{1}^{L} } \right)^{q} \left( {\nu_{2}^{L} } \right)^{q} }}} \right)^{\frac{1}{q}} , \hfill \\ \left( {\frac{{\left( {\nu_{1}^{U} } \right)^{q} + \left( {\nu_{2}^{U} } \right)^{q} }}{{1 + \left( {\nu_{1}^{U} } \right)^{q} \left( {\nu_{2}^{U} } \right)^{q} }}} \right)^{\frac{1}{q}} \hfill \\ \end{gathered} \right]} \right)$$
  3. $$\eta Q_{1} = \left( {\left[ \begin{gathered} \left( {\frac{{\left( {1 + \left( {\mu_{1}^{L} } \right)^{p} } \right)^{\eta } - \left( {1 - \left( {\mu_{1}^{L} } \right)^{p} } \right)^{\eta } }}{{\left( {1 + \left( {\mu_{1}^{L} } \right)^{p} } \right)^{\eta } + \left( {1 - \left( {\mu_{1}^{L} } \right)^{p} } \right)^{\eta } }}} \right)^{\frac{1}{p}} \hfill \\ \left( {\frac{{\left( {1 + \left( {\mu_{1}^{U} } \right)^{p} } \right)^{\eta } - \left( {1 - \left( {\mu_{1}^{U} } \right)^{p} } \right)^{\eta } }}{{\left( {1 + \left( {\mu_{1}^{U} } \right)^{p} } \right)^{\eta } + \left( {1 - \left( {\mu_{1}^{U} } \right)^{p} } \right)^{\eta } }}} \right)^{\frac{1}{p}} \hfill \\ \end{gathered} \right],\left[ \begin{gathered} \left( {\frac{{2\left( {\nu_{1}^{L} } \right)^{qn} }}{{\left( {2 - \left( {\nu_{1}^{L} } \right)^{q} } \right)^{\eta } + \left( {\nu_{1}^{L} } \right)^{qn} }}} \right)^{\frac{1}{q}} , \hfill \\ \left( {\frac{{2\left( {\nu_{1}^{U} } \right)^{qn} }}{{\left( {2 - \left( {\nu_{1}^{U} } \right)^{q} } \right)^{\eta } + \left( {\nu_{1}^{U} } \right)^{qn} }}} \right)^{\frac{1}{q}} \hfill \\ \end{gathered} \right]} \right)$$
  4. $$\left( {Q_{1} } \right)^{\eta } = \left( {\left[ \begin{gathered} \left( {\frac{{2\left( {\mu_{1}^{L} } \right)^{pn} }}{{\left( {2 - \left( {\mu_{1}^{L} } \right)^{p} } \right)^{\eta } + \left( {\mu_{1}^{L} } \right)^{pn} }}} \right)^{\frac{1}{p}} , \hfill \\ \left( {\frac{{2\left( {\mu_{1}^{U} } \right)^{pn} }}{{\left( {2 - \left( {\mu_{1}^{U} } \right)^{p} } \right)^{\eta } + \left( {\mu_{1}^{U} } \right)^{pn} }}} \right)^{\frac{1}{p}} \hfill \\ \end{gathered} \right],\left[ \begin{gathered} \left( {\frac{{\left( {1 + \left( {\nu_{1}^{L} } \right)^{q} } \right)^{\eta } - \left( {1 - \left( {\nu_{1}^{L} } \right)^{q} } \right)^{\eta } }}{{\left( {1 + \left( {\nu_{1}^{L} } \right)^{q} } \right)^{\eta } + \left( {1 - \left( {\nu_{1}^{L} } \right)^{q} } \right)^{\eta } }}} \right)^{\frac{1}{q}} \hfill \\ \left( {\frac{{\left( {1 + \left( {\nu_{1}^{U} } \right)^{q} } \right)^{\eta } - \left( {1 - \left( {\nu_{1}^{U} } \right)^{q} } \right)^{\eta } }}{{\left( {1 + \left( {\nu_{1}^{U} } \right)^{q} } \right)^{\eta } + \left( {1 - \left( {\nu_{1}^{U} } \right)^{q} } \right)^{\eta } }}} \right)^{\frac{1}{q}} \hfill \\ \end{gathered} \right]} \right)$$
3. If $$g\left( x \right) = \log \left( {\frac{{\gamma + \left( {1 - \gamma } \right)x^{p} }}{{x^{p} }}} \right)$$ and $$h\left( x \right) = \log \left( {\frac{{\gamma + \left( {1 - \gamma } \right)\left( {1 - x^{q} } \right)}}{{1 - x^{q} }}} \right)$$, the operational laws for the IVPQ-QOFNs based on Hamacher TN and TCN can be defined as follows:
  1. $$Q_{1} \oplus Q_{2} = \left( {\left[ \begin{gathered} \left( {\frac{{\left( {\mu_{1}^{L} } \right)^{p} + \left( {\mu_{2}^{L} } \right)^{p} - \left( {\mu_{1}^{L} } \right)^{p} \left( {\mu_{2}^{L} } \right)^{p} - \left( {1 - \gamma } \right)\left( {\mu_{1}^{L} } \right)^{p} \left( {\mu_{2}^{L} } \right)^{p} }}{{1 - \left( {1 - \gamma } \right)\left( {\mu_{1}^{L} } \right)^{p} \left( {\mu_{2}^{L} } \right)^{p} }}} \right)^{\frac{1}{p}} \hfill \\ ,\left( {\frac{{\left( {\mu_{1}^{U} } \right)^{p} + \left( {\mu_{2}^{U} } \right)^{p} - \left( {\mu_{1}^{U} } \right)^{p} \left( {\mu_{2}^{U} } \right)^{p} - \left( {1 - \gamma } \right)\left( {\mu_{1}^{U} } \right)^{p} \left( {\mu_{2}^{U} } \right)^{p} }}{{1 - \left( {1 - \gamma } \right)\left( {\mu_{1}^{U} } \right)^{p} \left( {\mu_{2}^{U} } \right)^{p} }}} \right)^{\frac{1}{p}} \hfill \\ \end{gathered} \right],\left[ \begin{gathered} \left( {\frac{{\left( {\nu_{1}^{L} } \right)^{q} \left( {\nu_{2}^{L} } \right)^{q} }}{{\gamma + \left( {1 - \gamma } \right)\left( {\left( {\nu_{1}^{L} } \right)^{q} + \left( {\nu_{2}^{L} } \right)^{q} - \left( {\nu_{1}^{L} } \right)^{q} \left( {\nu_{2}^{L} } \right)^{q} } \right)}}} \right)^{\frac{1}{q}} \hfill \\ ,\left( {\frac{{\left( {\nu_{1}^{U} } \right)^{q} \left( {\nu_{2}^{U} } \right)^{q} }}{{\gamma + \left( {1 - \gamma } \right)\left( {\left( {\nu_{1}^{U} } \right)^{q} + \left( {\nu_{2}^{U} } \right)^{q} - \left( {\nu_{1}^{U} } \right)^{q} \left( {\nu_{2}^{U} } \right)^{q} } \right)}}} \right)^{\frac{1}{q}} \hfill \\ \end{gathered} \right]} \right)$$.
  2. $$Q_{1} \otimes Q_{2} = \left( {\left[ \begin{gathered} \left( {\frac{{\left( {\mu_{1}^{L} } \right)^{p} \left( {\mu_{2}^{L} } \right)^{p} }}{{\gamma + \left( {1 - \gamma } \right)\left( {\left( {\mu_{1}^{L} } \right)^{p} + \left( {\mu_{2}^{L} } \right)^{p} - \left( {\mu_{1}^{L} } \right)^{p} \left( {\mu_{2}^{L} } \right)^{p} } \right)}}} \right)^{\frac{1}{p}} \hfill \\ ,\left( {\frac{{\left( {\mu_{1}^{U} } \right)^{p} \left( {\mu_{2}^{U} } \right)^{p} }}{{\gamma + \left( {1 - \gamma } \right)\left( {\left( {\mu_{1}^{U} } \right)^{p} + \left( {\mu_{2}^{U} } \right)^{p} - \left( {\mu_{1}^{U} } \right)^{p} \left( {\mu_{2}^{U} } \right)^{p} } \right)}}} \right)^{\frac{1}{p}} \hfill \\ \end{gathered} \right],\left[ \begin{gathered} \left( {\frac{{\left( {\nu_{1}^{L} } \right)^{q} + \left( {\nu_{2}^{L} } \right)^{q} - \left( {\nu_{1}^{L} } \right)^{q} \left( {\nu_{2}^{L} } \right)^{q} - \left( {1 - \gamma } \right)\left( {\nu_{1}^{L} } \right)^{q} \left( {\nu_{2}^{L} } \right)^{q} }}{{1 - \left( {1 - \gamma } \right)\left( {\nu_{1}^{L} } \right)^{q} \left( {\nu_{2}^{L} } \right)^{q} }}} \right)^{\frac{1}{q}} \hfill \\ ,\left( {\frac{{\left( {\nu_{1}^{U} } \right)^{q} + \left( {\nu_{2}^{U} } \right)^{q} - \left( {\nu_{1}^{U} } \right)^{q} \left( {\nu_{2}^{U} } \right)^{q} - \left( {1 - \gamma } \right)\left( {\nu_{1}^{U} } \right)^{q} \left( {\nu_{2}^{U} } \right)^{q} }}{{1 - \left( {1 - \gamma } \right)\left( {\nu_{1}^{U} } \right)^{q} \left( {\nu_{2}^{U} } \right)^{q} }}} \right)^{\frac{1}{q}} \hfill \\ \end{gathered} \right]} \right)$$
  3. $$\eta Q_{1} = \left( {\left[ \begin{gathered} \left( {\frac{{\left( {1 + \left( {\gamma - 1} \right)\left( {\mu_{1}^{L} } \right)^{p} } \right)^{\eta } - \left( {1 - \left( {\mu_{1}^{L} } \right)^{p} } \right)^{\eta } }}{{\left( {1 + \left( {\gamma - 1} \right)\left( {\mu_{1}^{L} } \right)^{p} } \right)^{\eta } + \left( {\gamma - 1} \right)\left( {1 - \left( {\mu_{1}^{L} } \right)^{p} } \right)^{\eta } }}} \right)^{\frac{1}{p}} , \hfill \\ \left( {\frac{{\left( {1 + \left( {\gamma - 1} \right)\left( {\mu_{1}^{U} } \right)^{p} } \right)^{\eta } - \left( {1 - \left( {\mu_{1}^{U} } \right)^{p} } \right)^{\eta } }}{{\left( {1 + \left( {\gamma - 1} \right)\left( {\mu_{1}^{U} } \right)^{p} } \right)^{\eta } + \left( {\gamma - 1} \right)\left( {1 - \left( {\mu_{1}^{U} } \right)^{p} } \right)^{\eta } }}} \right)^{\frac{1}{p}} \hfill \\ \end{gathered} \right],\left[ \begin{gathered} \left( {\frac{{\gamma \left( {\nu_{1}^{L} } \right)^{q\eta } }}{{\left( {\gamma - 1} \right)\left( {\nu_{1}^{L} } \right)^{q\eta } + \left( {1 + \left( {\gamma - 1} \right)\left( {1 - \left( {\nu_{1}^{L} } \right)^{q} } \right)} \right)^{\eta } }}} \right)^{\frac{1}{q}} \hfill \\ ,\left( {\frac{{\gamma \left( {\nu_{1}^{U} } \right)^{q\eta } }}{{\left( {\gamma - 1} \right)\left( {\nu_{1}^{U} } \right)^{q\eta } + \left( {1 + \left( {\gamma - 1} \right)\left( {1 - \left( {\nu_{1}^{U} } \right)^{q} } \right)} \right)^{\eta } }}} \right)^{\frac{1}{q}} \hfill \\ \end{gathered} \right]} \right)$$
  4. $$\left( {Q_{1} } \right)^{\eta } = \left( {\left[ \begin{gathered} \left( {\frac{{\gamma \left( {\mu_{1}^{L} } \right)^{p\eta } }}{{\left( {\gamma - 1} \right)\left( {\mu_{1}^{L} } \right)^{p\eta } + \left( {1 + \left( {\gamma - 1} \right)\left( {1 - \left( {\mu_{1}^{L} } \right)^{p} } \right)} \right)^{\eta } }}} \right)^{\frac{1}{p}} \hfill \\ ,\left( {\frac{{\gamma \left( {\mu_{1}^{U} } \right)^{p\eta } }}{{\left( {\gamma - 1} \right)\left( {\mu_{1}^{U} } \right)^{p\eta } + \left( {1 + \left( {\gamma - 1} \right)\left( {1 - \left( {\mu_{1}^{U} } \right)^{p} } \right)} \right)^{\eta } }}} \right)^{\frac{1}{p}} \hfill \\ \end{gathered} \right],\left[ \begin{gathered} \left( {\frac{{\left( {1 + \left( {\gamma - 1} \right)\left( {\nu_{1}^{L} } \right)^{q} } \right)^{\eta } - \left( {1 - \left( {\nu_{1}^{L} } \right)^{q} } \right)^{\eta } }}{{\left( {1 + \left( {\gamma - 1} \right)\left( {\nu_{1}^{L} } \right)^{q} } \right)^{\eta } + \left( {\gamma - 1} \right)\left( {1 - \left( {\nu_{1}^{L} } \right)^{q} } \right)^{\eta } }}} \right)^{\frac{1}{q}} , \hfill \\ \left( {\frac{{\left( {1 + \left( {\gamma - 1} \right)\left( {\nu_{1}^{U} } \right)^{q} } \right)^{\eta } - \left( {1 - \left( {\nu_{1}^{U} } \right)^{q} } \right)^{\eta } }}{{\left( {1 + \left( {\gamma - 1} \right)\left( {\nu_{1}^{U} } \right)^{q} } \right)^{\eta } + \left( {\gamma - 1} \right)\left( {1 - \left( {\nu_{1}^{U} } \right)^{q} } \right)^{\eta } }}} \right)^{\frac{1}{q}} \hfill \\ \end{gathered} \right]} \right).$$
4. If $$g\left( x \right) = - \log \left( {\frac{\tau - 1}{{\tau^{{x^{p} }} - 1}}} \right)$$ and $$h\left( x \right) = - \log \left( {\frac{\tau - 1}{{\tau^{{1 - x^{q} }} - 1}}} \right)$$, the operational laws for the IVPQ-ROFNs based on Frank TN and TCN can be defined as follows:
  1. $$Q_{1} \oplus Q_{2} = \left( {\left[ \begin{gathered} \left( {1 - \log_{\tau } \left( {1 + \frac{{\left( {\tau^{{1 - \left( {\mu_{1}^{L} } \right)^{p} }} - 1} \right)\left( {\tau^{{1 - \left( {\mu_{2}^{L} } \right)^{p} }} - 1} \right)}}{\tau - 1}} \right)} \right)^{\frac{1}{p}} , \hfill \\ \left( {1 - \log_{\tau } \left( {1 + \frac{{\left( {\tau^{{1 - \left( {\mu_{1}^{L} } \right)^{p} }} - 1} \right)\left( {\tau^{{1 - \left( {\mu_{2}^{L} } \right)^{p} }} - 1} \right)}}{\tau - 1}} \right)} \right)^{\frac{1}{p}} \hfill \\ \end{gathered} \right],\left[ \begin{gathered} \left( {\log_{\tau } \left( {1 + \frac{{\left( {\tau^{{\left( {\nu_{1}^{L} } \right)^{q} }} - 1} \right)\left( {\tau^{{\left( {\nu_{2}^{L} } \right)^{q} }} - 1} \right)}}{\tau - 1}} \right)} \right)^{\frac{1}{q}} , \hfill \\ \left( {\log_{\tau } \left( {1 + \frac{{\left( {\tau^{{\left( {\nu_{1}^{U} } \right)^{q} }} - 1} \right)\left( {\tau^{{\left( {\nu_{2}^{U} } \right)^{q} }} - 1} \right)}}{\tau - 1}} \right)} \right)^{\frac{1}{q}} \hfill \\ \end{gathered} \right]} \right)$$.
  2. $$Q_{1} \otimes Q_{2} = \left( {\left[ \begin{gathered} \left( {\log_{\tau } \left( {1 + \frac{{\left( {\tau^{{\left( {\mu_{1}^{L} } \right)^{p} }} - 1} \right)\left( {\tau^{{\left( {\mu_{2}^{L} } \right)^{p} }} - 1} \right)}}{\tau - 1}} \right)} \right)^{\frac{1}{p}} , \hfill \\ \left( {\log_{\tau } \left( {1 + \frac{{\left( {\tau^{{\left( {\mu_{1}^{U} } \right)^{p} }} - 1} \right)\left( {\tau^{{\left( {\mu_{2}^{U} } \right)^{p} }} - 1} \right)}}{\tau - 1}} \right)} \right)^{\frac{1}{p}} \hfill \\ \end{gathered} \right],\left[ \begin{gathered} \left( {1 - \log_{\tau } \left( {1 + \frac{{\left( {\tau^{{1 - \left( {\nu_{1}^{L} } \right)^{q} }} - 1} \right)\left( {\tau^{{1 
- \left( {\nu_{2}^{L} } \right)^{q} }} - 1} \right)}}{\tau - 1}} \right)} \right)^{\frac{1}{q}} , \hfill \\ \left( {1 - \log_{\tau } \left( {1 + \frac{{\left( {\tau^{{1 - \left( {\nu_{1}^{U} } \right)^{q} }} - 1} \right)\left( {\tau^{{1 - \left( {\nu_{2}^{U} } \right)^{q} }} - 1} \right)}}{\tau - 1}} \right)} \right)^{\frac{1}{q}} \hfill \\ \end{gathered} \right]} \right)$$.
  3. $$\eta Q_{1} = \left( {\left[ \begin{gathered} \left( {1 - \log_{\tau } \left( {1 + \frac{{\left( {\tau^{{1 - \left( {\mu_{1}^{L} } \right)^{p} }} - 1} \right)^{\eta } }}{{\left( {\tau - 1} \right)^{\eta - 1} }}} \right)} \right)^{\frac{1}{p}} , \hfill \\ \left( {1 - \log_{\tau } \left( {1 + \frac{{\left( {\tau^{{1 - \left( {\mu_{1}^{U} } \right)^{p} }} - 1} \right)^{\eta } }}{{\left( {\tau - 1} \right)^{\eta - 1} }}} \right)} \right)^{\frac{1}{p}} \hfill \\ \end{gathered} \right],\left[ \begin{gathered} \left( {\log_{\tau } \left( {1 + \frac{{\left( {\tau^{{\left( {\nu_{1}^{L} } \right)^{q} }} - 1} \right)^{\eta } }}{{\left( {\tau - 1} \right)^{\eta - 1} }}} \right)} \right)^{\frac{1}{q}} , \hfill \\ \left( {\log_{\tau } \left( {1 + \frac{{\left( {\tau^{{\left( {\nu_{1}^{U} } \right)^{q} }} - 1} \right)^{\eta } }}{{\left( {\tau - 1} \right)^{\eta - 1} }}} \right)} \right)^{\frac{1}{q}} , \hfill \\ \end{gathered} \right]} \right)$$.
  4. $$\left( {Q_{1} } \right)^{\eta } = \left( {\left[ \begin{gathered} \left( {\log_{\tau } \left( {1 + \frac{{\left( {\tau^{{\left( {\mu_{1}^{L} } \right)^{p} }} - 1} \right)^{\eta } }}{{\left( {\tau - 1} \right)^{\eta - 1} }}} \right)} \right)^{\frac{1}{p}} , \hfill \\ \left( {\log_{\tau } \left( {1 + \frac{{\left( {\tau^{{\left( {\mu_{1}^{U} } \right)^{p} }} - 1} \right)^{\eta } }}{{\left( {\tau - 1} \right)^{\eta - 1} }}} \right)} \right)^{\frac{1}{p}} , \hfill \\ \end{gathered} \right],\left[ \begin{gathered} \left( {1 - \log_{\tau } \left( {1 + \frac{{\left( {\tau^{{1 - \left( {\nu_{1}^{L} } \right)^{q} }} - 1} \right)^{\eta } }}{{\left( {\tau - 1} \right)^{\eta - 1} }}} \right)} \right)^{\frac{1}{q}} , \hfill \\ \left( {1 - \log_{\tau } \left( {1 + \frac{{\left( {\tau^{{1 - \left( {\nu_{1}^{U} } \right)^{p} }} - 1} \right)^{\eta } }}{{\left( {\tau - 1} \right)^{\eta - 1} }}} \right)} \right)^{\frac{1}{q}} \hfill \\ \end{gathered} \right]} \right)$$.

### Example 3.1

Let $Q_{1} = \left( {[0.2,0.3],[0.4,0.5]} \right)$ and $Q_{2} = \left( {[0.4,0.5],[0.3,0.5]} \right)$ be two IV.

PQ-ROFNS, $\eta = 2$ and $p = q = 3$. According to the operational laws for the IVPQ-ROFNs based on Algebraic TN and TCN, $Q_{1} \oplus Q_{2}$, $Q_{1} \otimes Q_{2}$, $\eta Q_{1}$ and $\left( {Q_{1} } \right)^{\eta }$ can be computed as follows:

1. $$Q_{1} \oplus Q_{2} = \left( {[0.4150,0.5297],[0.1200,0.2500]} \right)$$
2. $$Q_{1} \otimes Q_{2} = ([0.0800,0.1500],[0.4469,0.6166])$$
3. $$\eta Q_{1} = ([0.2516,0.3763],[0.1600,0.2500])$$
4. $$\left( {Q_{1} } \right)^{\eta } = ([0.0400],[0.0900],[0.4985,0.6166])$$

### Example 3.2

Let $Q_{1} = \left( {[0.2,0.3],[0.4,0.5]} \right)$ and $Q_{2} = \left( {[0.4,0.5],[0.3,0.5]} \right)$ be two IV.

PQ-ROFNS, $\eta = 2$ and $p = q = 3$. According to the operational laws for the IVPQ-ROFNs based on Einstein TN and TCN, $Q_{1} \oplus Q_{2}$, $Q_{1} \otimes Q_{2}$, $\eta Q_{1}$ and $\left( {Q_{1} } \right)^{\eta }$ can be computed as follows:

1. $$Q_{1} \oplus Q_{2} = ([0.4159,0.5331],[0.0967,0.2068])$$
2. $$Q_{1} \otimes Q_{2} = ([0.0643,0.1222],[0.4495,0.6267])$$
3. $$\eta Q_{1} = ([0.2520,0.3779],[0.0053,0.0205])$$
4. $$\left( {Q_{1} } \right)^{\eta } = ([0.0318,0.0721],[0.2029,0.3948])$$

### Example 3.3

Let $Q_{1} = \left( {[0.2,0.3],[0.4,0.5]} \right)$ and $Q_{2} = \left( {[0.4,0.5],[0.3,0.5]} \right)$ be two IV.

PQ-ROFNS, $\eta = 2$ and $p = q = 3$. According to the operational laws for the IVPQ-ROFNs based on Hamacher TN and TCN, $Q_{1} \oplus Q_{2}$, $Q_{1} \otimes Q_{2}$, $\eta Q_{1}$ and $\left( {Q_{1} } \right)^{\eta }$ can be computed when taking different parameter $\gamma$, and the results are shown in Table 3.

**Table 3:** The calculation results when taking different parameter $\gamma$.

$\gamma$	$Q_{1} \oplus Q_{2}$	$Q_{1} \otimes Q_{2}$	$\eta Q_{1}$	$\left( {Q_{1} } \right)^{\eta }$
$\gamma = 1$	([0.4150,0.5297], [0.1200,0.2500])	([0.0800,0.0800,], [0.4469,0.6166])	([0.0400,0.0900], [0.4985,0.6166])	([0.2516,0.3763], [0.1600,0.2500])
$\gamma = 2$	([0.4159,0.5331], [0.0967,0.2068])	([0.0643,0.0643], [0.4495,0.6267])	([0.0318,0.0721], [0.5033,0.6267])	([0.2520,0.3779], [0.1297,0.2068])
$\gamma = 3$	([0.4169,0.5364], [0.0849,0.1834])	([0.0564,0.0564], [0.4521,0.6363])	([0.0278,0.0632], [0.5079,0.6363])	([0.2523,0.3795], [0.1142,0.1834])
$\gamma = 4$	([0.4178,0.5397], [0.0774,0.1680])	([0.0513,0.0513,] [0.4546,0.6453])	([0.0253,0.0575], [0.5124,0.6453])	([0.2526,0.3811], [0.1041,0.1680])
$\gamma = 5$	([0.4187,0.5428], [0.0719,0.1567])	([0.0477,0.0477], [0.4571,0.6538])	([0.0235,0.0534], [0.5168,0.6538])	([0.2530,0.3826], [0.0969,0.1567])

### Example 3.4

Let $Q_{1} = \left( {[0.2,0.3],[0.4,0.5]} \right)$ and $Q_{2} = \left( {[0.4,0.5],[0.3,0.5]} \right)$ be two IV.

PQ-ROFNS, $\eta = 2$ and $p = q = 3$. According to the operational laws for the IVPQ-ROFNs based on Frank TN and TCN, $Q_{1} \oplus Q_{2}$, $Q_{1} \otimes Q_{2}$, $\eta Q_{1}$ and $\left( {Q_{1} } \right)^{\eta }$ can be computed when taking different parameter $\tau$, and the results are shown in Table 4.

**Table 4:** The calculation results when taking different parameter $\tau$.

$\tau$	$Q_{1} \oplus Q_{2}$	$Q_{1} \otimes Q_{2}$	$\eta Q_{1}$	$\left( {Q_{1} } \right)^{\eta }$
$\tau = 1.01$	([0.4150,0.5297], [0.1200,0.2500])	([0.0800,0.0800,], [0.4469,0.6166])	([0.0400,0.0900], [0.4985,0.6166])	([0.2516,0.3763], [0.1600,0.2500])
$\tau = 2.01$	([0.4153,0.5308], [0.1072,0.2273])	([0.0713,0.1350], [0.4478,]0.6199)	([0.2518,0.3768], [0.1435,0.2273])	([0.0354,0.0801], [0.5001,0.6199])
$\tau = 3.01$	([0.4155,0.5313], [0.0999,0.2140])	([0.0663,0.1262], [0.4481,0.6216])	([0.2518,0.3770], [0.1340,0.2140])	([0.0328,0.0744], [0.5008,0.6216])
$\tau = 4.01$	([0.4155,0.5316], [0.0947,0.2045])	([0.0629,0.1201], [0.4484,0.6227])	([0.2518,0.3772], [0.1273,0.2045])	([0.0310,0.0704], [0.5012,0.6227])
$\tau = 5.01$	([0.4156,0.5319], [0.0907,0.1971])	([0.0602,0.1153], [0.4486,0.6235])	([0.2518,0.3773], [0.1222,0.1971])	([0.0296,0.0674], [0.5016,0.6235])

According to the results of Examples 3.1–3.3, it can be seen that the result obtained based on the Hamacher operation law is consistent with the result obtained based on the Algebraic operation law when $\gamma$ is equal to 1, and consistent with the result obtained based on the Einstein operation law when $\gamma$ is equal to 2. This is because the Hamacher operation law degenerate into the Algebraic operation law when $\gamma$ is equal to 1, and the Einstein operation law when $\gamma$ is equal to 2. Therefore, compared to the Algebraic operation law and the Einstein operation law, the Hamacher operation law is a more general form, while the Algebraic operation law and the Einstein operation law are its special forms.

Based on the results of Examples 3.1 and 3.4, it can be seen that the result obtained based on the Frank operation law is consistent with the result obtained based on the Algebraic operation law when $\tau$ approaches 1. This is mainly because when $\tau$ approaches 1, the Frank operation law can be transformed into Algebraic operation law.

$$ \mathop {\lim }\limits_{\tau \to 1} \log \left( {\frac{\tau - 1}{{\tau^{{x^{p} }} - 1}}} \right) = \mathop {\lim }\limits_{\tau \to 1} \log \left( {\frac{{\exp \left\{ {\log \tau } \right\} - 1}}{{\exp \left\{ {x^{p} \log \tau } \right\} - 1}}} \right) = \mathop {\lim }\limits_{\tau \to 1} \log \left( {\frac{\log \tau }{{x^{p} \log \tau }}} \right) = - \log x^{p} $$

$$ \mathop {\lim }\limits_{\tau \to 1} \log \left( {\frac{\tau - 1}{{\tau^{{1 - x^{q} }} - 1}}} \right) = \mathop {\lim }\limits_{\tau \to 1} \log \left( {\frac{{\exp \left\{ {\log \tau } \right\} - 1}}{{\exp \left\{ {\left( {1 - x^{q} } \right)\log \tau } \right\} - 1}}} \right) = \mathop {\lim }\limits_{\tau \to 1} \log \left( {\frac{\log \tau }{{\left( {1 - x^{q} } \right)\log \tau }}} \right) = - \log \left( {1 - x^{q} } \right) $$

Based on the above analysis, we can conclude that the Hamacher operation law and the Frank operation law have better flexibility due to the presence of parameters, which are mainly set according to the preferences of DMs and the characteristics of the input data. However, Algebraic operation law is the most widely used in practical applications due to their simplicity and ease of operation.

## Some GIVPQ-ROFMSM operators based on ATN and ATCN

In this section, we propose some aggregation operators based on ATN, ATCN and GMSM operator under IVPQ-ROF environment**.**

### GIVPQ-ROFMSM operator

In this part, we extend the GMSM operator into IVPQ-ROF environment and propose an GIVPQ-ROFMSM operator by utilizing operational rules of IVPQ-ROFN based on ATN and ATCN. Moreover, some valuable properties of the GIVPQ-QOFMSM operators are studied.

#### Definition 4.1

Let $Q = \left( {\left[ {\mu_{i}^{L} ,\mu_{i}^{U} } \right],\left[ {\nu_{i}^{L} ,\nu_{i}^{U} } \right]} \right)\left( {i = 1,2,...,n} \right)$ be IVPQ-ROFNs, $p,q \ge 1$ The GIVPQ-ROFMSM operator of the IVPQ-ROFNs $Q_{i} \left( {i = 1,2,...,n} \right)$ can be defined as follows:

8 $$ GIVPQ - ROFMSM^{{\left( {k,\lambda_{1} ,\lambda_{2} ,...,\lambda_{k} } \right)}} \left( {Q_{1} ,Q_{2} ,...,Q_{n} } \right) = \left( {\frac{1}{{C_{n}^{k} }}\mathop \oplus \limits_{{1 \le i_{1} < ... < i_{k} \le n}} \left( {\mathop \otimes \limits_{j = 1}^{k} Q_{{i_{j} }}^{{\lambda_{j} }} } \right)} \right)^{{\frac{1}{{\lambda_{1} + \lambda_{2} + ... + \lambda_{k} }}}} $$

where $\lambda_{1} ,\lambda_{2} ,...,\lambda_{k} \ge 0$, $k$ is a parameter and $k = 1,2,...,n$. $\left( {i_{1} ,i_{2} ,...,i_{k} } \right)$ traverses all the n-tuple combination of $\left( {1,2,...,n} \right)$, and $C_{n}^{k} = \frac{n!}{{k!\left( {n - k} \right)!}}$ is the binomial coefficient.

#### Theorem 4.1

Let $Q = \left( {\left[ {\mu_{i}^{L} ,\mu_{i}^{U} } \right],\left[ {\nu_{i}^{L} ,\nu_{i}^{U} } \right]} \right)\left( {i = 1,2,...,n} \right)$ be IVPQ-ROFNs, $\diamondsuit = \left\{ {U,L} \right\}$ $p,q \ge 1$. Then

9 $$ GIVPQ - ROFMSM^{{\left( {k,\lambda_{1} ,\lambda_{2} ,...,\lambda_{k} } \right)}} \left( {Q_{1} ,Q_{2} ,...,Q_{n} } \right) = \left( {\left[ {A^{L} ,A^{U} } \right],\left[ {B^{L} ,B^{U} } \right]} \right) $$

where

$$ A^{\diamondsuit } = g^{ - 1} \left( {\frac{1}{{\lambda_{1} + \lambda_{2} + ... + \lambda_{k} }}g\left( {h^{ - 1} \left( {\frac{1}{{C_{n}^{k} }}\sum\limits_{{1 \le i_{1} < ... < i_{k} \le n}} {h\left( {g^{ - 1} \left( {\sum\limits_{j = 1}^{k} {\lambda_{j} g\left( {\mu_{{i_{j} }}^{\diamondsuit } } \right)} } \right)} \right)} } \right)} \right)} \right) $$

$$ B^{\diamondsuit } = h^{ - 1} \left( {\frac{1}{{\lambda_{1} + \lambda_{2} + ... + \lambda_{k} }}h\left( {g^{ - 1} \left( {\frac{1}{{C_{n}^{k} }}\sum\limits_{{1 \le i_{1} < ... < i_{k} \le n}} {g\left( {h^{ - 1} \left( {\sum\limits_{j = 1}^{k} {\lambda_{j} h\left( {\nu_{{i_{j} }}^{\diamondsuit } } \right)} } \right)} \right)} } \right)} \right)} \right) $$

#### Proof

See appendix A.

#### Property 4.1

Let $Q_{i} = \left( {\left[ {\mu_{i}^{L} ,\mu_{i}^{U} } \right],\left[ {\nu_{i}^{L} ,\nu_{i}^{U} } \right]} \right)\left( {i = 1,2,...,n} \right)$ and $Q_{i}^{*} = \left( {\left[ {\mu_{i}^{L*} ,\mu_{i}^{U*} } \right],\left[ {\nu_{i}^{L*} ,\nu_{i}^{U*} } \right]} \right)\left( {i = 1,2,...,n} \right)$ be two collections of IVPQ-ROFNs, $\diamondsuit = \left\{ {U,L} \right\}$ and $p,q \ge 1$. Then

1. Idempotency: If $Q_{i} = \left( {\left[ {\mu^{L} ,\mu^{U} } \right],\left[ {\nu^{L} ,\nu^{U} } \right]} \right)\left( {i = 1,2,...,n} \right)$ for all $i = 1,2,...,n$, then
  $$ GIVPQ - ROFMSM^{{\left( {k,\lambda_{1} ,\lambda_{2} ,...,\lambda_{k} } \right)}} \left( {Q_{1} ,Q_{2} ,...,Q_{n} } \right) = \left( {\left[ {\mu^{L} ,\mu^{U} } \right],\left[ {\nu^{L} ,\nu^{U} } \right]} \right) $$
2. Monotonicity: If $\mu_{i}^{U} \le \mu_{i}^{U*}$, $\mu_{i}^{L} \le \mu_{i}^{L*}$, $\nu_{i}^{U} \ge \nu_{i}^{U*}$ and $\nu_{i}^{L} \ge \nu_{i}^{L*}$ for all $i = 1,2,...,n$, then
  $$ GIVPQ - ROFMSM^{{\left( {k,\lambda_{1} ,\lambda_{2} ,...,\lambda_{k} } \right)}} \left( {Q_{1} ,Q_{2} ,...,Q_{n} } \right) \le GIVPQ - ROFMSM^{{\left( {k,\lambda_{1} ,\lambda_{2} ,...,\lambda_{k} } \right)}} \left( {Q_{1}^{*} ,Q_{2}^{*} ,...,Q_{n}^{*} } \right) $$
3. Boundedness: $Q^{ - } \le GIVPQ - ROFMSM^{{\left( {k,\lambda_{1} ,\lambda_{2} ,...,\lambda_{k} } \right)}} \left( {Q_{1} ,Q_{2} ,...,Q_{n} } \right) \le Q^{ + }$, where $Q^{ + } = \left( {\left[ {\mathop {\max }\limits_{i} \left\{ {\mu_{i}^{L} } \right\},\mathop {\max }\limits_{i} \left\{ {\mu_{i}^{U} } \right\}} \right],\left[ {\mathop {\min }\limits_{i} \left\{ {\nu_{i}^{L} } \right\},\mathop {\min }\limits_{i} \left\{ {\nu_{i}^{U} } \right\}} \right]} \right)$ and $Q^{ - } = \left( {\left[ {\mathop {\min }\limits_{i} \left\{ {\mu_{i}^{L} } \right\},\mathop {\min }\limits_{i} \left\{ {\mu_{i}^{U} } \right\}} \right],\left[ {\mathop {\max }\limits_{i} \left\{ {\nu_{i}^{L} } \right\},\mathop {\max }\limits_{i} \left\{ {\nu_{i}^{U} } \right\}} \right]} \right)$.

#### Proof

See appendix B.

### Some special cases of GIVPQ-ROFMSM operator

In this part, we will obtain some special cases of GIVPQ-ROFMSM operator when setting different values for parameters, such as: interval valued p,q-Rung orthopair fuzzy Bonferroni mean (IVPQ-ROFBM) operator, interval valued p,q-Rung orthopair fuzzy Maclaurin symmetric mean (IVPQ-ROFMSM) operator, interval valued p,q-Rung orthopair fuzzy Hamy mean (IVPQ-ROFHM) operator. Further, we also introduce some special cases of the GIVPQ-ROFMSM operator when taking different additive generators.

When setting different values of parameters, we have such special cases:

1. When $k = 2$ and $\lambda_{1} = \lambda_{2} = \lambda$, the GIVPQ-ROFMSM operator is simplified into the IVPQ-ROFBM operator, shown as follows:
  10 $$ \begin{gathered} IVPQ - ROFBM^{{\left( {2,\lambda ,\lambda } \right)}} \left( {Q_{1} ,Q_{2} ,...,Q_{n} } \right) \\ = \left( {\frac{1}{{n\left( {n - 1} \right)}}\mathop \oplus \limits_{\begin{subarray}{l} i,j = 1 \\ i \ne j \end{subarray} }^{n} \left( {Q_{i}^{\lambda } \otimes Q_{j}^{\lambda } } \right)} \right)^{{\frac{1}{2\lambda }}} \\ = \left( {\left[ {A^{L} ,A^{U} } \right],\left[ {B^{L} ,B^{U} } \right]} \right) \\ \end{gathered} $$
  where
  $$ A^{\diamondsuit } = g^{ - 1} \left( {\frac{1}{2\lambda }g\left( {h^{ - 1} \left( {\frac{1}{{n\left( {n - 1} \right)}}\sum\limits_{\begin{subarray}{l} i,j = 1 \\ i \ne j \end{subarray} }^{n} {h\left( {g^{ - 1} \left( {\lambda g\left( {\mu_{i}^{\diamondsuit } } \right) + \lambda g\left( {\mu_{j}^{\diamondsuit } } \right)} \right)} \right)} } \right)} \right)} \right) $$
  $$ B^{\diamondsuit } = h^{ - 1} \left( {\frac{1}{2\lambda }h\left( {g^{ - 1} \left( {\frac{1}{{n\left( {n - 1} \right)}}\sum\limits_{\begin{subarray}{l} i,j = 1 \\ i \ne j \end{subarray} }^{n} {g\left( {h^{ - 1} \left( {\lambda h\left( {\nu_{i}^{\diamondsuit } } \right) + \lambda h\left( {x_{j}^{\diamondsuit } } \right)} \right)} \right)} } \right)} \right)} \right) $$
2. When $\lambda_{1} = \lambda_{2} = ... = \lambda_{k} = 1$, the GIVPQ-ROFMSM operator is transformed into the IVPQ-ROFMSM operator, shown as follows:
  11 $$ \begin{gathered} IVPQ - ROFMSM^{\left( k \right)} \left( {Q_{1} ,Q_{2} ,...,Q_{n} } \right) \hfill \\ = \left( {\frac{1}{{C_{n}^{k} }}\mathop \oplus \limits_{{1 \le i_{1} < ... < i_{k} \le n}} \left( {\mathop \otimes \limits_{j = 1}^{k} Q_{{i_{j} }} } \right)} \right)^{\frac{1}{k}} \hfill \\ = \left( {\left[ {A^{L} ,A^{U} } \right],\left[ {B^{L} ,B^{U} } \right]} \right) \hfill \\ \end{gathered} $$
  where
  $$ A^{\diamondsuit } = g^{ - 1} \left( {\frac{1}{k}g\left( {h^{ - 1} \left( {\frac{1}{{C_{n}^{k} }}\sum\limits_{{1 \le i_{1} < ... < i_{k} \le n}} {h\left( {g^{ - 1} \left( {\sum\limits_{j = 1}^{k} {g\left( {\mu_{{i_{j} }}^{\diamondsuit } } \right)} } \right)} \right)} } \right)} \right)} \right) $$
  $$ B^{\diamondsuit } = h^{ - 1} \left( {\frac{1}{k}h\left( {g^{ - 1} \left( {\frac{1}{{C_{n}^{k} }}\sum\limits_{{1 \le i_{1} < ... < i_{k} \le n}} {g\left( {h^{ - 1} \left( {\sum\limits_{j = 1}^{k} {h\left( {\nu_{{i_{j} }}^{\diamondsuit } } \right)} } \right)} \right)} } \right)} \right)} \right) $$
3. when $\lambda_{1} = \lambda_{2} = ... = \lambda_{k} = \frac{1}{k}$, the GIVPQ-ROFMSM operator degrades into the IVPQ-ROFMSM operator, shown as follows:
  12 $$ \begin{gathered} IVPQ - ROFHM^{\left( k \right)} \left( {Q_{1} ,Q_{2} ,...,Q_{n} } \right) \hfill \\ = \frac{1}{{C_{n}^{k} }}\mathop \oplus \limits_{{1 \le i_{1} < ... < i_{k} \le n}} \left( {\mathop \otimes \limits_{j = 1}^{k} Q_{{i_{j} }} } \right)^{\frac{1}{k}} \hfill \\ = \left( {\left[ {A^{L} ,A^{U} } \right],\left[ {B^{L} ,B^{U} } \right]} \right) \hfill \\ \end{gathered} $$
  where
  $$ A^{\diamondsuit } = h^{ - 1} \left( {\frac{1}{{C_{n}^{k} }}\sum\limits_{{1 \le i_{1} < ... < i_{k} \le n}} {h\left( {g^{ - 1} \left( {\sum\limits_{j = 1}^{k} {\frac{1}{k}g\left( {\mu_{{i_{j} }}^{\diamondsuit } } \right)} } \right)} \right)} } \right) $$
  $$ B^{\diamondsuit } = g^{ - 1} \left( {\frac{1}{{C_{n}^{k} }}\sum\limits_{{1 \le i_{1} < ... < i_{k} \le n}} {g\left( {h^{ - 1} \left( {\sum\limits_{j = 1}^{k} {\frac{1}{k}h\left( {\nu_{{i_{j} }}^{\diamondsuit } } \right)} } \right)} \right)} } \right) $$

When taking different additive generators in Tables 1, 2, we can obtain such special cases:

1. If $g\left( x \right) = - \log x^{p}$ and $h\left( x \right) = - \log \left( {1 - x^{q} } \right)$, then GIVPQ-ROFMSM operator is converted into generalized interval valued p-q Rung orthopair fuzzy Algebraic Maclurin symmetric mean (GIVPQ-ROFAMSM) operator and is presented as follows:
  13 $$ GIVPQ - ROFAMSM^{{\left( {k,\lambda_{1} ,\lambda_{2} ,...,\lambda_{k} } \right)}} \left( {Q_{1} ,Q_{2} ,...,Q_{n} } \right) = \left( {\left[ {A^{L} ,A^{U} } \right],\left[ {B^{L} ,B^{U} } \right]} \right) $$
  where
  $$ A^{\diamondsuit } = \left( {\left( {1 - \left( {\prod\limits_{{1 \le i_{1} < ... < i_{k} \le n}} {\left( {1 - \left( {\prod\limits_{j = 1}^{k} {\left( {\mu_{{i_{j} }}^{\diamondsuit } } \right)^{{\lambda_{j} }} } } \right)^{p} } \right)^{{\frac{1}{{C_{n}^{k} }}}} } } \right)} \right)^{{\frac{1}{{\lambda_{1} + \lambda_{2} + ... + \lambda_{k} }}}} } \right)^{\frac{1}{p}} $$
  $$ B^{\diamondsuit } = \left( {1 - \left( {1 - \prod\limits_{{1 \le i_{1} < ... < i_{k} \le n}} {\left( {1 - \prod\limits_{j = 1}^{k} {\left( {1 - \left( {\nu_{{i_{j} }}^{\diamondsuit } } \right)^{q} } \right)^{{\lambda_{j} }} } } \right)^{{\frac{1}{{C_{n}^{k} }}}} } } \right)^{{\frac{1}{{\lambda_{1} + \lambda_{2} + ... + \lambda_{k} }}}} } \right)^{\frac{1}{q}} $$
2. If $g\left( x \right) = \log \left( {\frac{{2 - x^{p} }}{{x^{p} }}} \right)$ and $h\left( x \right) = \log \left( {\frac{{1 + x^{q} }}{{1 - x^{q} }}} \right)$, then GIVPQ-ROFMSM operator is converted into generalized interval valued p-q Rung orthopair fuzzy Einstein Maclurin symmetric mean (GIVPQ-ROFEMSM) operator and is presented as follows:
  14 $$ GIVPQ - ROFEMSM^{{\left( {k,\lambda_{1} ,\lambda_{2} ,...,\lambda_{k} } \right)}} \left( {Q_{1} ,Q_{2} ,...,Q_{n} } \right) = \left( {\left[ {A^{L} ,A^{U} } \right],\left[ {B^{L} ,B^{U} } \right]} \right) $$
  where
  $$ A^{\diamondsuit } = \left( {\frac{{2\left( {s^{\diamondsuit } - t^{\diamondsuit } } \right)^{{\frac{1}{{\lambda_{1} + \lambda_{2} + ... + \lambda_{k} }}}} }}{{\left( {s^{\diamondsuit } + 3t^{\diamondsuit } } \right)^{{\frac{1}{{\lambda_{1} + \lambda_{2} + ... + \lambda_{k} }}}} + \left( {s^{\diamondsuit } - t^{\diamondsuit } } \right)^{{\frac{1}{{\lambda_{1} + \lambda_{2} + ... + \lambda_{k} }}}} }}} \right)^{\frac{1}{p}} $$
  $$ B^{\diamondsuit } = \left( {\frac{{\left( {x^{\diamondsuit } + 3y^{\diamondsuit } } \right)^{{\frac{1}{{\lambda_{1} + \lambda_{2} + ... + \lambda_{k} }}}} - \left( {x^{\diamondsuit } - y^{\diamondsuit } } \right)^{{\frac{1}{{\lambda_{1} + \lambda_{2} + ... + \lambda_{k} }}}} }}{{\left( {x^{\diamondsuit } + 3y^{\diamondsuit } } \right)^{{\frac{1}{{\lambda_{1} + \lambda_{2} + ... + \lambda_{k} }}}} + \left( {x^{\diamondsuit } - y^{\diamondsuit } } \right)^{{\frac{1}{{\lambda_{1} + \lambda_{2} + ... + \lambda_{k} }}}} }}} \right)^{\frac{1}{q}} $$
  in which
  $$ s^{\diamondsuit } = \prod\limits_{{1 \le i_{1} < ... < i_{k} \le n}} {\left( {\prod\limits_{j = 1}^{k} {\left( {2 - \left( {\mu_{{i_{j} }}^{\diamondsuit } } \right)^{p} } \right)^{{\lambda_{j} }} } + 3\left( {\prod\limits_{j = 1}^{k} {\left( {\mu_{{i_{j} }}^{\diamondsuit } } \right)^{p} } } \right)^{{\lambda_{j} }} } \right)^{{\frac{1}{{C_{n}^{k} }}}} } $$
  $$ t^{\diamondsuit } = \prod\limits_{{1 \le i_{1} < ... < i_{k} \le n}} {\left( {\prod\limits_{j = 1}^{k} {\left( {2 - \left( {\mu_{{i_{j} }}^{\diamondsuit } } \right)^{p} } \right)^{{\lambda_{j} }} } - \left( {\prod\limits_{j = 1}^{k} {\left( {\mu_{{i_{j} }}^{\diamondsuit } } \right)^{p} } } \right)^{{\lambda_{j} }} } \right)^{{\frac{1}{{C_{n}^{k} }}}} } $$
  $$ x^{\diamondsuit } = \prod\limits_{{1 \le i_{1} < ... < i_{k} \le n}} {\left( {\prod\limits_{j = 1}^{k} {\left( {1 + \left( {\nu_{{i_{j} }}^{\diamondsuit } } \right)^{q} } \right)^{{\lambda_{j} }} } + 3\prod\limits_{j = 1}^{k} {\left( {1 - \left( {\nu_{{i_{j} }}^{\diamondsuit } } \right)^{q} } \right)^{{\lambda_{j} }} } } \right)^{{\frac{1}{{C_{n}^{k} }}}} } $$
  $$ y^{\diamondsuit } = \prod\limits_{{1 \le i_{1} < ... < i_{k} \le n}} {\left( {\prod\limits_{j = 1}^{k} {\left( {1 + \left( {\nu_{{i_{j} }}^{\diamondsuit } } \right)^{q} } \right)^{{\lambda_{j} }} } - \prod\limits_{j = 1}^{k} {\left( {1 - \left( {\nu_{{i_{j} }}^{\diamondsuit } } \right)^{q} } \right)^{{\lambda_{j} }} } } \right)^{{\frac{1}{{C_{n}^{k} }}}} } $$
3. If $g\left( x \right) = \log \left( {\frac{{\gamma + \left( {1 - \gamma } \right)x^{p} }}{{x^{p} }}} \right)$ and $h\left( x \right) = \log \left( {\frac{{\gamma + \left( {1 - \gamma } \right)\left( {1 - x^{q} } \right)}}{{1 - x^{q} }}} \right)$, then GIVPQ-ROFMSM operator is converted into generalized interval valued p-q Rung orthopair fuzzy Hamacher Maclurin symmetric mean (GIVPQ-QOFHMSM) operator and is presented as follows:
  15 $$ GIVPQ - ROFHMSM^{{\left( {k,\lambda_{1} ,\lambda_{2} ,...,\lambda_{k} } \right)}} \left( {Q_{1} ,Q_{2} ,...,Q_{n} } \right) = \left( {\left[ {A^{L} ,A^{U} } \right],\left[ {B^{L} ,B^{U} } \right]} \right) $$
  where
  $$ A^{\diamondsuit } = \left( {\frac{{\gamma \left( {s^{\diamondsuit } - t^{\diamondsuit } } \right)^{{\frac{1}{{\lambda_{1} + \lambda_{2} + ... + \lambda_{k} }}}} }}{{\left( {s^{\diamondsuit } + \left( {\gamma^{2} - 1} \right)t^{\diamondsuit } } \right)^{{\frac{1}{{\lambda_{1} + \lambda_{2} + ... + \lambda_{k} }}}} + \left( {\gamma - 1} \right)\left( {s^{\diamondsuit } - t^{\diamondsuit } } \right)^{{\frac{1}{{\lambda_{1} + \lambda_{2} + ... + \lambda_{k} }}}} }}} \right)^{\frac{1}{p}} $$
  $$ B^{\diamondsuit } = \left( {\frac{{\left( {x^{\diamondsuit } + \left( {\gamma^{2} - 1} \right)y^{\diamondsuit } } \right)^{{\frac{1}{{\lambda_{1} + \lambda_{2} + ... + \lambda_{k} }}}} - \left( {x^{\diamondsuit } - y^{\diamondsuit } } \right)^{{\frac{1}{{\lambda_{1} + \lambda_{2} + ... + \lambda_{k} }}}} }}{{\left( {x^{\diamondsuit } + \left( {\gamma^{2} - 1} \right)y^{\diamondsuit } } \right)^{{\frac{1}{{\lambda_{1} + \lambda_{2} + ... + \lambda_{k} }}}} + \left( {\gamma - 1} \right)\left( {x^{\diamondsuit } - y^{\diamondsuit } } \right)^{{\frac{1}{{\lambda_{1} + \lambda_{2} + ... + \lambda_{k} }}}} }}} \right)^{\frac{1}{q}} $$
  in which
  $$ s^{\diamondsuit } = \prod\limits_{{1 \le i_{1} < ... < i_{k} \le n}} {\left( {\prod\limits_{j = 1}^{k} {\left( {1 + \left( {\gamma - 1} \right)\left( {1 - \left( {\mu_{{i_{j} }}^{\diamondsuit } } \right)^{p} } \right)} \right)^{{\lambda_{j} }} } + \left( {\gamma^{2} - 1} \right)\left( {\prod\limits_{j = 1}^{k} {\left( {\mu_{{i_{j} }}^{\diamondsuit } } \right)^{p} } } \right)^{{\lambda_{j} }} } \right)^{{\frac{1}{{C_{n}^{k} }}}} } $$
  $$ t^{\diamondsuit } = \prod\limits_{{1 \le i_{1} < ... < i_{k} \le n}} {\left( {\prod\limits_{j = 1}^{k} {\left( {1 + \left( {\gamma - 1} \right)\left( {1 - \left( {\mu_{{i_{j} }}^{\diamondsuit } } \right)^{p} } \right)} \right)^{{\lambda_{j} }} } - \left( {\prod\limits_{j = 1}^{k} {\left( {\mu_{{i_{j} }}^{\diamondsuit } } \right)^{p} } } \right)^{{\lambda_{j} }} } \right)^{{\frac{1}{{C_{n}^{k} }}}} } $$
  $$ x^{\diamondsuit } = \prod\limits_{{1 \le i_{1} < ... < i_{k} \le n}} {\left( {\prod\limits_{j = 1}^{k} {\left( {1 + \left( {\gamma - 1} \right)\left( {\nu_{{i_{j} }}^{\diamondsuit } } \right)^{q} } \right)^{{\lambda_{j} }} } + \left( {\gamma^{2} - 1} \right)\prod\limits_{j = 1}^{k} {\left( {1 - \left( {\nu_{{i_{j} }}^{\diamondsuit } } \right)^{q} } \right)^{{\lambda_{j} }} } } \right)^{{\frac{1}{{C_{n}^{k} }}}} } $$
  $$ y^{\diamondsuit } = \prod\limits_{{1 \le i_{1} < ... < i_{k} \le n}} {\left( {\prod\limits_{j = 1}^{k} {\left( {1 + \left( {\gamma - 1} \right)\left( {\nu_{{i_{j} }}^{\diamondsuit } } \right)^{q} } \right)^{{\lambda_{j} }} } - \prod\limits_{j = 1}^{k} {\left( {1 - \left( {\nu_{{i_{j} }}^{\diamondsuit } } \right)^{q} } \right)^{{\lambda_{j} }} } } \right)^{{\frac{1}{{C_{n}^{k} }}}} } $$
4. If $g\left( x \right) = - \log \left( {\frac{\tau - 1}{{\tau^{{x^{p} }} - 1}}} \right)$ and $h\left( x \right) = - \log \left( {\frac{\tau - 1}{{\tau^{{1 - x^{q} }} - 1}}} \right)$, then GIVPQ-ROFMSM operator is converted into generalized interval valued p-q Rung orthopair fuzzy frank Maclurin symmetric mean (GIVPQ-ROFFMSM) operator and is presented as follows:
  16 $$ GIVPQ - ROFFMSM^{{\left( {k,\lambda_{1} ,\lambda_{2} ,...,\lambda_{k} } \right)}} \left( {Q_{1} ,Q_{2} ,...,Q_{n} } \right) = \left( {\left[ {A^{L} ,A^{U} } \right],\left[ {B^{L} ,B^{U} } \right]} \right) $$
  where
  $$ A^{\diamondsuit } = \left( {\log_{\tau } \left( {1 + \frac{{\left( {\tau^{{a^{\diamondsuit } }} - 1} \right)^{{\frac{1}{{\lambda_{1} + \lambda_{2} + ... + \lambda_{k} }}}} }}{{\left( {\tau - 1} \right)^{{\frac{1}{{\lambda_{1} + \lambda_{2} + ... + \lambda_{k} }} - 1}} }}} \right)} \right)^{\frac{1}{p}} $$
  $$ B^{\diamondsuit } = \left( {1 - \log_{\tau } \left( {1 + \frac{{\left( {\tau^{{1 - b^{\diamondsuit } }} - 1} \right)^{{\frac{1}{{\lambda_{1} + \lambda_{2} + ... + \lambda_{k} }}}} }}{{\left( {\tau - 1} \right)^{{\frac{1}{{\lambda_{1} + \lambda_{2} + ... + \lambda_{k} }} - 1}} }}} \right)} \right)^{\frac{1}{q}} $$
  in which
  $$ \begin{gathered} a^{\diamondsuit } = 1 - \log_{\tau } \left( {1 + \prod\limits_{{1 \le i_{1} < ... < i_{k} \le n}} {\left( {\tau^{{1 - \log_{\tau } \left( {1 + \frac{{\prod\limits_{j = 1}^{k} {\left( {\tau^{{\mu_{{i_{j} }}^{p} }} - 1} \right)^{{\lambda_{j} }} } }}{{\left( {\tau - 1} \right)^{{\sum\limits_{j = 1}^{k} {\lambda_{j} } - 1}} }}} \right)}} - 1} \right)^{{\frac{1}{{C_{n}^{k} }}}} } } \right) \hfill \\ \hfill \\ \end{gathered} $$
  $$ b^{\diamondsuit } = \log_{\tau } \left( {1 + \prod\limits_{{1 \le i_{1} < ... < i_{k} \le n}} {\left( {\tau^{{1 - \log_{\tau } \left( {1 + \frac{{\prod\limits_{j = 1}^{k} {\left( {\tau^{{1 - \left( {\nu_{{i_{j} }}^{\diamondsuit } } \right)^{q} }} - 1} \right)^{{\lambda_{j} }} } }}{{\left( {\tau - 1} \right)^{{\sum\limits_{j = 1}^{k} {\lambda_{j} } - 1}} }}} \right)}} - 1} \right)^{{\frac{1}{{C_{n}^{k} }}}} } } \right) $$

### GIVPQ-ROFWMSM operator based on ATN and ATCN

The GIVPQ-ROFMSM operator discussed in previous section only considers the interrelation between input arguments but ignore the importance of them. Nonetheless, the decision-making process will be heavily influenced by the importance of the input arguments. Hence, in this part, we introduce the GIVPQ-ROFWMSM operator as follows:

#### Definition 4.2

Let $Q_{i} = \left( {\left[ {\mu_{i}^{L} ,\mu_{i}^{U} } \right],\left[ {\nu_{i}^{L} ,\nu_{i}^{U} } \right]} \right)\left( {i = 1,2,...,n} \right)$ be IVPQ-ROFNs, $p,q \ge 1$, and let the weight vector of $Q_{i} = \left( {\left[ {\mu_{i}^{L} ,\mu_{i}^{U} } \right],\left[ {\nu_{i}^{L} ,\nu_{i}^{U} } \right]} \right)\left( {i = 1,2,...,n} \right)$ be $w = \left( {w_{1} ,w_{2} ,...,w_{n} } \right)$ which satisfying $\sum\limits_{i = 1}^{n} {w_{i} } = 1$. The GIVPQ-ROFWMSM operator of the IVPQ-ROFNs $Q_{i} \left( {i = 1,2,...,n} \right)$ can be defined as follows:

17 $$ \begin{gathered} GIVPQ - ROFWMSM^{{\left( {k,\lambda_{1} ,\lambda_{2} ,...,\lambda_{k} } \right)}} \left( {Q_{1} ,Q_{2} ,...,Q_{n} } \right) \hfill \\ = \left\{ \begin{gathered} \left( {\frac{1}{{C_{n - 1}^{k} }}\mathop \oplus \limits_{{1 \le i_{1} < ... < i_{k} \le n}} \left( {1 - \sum\limits_{j = 1}^{k} {w_{{i_{j} }} } } \right)\left( {\mathop \otimes \limits_{j = 1}^{k} Q_{{i_{j} }}^{{\lambda_{j} }} } \right)} \right)^{{\frac{1}{{\lambda_{1} + \lambda_{2} + ... + \lambda_{k} }}}} ,1 \le k < n \hfill \\ \left( {\mathop \otimes \limits_{i = 1}^{k} Q_{i}^{{\lambda_{i} + \frac{{1 - nw_{i} }}{n - 1}}} } \right)^{{\frac{1}{{\lambda_{1} + \lambda_{2} + ... + \lambda_{k} }}}} ,k = n \hfill \\ \end{gathered} \right. \hfill \\ \end{gathered} $$

where $\lambda_{1} ,\lambda_{2} ,...,\lambda_{k} \ge 0$, $k = 1,2,...,n$, $\left( {i_{1} ,i_{2} ,...,i_{k} } \right)$ traverses all the $k$ tuple combination of $\left( {1,2,...,n} \right)$, and $C_{n}^{k} = \frac{n!}{{k!\left( {n - k} \right)!}}$ is the binomial coefficient.

#### Theorem 4.2

Let $Q_{i} = \left( {\left[ {\mu_{i}^{L} ,\mu_{i}^{U} } \right],\left[ {\nu_{i}^{L} ,\nu_{i}^{U} } \right]} \right)\left( {i = 1,2,...,n} \right)$ be IVPQ-QOFNs,$\diamondsuit = \left\{ {U,L} \right\}$ $p,q \ge 1$. Then

18 $$ GIVPQ - ROFWMSM^{{\left( {k,\lambda_{1} ,\lambda_{2} ,...,\lambda_{k} } \right)}} \left( {Q_{1} ,Q_{2} ,...,Q_{n} } \right) = \left\{ \begin{gathered} \left( {\left[ {A^{L} ,A^{U} } \right],\left[ {B^{L} ,B^{U} } \right]} \right),1 \le k < n \hfill \\ \left( {\left[ {C^{L} ,C^{U} } \right],\left[ {D^{L} ,D^{U} } \right]} \right),k = n \hfill \\ \end{gathered} \right. $$

where

$$ A^{\diamondsuit } = g^{ - 1} \left( {\frac{1}{{\lambda_{1} + \lambda_{2} + ... + \lambda_{k} }}g\left( {h^{ - 1} \left( {\frac{1}{{C_{n - 1}^{k} }}\sum\limits_{{1 \le i_{1} < ... < i_{n} \le n}} {\left( {1 - \sum\limits_{j = 1}^{k} {w_{{i_{j} }} } } \right)h\left( {g^{ - 1} \left( {\sum\limits_{j = 1}^{k} {\lambda_{j} g\left( {\mu_{{i_{j} }}^{\diamondsuit } } \right)} } \right)} \right)} } \right)} \right)} \right) $$

$$ B^{\diamondsuit } = h^{ - 1} \left( {\frac{1}{{\lambda_{1} + \lambda_{2} + ... + \lambda_{k} }}h\left( {g^{ - 1} \left( {\frac{1}{{C_{n - 1}^{k} }}\sum\limits_{{1 \le i_{1} < ... < i_{n} \le n}} {\left( {1 - \sum\limits_{j = 1}^{k} {w_{{i_{j} }} } } \right)g\left( {h^{ - 1} \left( {\sum\limits_{j = 1}^{k} {\lambda_{j} h\left( {\nu_{{i_{j} }}^{\diamondsuit } } \right)} } \right)} \right)} } \right)} \right)} \right) $$

$$ C^{\diamondsuit } = g^{ - 1} \left( {\frac{1}{{\lambda_{1} + \lambda_{2} + ... + \lambda_{k} }}\sum\limits_{i = 1}^{k} {\left( {\lambda_{i} + \frac{{1 - nw_{i} }}{n - 1}} \right)g\left( {\mu_{i}^{\diamondsuit } } \right)} } \right) $$

$$ D^{\diamondsuit } = h^{ - 1} \left( {\frac{1}{{\lambda_{1} + \lambda_{2} + ... + \lambda_{k} }}\sum\limits_{i = 1}^{k} {\left( {\lambda_{i} + \frac{{1 - nw_{i} }}{n - 1}} \right)h\left( {\nu_{i}^{\diamondsuit } } \right)} } \right) $$

#### Proof

See Appendix C.

#### Property 4.2

Let $Q_{i} = \left( {\left[ {\mu_{i}^{L} ,\mu_{i}^{U} } \right],\left[ {\nu_{i}^{L} ,\nu_{i}^{U} } \right]} \right)\left( {i = 1,2,...,n} \right)$ and $Q_{i}^{*} = \left( {\left[ {\mu_{i}^{L*} ,\mu_{i}^{U*} } \right],\left[ {\nu_{i}^{L*} ,\nu_{i}^{U*} } \right]} \right)\left( {i = 1,2,...,n} \right)$ be IVPQ-ROFNs, $p,q \ge 1$. Then

1. Idempotency: If $Q_{i} = \left( {\left[ {\mu^{L} ,\mu^{U} } \right],\left[ {\nu^{L} ,\nu^{U} } \right]} \right)\left( {i = 1,2,...,n} \right)$ for all $i = 1,2,...,n$, then $GIVPQ - ROFWMSM^{{\left( {k,\lambda_{1} ,\lambda_{2} ,...,\lambda_{k} } \right)}} \left( {Q_{1} ,Q_{2} ,...,Q_{n} } \right) = \left( {\left[ {\mu^{L} ,\mu^{U} } \right],\left[ {\nu^{L} ,\nu^{U} } \right]} \right)$.
2. Monotonicity: If $\mu_{i}^{U} \le \mu_{i}^{U*}$, $\mu_{i}^{L} \le \mu_{i}^{L*}$, $\nu_{i}^{U} \ge \nu_{i}^{U*}$ and $\nu_{i}^{L} \ge \nu_{i}^{L*}$ for all $i = 1,2,...,n$, then
  $$ GIVPQ - ROFWMSM^{{\left( {k,\lambda_{1} ,\lambda_{2} ,...,\lambda_{k} } \right)}} \left( {Q_{1} ,Q_{2} ,...,Q_{n} } \right) \le GIVPQ - ROFWMSM^{{\left( {k,\lambda_{1} ,\lambda_{2} ,...,\lambda_{k} } \right)}} \left( {Q_{1}^{*} ,Q_{2}^{*} ,...,Q_{n}^{*} } \right) $$
3. Boundedness: $Q^{ - } \le GIVPQ - ROFWMSM^{{\left( {k,\lambda_{1} ,\lambda_{2} ,...,\lambda_{k} } \right)}} \left( {Q_{1} ,Q_{2} ,...,Q_{n} } \right) \le Q^{ + }$, where $Q^{ + } = \left( {\left[ {\mathop {\max }\limits_{i} \left\{ {\mu_{i}^{L} } \right\},\mathop {\max }\limits_{i} \left\{ {\mu_{i}^{U} } \right\}} \right],\left[ {\mathop {\min }\limits_{i} \left\{ {\nu_{i}^{L} } \right\},\mathop {\min }\limits_{i} \left\{ {\nu_{i}^{U} } \right\}} \right]} \right)$ and $Q^{ - } = \left( {\left[ {\mathop {\min }\limits_{i} \left\{ {\mu_{i}^{L} } \right\},\mathop {\min }\limits_{i} \left\{ {\mu_{i}^{U} } \right\}} \right],\left[ {\mathop {\max }\limits_{i} \left\{ {\nu_{i}^{L} } \right\},\mathop {\max }\limits_{i} \left\{ {\nu_{i}^{U} } \right\}} \right]} \right)$.

#### Proof

Similar to Property 4.1, so omitted here.

### Some special cases of GIVPQ-ROFWMSM operator

Likewise, we also obtain some special examples of the GIVPQ-ROFWMSM operator by setting different values for parameters and taking different additive generator in Tables 1, 2.

Here, we first introduce a special case of the GIVPQ-ROFWMSM operator by setting different values for parameters as follows:

If $w_{i} = \frac{1}{n}\left( {i = 1,2,...,n} \right)$, the established GIVPQ-ROFWMSM operator is simplified into the GIVPQ-ROFMSM operator as follows:

19 $$ \begin{gathered} IVPQ - ROFAGWMSM^{{\left( {k,\lambda_{1} ,\lambda_{2} ,...,\lambda_{k} } \right)}} \left( {Q_{1} ,Q_{2} ,...,Q_{n} } \right) \hfill \\ = \left\{ \begin{gathered} \left( {\frac{1}{{C_{n}^{k} }}\mathop \oplus \limits_{{1 \le i_{1} < ... < i_{k} \le n}} \left( {\mathop \otimes \limits_{j = 1}^{k} Q_{{i_{j} }}^{{\lambda_{j} }} } \right)} \right)^{{\frac{1}{{\lambda_{1} + \lambda_{2} + ... + \lambda_{k} }}}} ,1 \le k < n \hfill \\ \left( {\mathop \otimes \limits_{j = 1}^{k} Q_{{i_{j} }}^{{\lambda_{j} }} } \right)^{{\frac{1}{{\lambda_{1} + \lambda_{2} + ... + \lambda_{k} }}}} ,k = n \hfill \\ \end{gathered} \right. \hfill \\ = \left\{ \begin{gathered} \left( {\left[ {A^{L} ,A^{U} } \right],\left[ {B^{L} ,B^{U} } \right]} \right),1 \le k < n \hfill \\ \left( {\left[ {C^{L} ,C^{U} } \right],\left[ {D^{L} ,D^{U} } \right]} \right),k = n \hfill \\ \end{gathered} \right. \hfill \\ \end{gathered} $$

When $$1 \le k < n$$

$$ A^{\diamondsuit } = g^{ - 1} \left( {\frac{1}{{\lambda_{1} + \lambda_{2} + ... + \lambda_{k} }}g\left( {h^{ - 1} \left( {\frac{1}{{C_{n}^{k} }}\sum\limits_{{1 \le i_{1} < ... < i_{k} \le n}} {h\left( {g^{ - 1} \left( {\sum\limits_{j = 1}^{k} {\lambda_{j} g\left( {\mu_{{i_{j} }}^{\diamondsuit } } \right)} } \right)} \right)} } \right)} \right)} \right) $$

$$ B^{\diamondsuit } = h^{ - 1} \left( {\frac{1}{{\lambda_{1} + \lambda_{2} + ... + \lambda_{k} }}h\left( {g^{ - 1} \left( {\frac{1}{{C_{n}^{k} }}\sum\limits_{{1 \le i_{1} < ... < i_{k} \le n}} {g\left( {h^{ - 1} \left( {\sum\limits_{j = 1}^{k} {\lambda_{j} h\left( {\nu_{{i_{j} }}^{\diamondsuit } } \right)} } \right)} \right)} } \right)} \right)} \right) $$

When $$k = n$$

$$ C^{\diamondsuit } = g^{ - 1} \left( {\frac{1}{{\lambda_{1} + \lambda_{2} + ... + \lambda_{k} }}\sum\limits_{j = 1}^{k} {\lambda_{j} g\left( {\mu_{{i_{j} }}^{\diamondsuit } } \right)} } \right) $$

$$ D^{\diamondsuit } = h^{ - 1} \left( {\frac{1}{{\lambda_{1} + \lambda_{2} + ... + \lambda_{k} }}\sum\limits_{j = 1}^{k} {\lambda_{j} h\left( {\nu_{{i_{j} }}^{\diamondsuit } } \right)} } \right) $$

Some special cases of the GIVPQ-ROFWMSM operator when taking different additive generators in Tables 1, 2 are shown as follows:

1. If $g\left( x \right) = - \log x^{p}$ and $h\left( x \right) = - \log \left( {1 - x^{q} } \right)$, then GIVPQ-ROFWMSM operator is converted into generalized interval valued p-q Rung orthopair fuzzy Algebraic weighted Maclurin symmetric mean (GIVPQ-ROFAWMSM) operator and is presented as follows
  20 $$ GIVPQ - ROFAWMSM^{{\left( {k,\lambda_{1} ,\lambda_{2} ,...,\lambda_{k} } \right)}} \left( {Q_{1} ,Q_{2} ,...,Q_{n} } \right) = \left\{ \begin{gathered} \left( {\left[ {A^{L} ,A^{U} } \right],\left[ {B^{L} ,B^{U} } \right]} \right),1 \le k < n \hfill \\ \left( {\left[ {C^{L} ,C^{U} } \right],\left[ {D^{L} ,D^{U} } \right]} \right),k = n \hfill \\ \end{gathered} \right. $$
  1. When $$1 \le k < n$$
    $$ A^{\diamondsuit } = \left( {\left( {1 - \left( {\prod\limits_{{1 \le i_{1} < ... < i_{k} \le n}} {\left( {1 - \left( {\prod\limits_{j = 1}^{k} {\left( {\mu_{{i_{j} }}^{\diamondsuit } } \right)^{{\lambda_{j} }} } } \right)^{p} } \right)^{{1 - \sum\limits_{j = 1}^{k} {w_{{i_{j} }} } }} } } \right)^{{\frac{1}{{C_{n - 1}^{k} }}}} } \right)^{{\frac{1}{{\lambda_{1} + \lambda_{2} + ... + \lambda_{k} }}}} } \right)^{\frac{1}{p}} $$
    $$ B^{\diamondsuit } = \left( {1 - \left( {1 - \left( {\prod\limits_{{1 \le i_{1} < ... < i_{k} \le n}} {\left( {1 - \prod\limits_{j = 1}^{k} {\left( {1 - \left( {\nu_{{i_{j} }}^{\diamondsuit } } \right)^{q} } \right)^{{\lambda_{j} }} } } \right)^{{1 - \sum\limits_{j = 1}^{k} {w_{{i_{j} }} } }} } } \right)^{{\frac{1}{{C_{n - 1}^{k} }}}} } \right)^{{\frac{1}{{\lambda_{1} + \lambda_{2} + ... + \lambda_{k} }}}} } \right)^{\frac{1}{q}} $$
  2. When $$k = n$$
    $$ C^{\diamondsuit } = \left( {\prod\limits_{j = 1}^{k} {\left( {\mu_{j}^{\diamondsuit } } \right)^{{\lambda_{j} + \frac{{1 - nw_{j} }}{n - 1}}} } } \right)^{{\frac{1}{{\lambda_{1} + \lambda_{2} + ... + \lambda_{k} }}}} $$
    $$ D^{\diamondsuit } = \left( {1 - \left( {\prod\limits_{j = 1}^{k} {\left( {1 - \left( {\nu_{j}^{\diamondsuit } } \right)^{q} } \right)^{{\lambda_{j} + \frac{{1 - nw_{j} }}{n - 1}}} } } \right)^{{\frac{1}{{\lambda_{1} + \lambda_{2} + ... + \lambda_{k} }}}} } \right)^{\frac{1}{q}} $$
2. If $g\left( x \right) = \log \left( {\frac{{2 - x^{p} }}{{x^{p} }}} \right)$ and $h\left( x \right) = \log \left( {\frac{{1 + x^{q} }}{{1 - x^{q} }}} \right)$, then GIVPQ-ROFWMSM operator is converted into generalized interval valued p-q Rung orthopair fuzzy Einstein weighted Maclurin symmetric mean (GIVPQ-ROFEWMSM) operator and is presented as follows
  21 $$ GIVPQ - ROFEWMSM^{{\left( {k,\lambda_{1} ,\lambda_{2} ,...,\lambda_{k} } \right)}} \left( {Q_{1} ,Q_{2} ,...,Q_{n} } \right) = \left\{ \begin{gathered} \left( {\left[ {A^{L} ,A^{U} } \right],\left[ {B^{L} ,B^{U} } \right]} \right),1 \le k < n \hfill \\ \left( {\left[ {C^{L} ,C^{U} } \right],\left[ {D^{L} ,D^{U} } \right]} \right),k = n \hfill \\ \end{gathered} \right. $$
  1. When $$1 \le k < n$$
    $$ A^{\diamondsuit } = \left( {\frac{{2\left[ {\left( {s + 3t} \right)^{{\frac{1}{{C_{n - 1}^{k} }}}} - \left( {s - t} \right)^{{\frac{1}{{C_{n - 1}^{k} }}}} } \right]^{{\frac{1}{{\lambda_{1} + \lambda_{2} + ... + \lambda_{k} }}}} }}{{\left[ {\left( {s + 3t} \right)^{{\frac{1}{{C_{n - 1}^{k} }}}} + 3\left( {s - t} \right)^{{\frac{1}{{C_{n - 1}^{k} }}}} } \right]^{{\frac{1}{{\lambda_{1} + \lambda_{2} + ... + \lambda_{k} }}}} + \left[ {\left( {s + 3t} \right)^{{\frac{1}{{C_{n - 1}^{k} }}}} - \left( {s - t} \right)^{{\frac{1}{{C_{n - 1}^{k} }}}} } \right]^{{\frac{1}{{\lambda_{1} + \lambda_{2} + ... + \lambda_{k} }}}} }}} \right)^{\frac{1}{p}} $$
    $$ B^{\diamondsuit } = \left( {\frac{{\left( {x^{{\frac{1}{{C_{n - 1}^{k} }}}} + 3y^{{\frac{1}{{C_{n - 1}^{k} }}}} } \right)^{{\frac{1}{{\lambda_{1} + \lambda_{2} + ... + \lambda_{k} }}}} - \left( {x^{{\frac{1}{{C_{n - 1}^{k} }}}} - y^{{\frac{1}{{C_{n - 1}^{k} }}}} } \right)^{{\frac{1}{{\lambda_{1} + \lambda_{2} + ... + \lambda_{k} }}}} }}{{\left( {x^{{\frac{1}{{C_{n - 1}^{k} }}}} + 3y^{{\frac{1}{{C_{n - 1}^{k} }}}} } \right)^{{\frac{1}{{\lambda_{1} + \lambda_{2} + ... + \lambda_{k} }}}} + \left( {x^{{\frac{1}{{C_{n - 1}^{k} }}}} - y^{{\frac{1}{{C_{n - 1}^{k} }}}} } \right)^{{\frac{1}{{\lambda_{1} + \lambda_{2} + ... + \lambda_{k} }}}} }}} \right)^{\frac{1}{q}} $$
    In which
    $$ s^{\diamondsuit } = \prod\limits_{{1 \le i_{1} < ... < i_{k} \le n}} {\left( {\prod\limits_{j = 1}^{k} {\left( {1 + \left( {1 - \left( {\mu_{{i_{j} }}^{\diamondsuit } } \right)^{p} } \right)} \right)^{{\lambda_{j} }} } + 3\left( {\prod\limits_{j = 1}^{k} {\left( {\mu_{{i_{j} }}^{\diamondsuit } } \right)^{{\lambda_{j} }} } } \right)^{p} } \right)^{{1 - \sum\limits_{j = 1}^{k} {w_{j} } }} } $$
    $$ t^{\diamondsuit } = \prod\limits_{{1 \le i_{1} < ... < i_{k} \le n}} {\left( {\prod\limits_{j = 1}^{k} {\left( {1 + \left( {1 - \left( {\mu_{{i_{j} }}^{\diamondsuit } } \right)^{p} } \right)} \right)^{{\lambda_{j} }} } - \left( {\prod\limits_{j = 1}^{k} {\left( {\mu_{{i_{j} }}^{\diamondsuit } } \right)^{{\lambda_{j} }} } } \right)^{p} } \right)^{{1 - \sum\limits_{j = 1}^{k} {w_{j} } }} } $$
    $$ x^{\diamondsuit } = \prod\limits_{{1 \le i_{1} < ... < i_{k} \le n}} {\left( {\prod\limits_{j = 1}^{k} {\left( {1 + \left( {\nu_{{i_{j} }}^{\diamondsuit } } \right)^{q} } \right)^{{\lambda_{j} }} } + 3\prod\limits_{j = 1}^{k} {\left( {1 - \left( {\nu_{{i_{j} }}^{\diamondsuit } } \right)^{q} } \right)^{{\lambda_{j} }} } } \right)^{{1 - \sum\limits_{j = 1}^{k} {w_{j} } }} } , $$
    $$ y^{\diamondsuit } = \prod\limits_{{1 \le i_{1} < ... < i_{k} \le n}} {\left( {\prod\limits_{j = 1}^{k} {\left( {1 + \left( {\nu_{{i_{j} }}^{\diamondsuit } } \right)^{q} } \right)^{{\lambda_{j} }} } - \prod\limits_{j = 1}^{k} {\left( {1 - \left( {\nu_{{i_{j} }}^{\diamondsuit } } \right)^{q} } \right)^{{\lambda_{j} }} } } \right)^{{1 - \sum\limits_{j = 1}^{k} {w_{j} } }} } . $$
  2. When $$k = n$$
    $$ C^{\diamondsuit } = \left( {\frac{{2\left( {\prod\limits_{j = 1}^{k} {\left( {\left( {\mu_{j}^{\diamondsuit } } \right)^{p} } \right)^{{\lambda_{j} + \frac{{1 - nw_{j} }}{n - 1}}} } } \right)^{{\frac{1}{{\lambda_{1} + \lambda_{2} + ... + \lambda_{k} }}}} }}{{\left( {\prod\limits_{j = 1}^{k} {\left( {1 + \left( {\gamma - 1} \right)\left( {1 - \left( {\mu_{j}^{\diamondsuit } } \right)^{p} } \right)^{{\lambda_{j} + \frac{{1 - nw_{j} }}{n - 1}}} } \right)} } \right)^{{\frac{1}{{\lambda_{1} + \lambda_{2} + ... + \lambda_{k} }}}} + \left( {\prod\limits_{j = 1}^{k} {\left( {\left( {\mu_{j}^{\diamondsuit } } \right)^{p} } \right)^{{\lambda_{j} + \frac{{1 - nw_{j} }}{n - 1}}} } } \right)^{{\frac{1}{{\lambda_{1} + \lambda_{2} + ... + \lambda_{k} }}}} }}} \right)^{\frac{1}{p}} $$
    $$ D^{\diamondsuit } = \left( {\frac{{\left( {\prod\limits_{j = 1}^{k} {\left( {1 + \left( {\nu_{j}^{\diamondsuit } } \right)^{q} } \right)^{{\lambda_{j} + \frac{{1 - nw_{j} }}{n - 1}}} } } \right)^{{\frac{1}{{\lambda_{1} + \lambda_{2} + ... + \lambda_{k} }}}} - \left( {\prod\limits_{j = 1}^{k} {\left( {1 - \left( {\nu_{j}^{\diamondsuit } } \right)^{q} } \right)^{{\lambda_{j} + \frac{{1 - nw_{j} }}{n - 1}}} } } \right)^{{\frac{1}{{\lambda_{1} + \lambda_{2} + ... + \lambda_{k} }}}} }}{{\left( {\prod\limits_{j = 1}^{k} {\left( {1 + \left( {\nu_{j}^{\diamondsuit } } \right)^{q} } \right)^{{\lambda_{j} + \frac{{1 - nw_{j} }}{n - 1}}} } } \right)^{{\frac{1}{{\lambda_{1} + \lambda_{2} + ... + \lambda_{k} }}}} + \left( {\prod\limits_{j = 1}^{k} {\left( {1 - \left( {\nu_{j}^{\diamondsuit } } \right)^{q} } \right)^{{\lambda_{j} + \frac{{1 - nw_{j} }}{n - 1}}} } } \right)^{{\frac{1}{{\lambda_{1} + \lambda_{2} + ... + \lambda_{k} }}}} }}} \right)^{\frac{1}{q}} $$
3. If $g\left( x \right) = \log \left( {\frac{{\gamma + \left( {1 - \gamma } \right)x^{p} }}{{x^{p} }}} \right)$ and $h\left( x \right) = \log \left( {\frac{{\gamma + \left( {1 - \gamma } \right)\left( {1 - x^{q} } \right)}}{{1 - x^{q} }}} \right)$, then GIVPQ-ROFWMSM operator is converted into generalized interval valued p-q Rung orthopair fuzzy Hamacher weighted Maclurin symmetric mean (GIVPQ-ROFHWMSM) operator and is presented as follows:
  22 $$ GIVPQ - ROFHWMSM^{{\left( {k,\lambda_{1} ,\lambda_{2} ,...,\lambda_{k} } \right)}} \left( {Q_{1} ,Q_{2} ,...,Q_{n} } \right) = \left\{ \begin{gathered} \left( {\left[ {A^{L} ,A^{U} } \right],\left[ {B^{L} ,B^{U} } \right]} \right),1 \le k < n \hfill \\ \left( {\left[ {C^{L} ,C^{U} } \right],\left[ {D^{L} ,D^{U} } \right]} \right),k = n \hfill \\ \end{gathered} \right. $$
  1. When $$1 \le k < n$$
    $$ A^{\diamondsuit } = \left( {\frac{{\gamma \left[ {\left( {s + \left( {\gamma^{2} - 1} \right)t} \right)^{{\frac{1}{{C_{n - 1}^{k} }}}} - \left( {s - t} \right)^{{\frac{1}{{C_{n - 1}^{k} }}}} } \right]^{{\frac{1}{{\lambda_{1} + \lambda_{2} + ... + \lambda_{k} }}}} }}{{\left[ {\left( {s + \left( {\gamma^{2} - 1} \right)t} \right)^{{\frac{1}{{C_{n - 1}^{k} }}}} + \left( {\gamma^{2} - 1} \right)\left( {s - t} \right)^{{\frac{1}{{C_{n - 1}^{k} }}}} } \right]^{{\frac{1}{{\lambda_{1} + \lambda_{2} + ... + \lambda_{k} }}}} + \left( {\gamma - 1} \right)\left[ {\left( {s + \left( {\gamma^{2} - 1} \right)t} \right)^{{\frac{1}{{C_{n - 1}^{k} }}}} - \left( {s - t} \right)^{{\frac{1}{{C_{n - 1}^{k} }}}} } \right]^{{\frac{1}{{\lambda_{1} + \lambda_{2} + ... + \lambda_{k} }}}} }}} \right)^{\frac{1}{p}} $$
    $$ B^{\diamondsuit } = \left( {\frac{{\left( {x^{{\frac{1}{{C_{n - 1}^{k} }}}} + \left( {\gamma^{2} - 1} \right)y^{{\frac{1}{{C_{n - 1}^{k} }}}} } \right)^{{\frac{1}{{\lambda_{1} + \lambda_{2} + ... + \lambda_{k} }}}} - \left( {x^{{\frac{1}{{C_{n - 1}^{k} }}}} - y^{{\frac{1}{{C_{n - 1}^{k} }}}} } \right)^{{\frac{1}{{\lambda_{1} + \lambda_{2} + ... + \lambda_{k} }}}} }}{{\left( {x^{{\frac{1}{{C_{n - 1}^{k} }}}} + \left( {\gamma^{2} - 1} \right)y^{{\frac{1}{{C_{n - 1}^{k} }}}} } \right)^{{\frac{1}{{\lambda_{1} + \lambda_{2} + ... + \lambda_{k} }}}} + \left( {\gamma - 1} \right)\left( {x^{{\frac{1}{{C_{n - 1}^{k} }}}} - y^{{\frac{1}{{C_{n - 1}^{k} }}}} } \right)^{{\frac{1}{{\lambda_{1} + \lambda_{2} + ... + \lambda_{k} }}}} }}} \right)^{\frac{1}{q}} $$
    In which
    $$ s^{\diamondsuit } = \prod\limits_{{1 \le i_{1} < ... < i_{k} \le n}} {\left( {\prod\limits_{j = 1}^{k} {\left( {1 + \left( {\gamma - 1} \right)\left( {1 - \left( {\mu_{{i_{j} }}^{\diamondsuit } } \right)^{p} } \right)} \right)^{{\lambda_{j} }} } + \left( {\gamma^{2} - 1} \right)\left( {\prod\limits_{j = 1}^{k} {\left( {\mu_{{i_{j} }}^{\diamondsuit } } \right)^{{\lambda_{j} }} } } \right)^{p} } \right)^{{1 - \sum\limits_{j = 1}^{k} {w_{j} } }} } $$
    $$ t^{\diamondsuit } = \prod\limits_{{1 \le i_{1} < ... < i_{k} \le n}} {\left( {\prod\limits_{j = 1}^{k} {\left( {1 + \left( {\gamma - 1} \right)\left( {1 - \left( {\mu_{{i_{j} }}^{\diamondsuit } } \right)^{p} } \right)} \right)^{{\lambda_{j} }} } - \left( {\prod\limits_{j = 1}^{k} {\left( {\mu_{{i_{j} }}^{\diamondsuit } } \right)^{{\lambda_{j} }} } } \right)^{p} } \right)^{{1 - \sum\limits_{j = 1}^{k} {w_{j} } }} } $$
    $$ x^{\diamondsuit } = \prod\limits_{{1 \le i_{1} < ... < i_{k} \le n}} {\left( {\prod\limits_{j = 1}^{k} {\left( {1 + \left( {\gamma - 1} \right)\left( {\nu_{{i_{j} }}^{\diamondsuit } } \right)^{q} } \right)^{{\lambda_{j} }} } + \left( {\gamma^{2} - 1} \right)\prod\limits_{j = 1}^{k} {\left( {1 - \left( {\nu_{{i_{j} }}^{\diamondsuit } } \right)^{q} } \right)^{{\lambda_{j} }} } } \right)^{{1 - \sum\limits_{j = 1}^{k} {w_{j} } }} } , $$
    $$ y^{\diamondsuit } = \prod\limits_{{1 \le i_{1} < ... < i_{k} \le n}} {\left( {\prod\limits_{j = 1}^{k} {\left( {1 + \left( {\gamma - 1} \right)\left( {\nu_{{i_{j} }}^{\diamondsuit } } \right)^{q} } \right)^{{\lambda_{j} }} } - \prod\limits_{j = 1}^{k} {\left( {1 - \left( {\nu_{{i_{j} }}^{\diamondsuit } } \right)^{q} } \right)^{{\lambda_{j} }} } } \right)^{{1 - \sum\limits_{j = 1}^{k} {w_{j} } }} } . $$
  2. When $$k = n$$
    $$ C^{\diamondsuit } = \left( {\frac{{\gamma \left( {\prod\limits_{j = 1}^{k} {\left( {\left( {\mu_{j}^{\diamondsuit } } \right)^{p} } \right)^{{\lambda_{j} + \frac{{1 - nw_{j} }}{n - 1}}} } } \right)^{{\frac{1}{{\lambda_{1} + \lambda_{2} + ... + \lambda_{k} }}}} }}{{\left( {\prod\limits_{j = 1}^{k} {\left( {1 + \left( {\gamma - 1} \right)\left( {1 - \left( {\mu_{j}^{\diamondsuit } } \right)^{p} } \right)^{{\lambda_{j} + \frac{{1 - nw_{j} }}{n - 1}}} } \right)} } \right)^{{\frac{1}{{\lambda_{1} + \lambda_{2} + ... + \lambda_{k} }}}} + \left( {\gamma - 1} \right)\left( {\prod\limits_{j = 1}^{k} {\left( {\left( {\mu_{j}^{\diamondsuit } } \right)^{p} } \right)^{{\lambda_{j} + \frac{{1 - nw_{j} }}{n - 1}}} } } \right)^{{\frac{1}{{\lambda_{1} + \lambda_{2} + ... + \lambda_{k} }}}} }}} \right)^{\frac{1}{p}} $$
    $$ D^{\diamondsuit } = \left( {\frac{{\left( {\prod\limits_{j = 1}^{k} {\left( {1 + \left( {\gamma - 1} \right)\left( {\nu_{j}^{\diamondsuit } } \right)^{q} } \right)^{{\lambda_{j} + \frac{{1 - nw_{j} }}{n - 1}}} } } \right)^{{\frac{1}{{\lambda_{1} + \lambda_{2} + ... + \lambda_{k} }}}} - \left( {\prod\limits_{j = 1}^{k} {\left( {1 - \left( {\nu_{j}^{\diamondsuit } } \right)^{q} } \right)^{{\lambda_{j} + \frac{{1 - nw_{j} }}{n - 1}}} } } \right)^{{\frac{1}{{\lambda_{1} + \lambda_{2} + ... + \lambda_{k} }}}} }}{{\left( {\prod\limits_{j = 1}^{k} {\left( {1 + \left( {\gamma - 1} \right)\left( {\nu_{j}^{\diamondsuit } } \right)^{q} } \right)^{{\lambda_{j} + \frac{{1 - nw_{j} }}{n - 1}}} } } \right)^{{\frac{1}{{\lambda_{1} + \lambda_{2} + ... + \lambda_{k} }}}} + \left( {\gamma - 1} \right)\left( {\prod\limits_{j = 1}^{k} {\left( {1 - \left( {\nu_{j}^{\diamondsuit } } \right)^{q} } \right)^{{\lambda_{j} + \frac{{1 - nw_{j} }}{n - 1}}} } } \right)^{{\frac{1}{{\lambda_{1} + \lambda_{2} + ... + \lambda_{k} }}}} }}} \right)^{\frac{1}{q}} $$
4. If $g\left( x \right) = - \log \left( {\frac{\tau - 1}{{\tau^{{x^{p} }} - 1}}} \right)$ and $h\left( x \right) = - \log \left( {\frac{\tau - 1}{{\tau^{{1 - x^{q} }} - 1}}} \right)$, then GIVPQ-ROFWMSM operator is converted into generalized interval valued p-q Rung orthopair fuzzy frank weighted Maclurin symmetric mean (GIVPQ-ROFFWMSM) operator and is presented as follows:
  23 $$ GIVPQ - ROFFWMSM^{{\left( {k,\lambda_{1} ,\lambda_{2} ,...,\lambda_{k} } \right)}} \left( {Q_{1} ,Q_{2} ,...,Q_{n} } \right) = \left\{ \begin{gathered} \left( {\left[ {A^{L} ,A^{U} } \right],\left[ {B^{L} ,B^{U} } \right]} \right),1 \le k < n \hfill \\ \left( {\left[ {C^{L} ,C^{U} } \right],\left[ {D^{L} ,D^{U} } \right]} \right),k = n \hfill \\ \end{gathered} \right. $$
  1. When $$1 \le k < n$$
    $$ A^{\diamondsuit } = \left( {\log_{\tau } \left( {1 + \frac{{\left( {\tau^{{a^{\diamondsuit } }} - 1} \right)^{{\frac{1}{{\lambda_{1} + \lambda_{2} + ... + \lambda_{k} }}}} }}{{\left( {\tau - 1} \right)^{{\frac{1}{{\lambda_{1} + \lambda_{2} + ... + \lambda_{k} }} - 1}} }}} \right)} \right)^{\frac{1}{p}} $$
    $$ B^{\diamondsuit } = \left( {1 - \log_{\tau } \left( {1 + \frac{{\left( {\tau^{{1 - b^{\diamondsuit } }} - 1} \right)^{{\frac{1}{{\lambda_{1} + \lambda_{2} + ... + \lambda_{k} }}}} }}{{\left( {\tau - 1} \right)^{{\frac{1}{{\lambda_{1} + \lambda_{2} + ... + \lambda_{k} }} - 1}} }}} \right)} \right)^{\frac{1}{q}} $$
    In which
    $$ \begin{gathered} a^{\diamondsuit } = 1 - \log_{\tau } \left( {1 + \left( {\prod\limits_{{1 \le i_{1} < ... < i_{k} \le n}} {\left( {\tau^{{1 - a_{i} }} - 1} \right)^{{1 - \sum\limits_{j = 1}^{k} {w_{{i_{j} }} } }} } } \right)^{{\frac{1}{{C_{n - 1}^{k} }}}} } \right) \hfill \\ b^{\diamondsuit } = \log_{\tau } \left( {1 + \left( {\prod\limits_{{1 \le i_{1} < ... < i_{k} \le n}} {\left( {\tau^{{b_{i}^{\diamondsuit } }} - 1} \right)^{{1 - \sum\limits_{j = 1}^{k} {w_{{i_{j} }} } }} } } \right)^{{\frac{1}{{C_{n - 1}^{k} }}}} } \right) \hfill \\ \end{gathered} $$
    $$ a_{i}^{\diamondsuit } = \log \tau \left( {1 + \frac{{\prod\limits_{j = 1}^{k} {\left( {\tau^{{\left( {\mu_{{i_{j} }}^{\diamondsuit } } \right)^{p} }} - 1} \right)^{{\lambda_{j} }} } }}{{\left( {\tau - 1} \right)^{{\sum\limits_{j = 1}^{k} {\lambda_{j} } - 1}} }}} \right) $$
    $$ b_{i}^{\diamondsuit } = 1 - \log \tau \left( {1 + \frac{{\prod\limits_{j = 1}^{k} {\left( {\tau^{{1 - \left( {\nu_{{i_{j} }}^{\diamondsuit } } \right)^{q} }} - 1} \right)^{{\lambda_{j} }} } }}{{\left( {\tau - 1} \right)^{{\sum\limits_{j = 1}^{k} {\lambda_{j} } - 1}} }}} \right) $$
  2. When $$k = n$$
    $$ C^{\diamondsuit } = \left( {\log_{\tau } \left( {1 + \left( {\prod\limits_{j = 1}^{k} {\left( {\tau^{{\left( {\mu_{j}^{\diamondsuit } } \right)^{p} }} - 1} \right)^{{\lambda_{j} + \frac{{1 - nw_{j} }}{n - 1}}} } } \right)^{{\frac{1}{{\lambda_{1} + \lambda_{2} + ... + \lambda_{k} }}}} } \right)} \right)^{\frac{1}{p}} $$
    $$ D^{\diamondsuit } = \left( {1 - \log_{\tau } \left( {1 + \left( {\prod\limits_{j = 1}^{k} {\left( {\tau^{{1 - \left( {\nu_{j}^{\diamondsuit } } \right)^{q} }} - 1} \right)^{{\lambda_{j} + \frac{{1 - nw_{j} }}{n - 1}}} } } \right)^{{\frac{1}{{\lambda_{1} + \lambda_{2} + ... + \lambda_{k} }}}} } \right)} \right)^{\frac{1}{q}} $$

## The PID and NID based on the projection measure of IVPQ-ROFNs

In this section, first, we define the module and cosine measure of IVPQ-ROFNs. Second, we propose the projection measure of IVPQ-ROFNs based on the module and cosine measure. Finally, based on the projection measure of IVPQ-ROFNs, the PID and the NID is introduced. We also study some valuable properties of them.

### The projection measure of IVPQ-ROFNs

To introduce the projection measure of IVPQ-ROFNs, we need to define the module and cosine measure of IVPQ-ROFNs. In the following, we first give the definition of module of IVPQ-ROFNs.

#### Definition 5.1

Let $Q = \left( {\left[ {\mu^{L} ,\mu^{U} } \right],\left[ {\nu^{L} ,\nu^{U} } \right]} \right)$ be an IVPQ-ROFNs, the module of $Q$ can be defined as follows:

24 $$ \left| Q \right| = \left( {\left( {\mu^{L} } \right)^{2p} + \left( {\mu^{U} } \right)^{2p} + \left( {\nu^{L} } \right)^{2q} + \left( {\nu^{U} } \right)^{2q} + \left( {\pi^{L} } \right)^{2r} + \left( {\pi^{U} } \right)^{2r} } \right)^{\frac{1}{2}} $$

#### Definition 5.2

Let $Q_{i} = \left( {\left[ {\mu_{i}^{L} ,\mu_{i}^{U} } \right],\left[ {\nu_{i}^{L} ,\nu_{i}^{U} } \right]} \right)\left( {i = 1,2} \right)$ be any two IVPQ-ROFNs, the inner product of the $Q_{1}$ and $Q_{2}$ can be defined as follows:

$$ Q_{1} \cdot Q_{2} = \left( {\mu_{1}^{L} \mu_{2}^{L} } \right)^{2p} + \left( {\mu_{1}^{U} \mu_{2}^{U} } \right)^{2p} + \left( {\nu_{1}^{L} \nu_{2}^{L} } \right)^{2q} + \left( {\nu_{1}^{U} \nu_{2}^{U} } \right)^{2q} + \left( {\pi_{1}^{L} \pi_{2}^{L} } \right)^{2r} + \left( {\pi_{1}^{U} \pi_{2}^{U} } \right)^{2r} $$

#### Definition 5.3

Let $Q_{i} = \left( {\left[ {\mu_{i}^{L} ,\mu_{i}^{U} } \right],\left[ {\nu_{i}^{L} ,\nu_{i}^{U} } \right]} \right)\left( {i = 1,2} \right)$ be any two IVPQ-ROFNs, the cosine of the angle between $Q_{1}$ and $Q_{2}$ can be defined as follows:

25 $$ \cos \left( {Q_{1} ,Q_{2} } \right) = \frac{{Q_{1} \cdot Q_{2} }}{{\left| {Q_{1} } \right|\left| {Q_{2} } \right|}} $$

#### Theorem 5.1

Let $Q_{i} = \left( {\left[ {\mu_{i}^{L} ,\mu_{i}^{U} } \right],\left[ {\nu_{i}^{L} ,\nu_{i}^{U} } \right]} \right)\left( {i = 1,2,...,n} \right)$ be a collection of IVPQ-ROFNs, where $Q^{ + } = \left( {\left[ {\mathop {\max }\limits_{i} \left\{ {\mu_{i}^{L} } \right\},\mathop {\max }\limits_{i} \left\{ {\mu_{i}^{U} } \right\}} \right],\left[ {\mathop {\min }\limits_{i} \left\{ {\nu_{i}^{L} } \right\},\mathop {\min }\limits_{i} \left\{ {\nu_{i}^{U} } \right\}} \right]} \right)$ is the positive ideal point (PIP) and $Q^{ - } = \left( {\left[ {\mathop {\min }\limits_{i} \left\{ {\mu_{i}^{L} } \right\},\mathop {\min }\limits_{i} \left\{ {\mu_{i}^{U} } \right\}} \right],\left[ {\mathop {\max }\limits_{i} \left\{ {\nu_{i}^{L} } \right\},\mathop {\max }\limits_{i} \left\{ {\nu_{i}^{U} } \right\}} \right]} \right)$ is the negative ideal point (NIP). Let $\cos \left( {Q_{i} ,Q^{ + } } \right)$ and $\cos \left( {Q_{i} ,Q^{ - } } \right)$ denote the cosines of the included angle between $Q_{1}$ and $Q^{ + }$ and between $Q_{i}$ and $Q^{ - }$, respectively, and they satisfy the following properties:

1. $$\cos \left( {Q_{i} ,Q^{ + } } \right) = \cos \left( {Q^{ + } ,Q_{i} } \right)$$ and $$\cos \left( {Q_{i} ,Q^{ - } } \right) = \cos \left( {Q^{ - } ,Q_{i} } \right)$$;
2. $$0 \le \cos \left( {Q_{i} ,Q^{ + } } \right) \le 1$$ and $$0 \le \cos \left( {Q_{i} ,Q^{ - } } \right) \le 1$$;
3. $\cos \left( {Q_{i} ,Q^{ + } } \right) = 1$ if and only if $Q_{i} = Q^{ + }$;
4. $\cos \left( {Q_{i} ,Q^{ - } } \right) = 1$ if and only if $Q_{i} = Q^{ - }$.

#### Proof

Similar to the Appendix A in literature^65^, so omitted here.

#### Definition 5.4

Let $Q_{i} = \left( {\left[ {\mu_{i}^{L} ,\mu_{i}^{U} } \right],\left[ {\nu_{i}^{L} ,\nu_{i}^{U} } \right]} \right)\left( {i = 1,2} \right)$ be any two IVPQ-ROFNs, the projection of $Q_{1}$ on $Q_{2}$ can be defined as follows:

26 $$ {\text{Proj}}_{{Q_{2} }} \left( {Q_{1} } \right) = \left| {Q_{1} } \right|\cos \left( {Q_{1} ,Q_{2} } \right) = \frac{{Q_{1} \cdot Q_{2} }}{{\left| {Q_{2} } \right|}} $$

Based on the definitions given above, we can obtain some valuable theorems.

#### Theorem 5.2

Let $Q_{i} = \left( {\left[ {\mu_{i}^{L} ,\mu_{i}^{U} } \right],\left[ {\nu_{i}^{L} ,\nu_{i}^{U} } \right]} \right)\left( {i = 1,2,...,n} \right)$ be a collection of IVPQ-ROFNs, where $Q^{ + } = \left( {\left[ {\mathop {\max }\limits_{i} \left\{ {\mu_{i}^{L} } \right\},\mathop {\max }\limits_{i} \left\{ {\mu_{i}^{U} } \right\}} \right],\left[ {\mathop {\min }\limits_{i} \left\{ {\nu_{i}^{L} } \right\},\mathop {\min }\limits_{i} \left\{ {\nu_{i}^{U} } \right\}} \right]} \right)$ is the PIP and $Q^{ - } = \left( {\left[ {\mathop {\min }\limits_{i} \left\{ {\mu_{i}^{L} } \right\},\mathop {\min }\limits_{i} \left\{ {\mu_{i}^{U} } \right\}} \right],\left[ {\mathop {\max }\limits_{i} \left\{ {\nu_{i}^{L} } \right\},\mathop {\max }\limits_{i} \left\{ {\nu_{i}^{U} } \right\}} \right]} \right)$ is the NIP. The projections ${\text{Proj}}_{{Q^{ + } }} \left( {Q_{i} } \right)$, ${\text{Proj}}_{{Q^{ - } }} \left( {Q_{i} } \right)$, ${\text{Proj}}_{{Q_{i} }} \left( {Q^{ + } } \right)$ and ${\text{Proj}}_{{Q_{i} }} \left( {Q^{ - } } \right)$ satisfy the following properties:

1. $$0 \le {\text{Proj}}_{{Q^{ + } }} \left( {Q_{i} } \right) \le \left| {Q_{i} } \right|$$, $$0 \le {\text{Proj}}_{{Q^{ - } }} \left( {Q_{i} } \right) \le \left| {Q_{i} } \right|$$, $$0 \le {\text{Proj}}_{{Q_{i} }} \left( {Q^{ + } } \right) \le \left| {Q^{ + } } \right|$$ and $$0 \le {\text{Proj}}_{{Q_{i} }} \left( {Q^{ - } } \right) \le \left| {Q^{ - } } \right|$$;
2. $${{{\text{Proj}}_{{Q^{ + } }} \left( {Q_{i} } \right)} \mathord{\left/ {\vphantom {{{\text{Proj}}_{{Q^{ + } }} \left( {Q_{i} } \right)} {{\text{Proj}}_{{Q_{i} }} \left( {Q^{ + } } \right)}}} \right. \kern-0pt} {{\text{Proj}}_{{Q_{i} }} \left( {Q^{ + } } \right)}} = {{\left| {Q_{i} } \right|} \mathord{\left/ {\vphantom {{\left| {Q_{i} } \right|} {\left| {Q^{ + } } \right|}}} \right. \kern-0pt} {\left| {Q^{ + } } \right|}}$$ and $${{{\text{Proj}}_{{Q^{ - } }} \left( {Q_{i} } \right)} \mathord{\left/ {\vphantom {{{\text{Proj}}_{{Q^{ - } }} \left( {Q_{i} } \right)} {{\text{Proj}}_{{Q_{i} }} \left( {Q^{ - } } \right)}}} \right. \kern-0pt} {{\text{Proj}}_{{Q_{i} }} \left( {Q^{ - } } \right)}} = {{\left| {Q_{i} } \right|} \mathord{\left/ {\vphantom {{\left| {Q_{i} } \right|} {\left| {Q^{ - } } \right|}}} \right. \kern-0pt} {\left| {Q^{ - } } \right|}}$$;
3. $${\text{Proj}}_{{Q^{ + } }} \left( {Q_{i} } \right) = {\text{Proj}}_{{Q_{i} }} \left( {Q^{ + } } \right) = \left| {Q_{i} } \right|$$ if $$Q_{i} = Q^{ + }$$;
4. $${\text{Proj}}_{{Q^{ - } }} \left( {Q_{i} } \right) = {\text{Proj}}_{{Q_{i} }} \left( {Q^{ - } } \right) = \left| {Q_{i} } \right|$$ if $$Q_{i} = Q^{ - }$$.

#### Proof

Similar to the Appendix B in literature^65^, so omitted here.

Compared with the distance measure of IVPQ-ROFN in literature^28^. The projection methods reflect closeness degree, which consider both angle and distance. At the same time, by comparing with the PIP and the NIP, it reflects people's psychological state, which is more consistent with the real decision-making situation We can judge whether the $Q_{1}$ is closer to $Q^{ + }$ or $Q^{ - }$ based on the value of the projections between them. The great value of ${\text{Proj}}_{{Q^{ + } }} \left( {Q_{i} } \right)$, the closer $Q_{i}$ to the positive ideal point $Q^{ + }$. In contrast, the smaller value of ${\text{Proj}}_{{Q^{ + } }} \left( {Q_{i} } \right)$, the farther $Q_{i}$ from the positive ideal point $Q^{ + }$.

### The PID and NID based on the projection of IVPQ-ROFNs

Previous section, we introduce the projection of IVPQ-ROFNs to measure the closeness degree between two IVPQ-ROFNs. However, any $Q_{i}$ has the highest ${\text{Proj}}_{{Q^{ + } }} \left( {Q_{i} } \right)$ value is not guaranteed have the lowest ${\text{Proj}}_{{Q^{ - } }} \left( {Q_{i} } \right)$ valued, and vice versa. Further, in real decision making environment, we not only need to know how closer the $Q_{i}$ to $Q^{ + }$, but also $Q_{i}$ to $Q^{ - }$, and even consider both comprehensively. Hence, based on the projection measure of IVPQ-ROFNs, we further define the PID and NID of IVPQ-ROFNs.

#### Definition 5.5

Let $Q_{i} = \left( {\left[ {\mu_{i}^{L} ,\mu_{i}^{U} } \right],\left[ {\nu_{i}^{L} ,\nu_{i}^{U} } \right]} \right)\left( {i = 1,2,...,n} \right)$ be a collection of IVPQ-ROFNs, where $Q^{ + } = \left( {\left[ {\mathop {\max }\limits_{i} \left\{ {\mu_{i}^{L} } \right\},\mathop {\max }\limits_{i} \left\{ {\mu_{i}^{U} } \right\}} \right],\left[ {\mathop {\min }\limits_{i} \left\{ {\nu_{i}^{L} } \right\},\mathop {\min }\limits_{i} \left\{ {\nu_{i}^{U} } \right\}} \right]} \right)$ is the PIP and $Q^{ - } = \left( {\left[ {\mathop {\min }\limits_{i} \left\{ {\mu_{i}^{L} } \right\},\mathop {\min }\limits_{i} \left\{ {\mu_{i}^{U} } \right\}} \right],\left[ {\mathop {\max }\limits_{i} \left\{ {\nu_{i}^{L} } \right\},\mathop {\max }\limits_{i} \left\{ {\nu_{i}^{U} } \right\}} \right]} \right)$ is the NIP. Then the PID and NID can be defined as follows:

27 $$ P_{i}^{ + } = \frac{{{\text{Proj}}_{{Q^{ + } }} \left( {Q_{i} } \right)}}{{{\text{Proj}}_{{Q^{ + } }} \left( {Q_{i} } \right) + {\text{Proj}}_{{Q^{ - } }} \left( {Q_{i} } \right)}} $$

28 $$ P_{i}^{ - } = \frac{{{\text{Proj}}_{{Q^{ - } }} \left( {Q_{i} } \right)}}{{{\text{Proj}}_{{Q^{ + } }} \left( {Q_{i} } \right) + {\text{Proj}}_{{Q^{ - } }} \left( {Q_{i} } \right)}} $$

#### Theorem 5.3

The PID and NID defined in Definition 5.5 satisfy the following properties:

1. $$P_{i}^{ + } + P_{i}^{ - } = 1$$;
2. $$0 \le P_{i}^{ + } \le 1$$ and $$0 \le P_{i}^{ - } \le 1$$;
3. $P_{i}^{ + }$ and $P_{i}^{ - }$ can not be both 0 or 1 at same time;
4. $P_{i}^{ + } = 1$ if and only if ${\text{Proj}}_{{Q^{ + } }} \left( {Q_{i} } \right) > 0$ and ${\text{Proj}}_{{Q^{ - } }} \left( {Q_{i} } \right) = 0$, $P_{i}^{ + } = 0$ if and only if ${\text{Proj}}_{{Q^{ + } }} \left( {Q_{i} } \right) = 0$ and ${\text{Proj}}_{{Q^{ - } }} \left( {Q_{i} } \right) > 0$;
5. $P_{i}^{ - } = 1$ if and only if ${\text{Proj}}_{{Q^{ + } }} \left( {Q_{i} } \right) = 0$ and ${\text{Proj}}_{{Q^{ - } }} \left( {Q_{i} } \right) > 0$, $P_{i}^{ + } = 0$ if and only if ${\text{Proj}}_{{Q^{ + } }} \left( {Q_{i} } \right) = 0$ and ${\text{Proj}}_{{Q^{ - } }} \left( {Q_{i} } \right) > 0$.

#### Proof

Similar to the Appendix C in literature^65^, so omitted here.

The PID and NID reflect closeness degree, which not only consider both angle and distance but also with two reference points. At the same time, by comparing with the PIP and the NIP, it reflects people's psychological state, which is more consistent with the real decision-making situation We can judge whether the $Q_{i}$ is closer to $Q^{ + }$ or $Q^{ - }$ based on the values of PID and NID. The larger the value of $P_{i}^{ + }$, the closer is $Q_{i}$ to $Q^{ + }$. Conversely, the smaller the value of $P_{i}^{ + }$,the farther $Q_{i}$ is from $Q^{ + }$. Similarly, the larger the value of $P_{i}^{ - }$, the closer is $Q_{i}$ to $Q^{ - }$.The smaller the value of $P_{i}^{ - }$, the farther $Q_{i}$ is from $Q^{ - }$.

## The created approaches to IVPQ-ROF MAGDM problems with regret theory

In this section, we propose a modified regret theory for MAGDM with IVPQ-ROF information. Firstly, the IVPQ-ROF MAGDM problem is illustrated. In view of this, we utilize the GIVPQ-ROFWMSM operator to aggregate the evaluation information under each attribute into a comprehensive opinion. Then, we design a modified regret theory for solving the IVPQ-ROF MAGDM problem. Finally, we present the decision-making steps of the proposed method.

### Problem description

Let $O = \left\{ {o_{1} ,o_{2} ,...,o_{m} } \right\}$ be a set of alternatives, $C = \left\{ {c_{1} ,c_{2} ,...,c_{n} } \right\}$ be a set of attributes, and $D = \left\{ {d_{1} ,d_{2} ,...,d_{T} } \right\}$ be a set of DMs. $W = \left\{ {w_{1} ,w_{2} ,...,w_{n} } \right\}$ is the weight vector of attributes, which satisfies $0 \le w_{j} \le 1$ and $\sum\limits_{j = 1}^{n} {w_{j} } = 1$. $\delta = \left( {\delta_{1} ,\delta_{2} ,...,\delta_{T} } \right)$ is a weight vector of DMs, which satisfies $0 \le \delta_{t} \le 1$ and $\sum\limits_{t = 1}^{T} {\delta_{t} } = 1$. According to the IVPQ-ROFMAGDM problem, the decision matrix ${\mathbb{Q}}_{t} = \left[ {Q_{ij}^{t} } \right]_{m \times n}$ given by DM $d_{t}$ can be defined as follows:

29 $$ {\mathbb{Q}}_{t} = \left[ {\begin{array}{*{20}c} {Q_{11}^{t} } & {Q_{12}^{t} } & \cdots & {Q_{1n}^{t} } \\ {Q_{21}^{t} } & {Q_{22}^{t} } & \cdots & {Q_{2n}^{t} } \\ \vdots & \vdots & \ddots & \vdots \\ {Q_{m1}^{t} } & {Q_{m2}^{t} } & \cdots & {Q_{mn}^{t} } \\ \end{array} } \right] $$

where $Q_{ij}^{t}$ denotes the evaluation value of alternative $o_{i}$ under attribute $c_{j}$ given by DM $d_{t}$ $\left( {i = 1,2,...,m;j = 1,2,...,n;t = 1,2,...,T} \right)$.

### The IVPQ-ROFMAGDM method with modified regret theory

As we stated in “Introduction” section, traditional regret-rejoice function only compares with the best alternative, but ignore the worst alternative when calculating the regret-rejoice value. However, DMs make decisions based not only on the best one, but the worst one. Hence, we introduce a novel IVPQ-ROFMAGDM method with modified regret-rejoice function which considers both best one and worst one. The main steps are organized as follows:

Step 1.: Standardize the individual decision matrices.
  As there usually are two types of attributes, namely, benefit type and cost type. Hence, we need to normalize the decision matrices under each attribute by utilizing Eq. (30) before aggregating.
  30 $$ Q_{ij}^{t} = \left\{ \begin{gathered} Q_{ij}^{t} = \left( {\left[ {\mu_{ij}^{Lt} ,\mu_{ij}^{Ut} } \right],\left[ {\nu_{ij}^{Lt} ,\nu_{ij}^{Ut} } \right]} \right),{\text{for benefit type}} \hfill \\ \left( {Q_{ij}^{t} } \right)^{c} = \left( {\left[ {\nu_{ij}^{Lt} ,\nu_{ij}^{Ut} } \right],\left[ {\mu_{ij}^{Lt} ,\mu_{ij}^{Ut} } \right]} \right),{\text{for cost type}} \hfill \\ \end{gathered} \right. $$
  where $\left( {Q_{ij}^{t} } \right)^{c}$ is the complement of $Q_{ij}^{t}$.
Step 2.: Aggregating the individual decision metrics ${\mathbb{Q}}_{t}$ under different attribute based on Eq. (31).
  31 $$ Q_{ij} = \left( {\left[ {\left( {1 - \prod\limits_{t = 1}^{T} {\left( {1 - \left( {\mu_{ij}^{Lt} } \right)^{p} } \right)^{{\delta_{t} }} } } \right)^{\frac{1}{p}} ,\left( {1 - \prod\limits_{t = 1}^{T} {\left( {1 - \left( {\mu_{ij}^{Ut} } \right)^{p} } \right)^{{\delta_{t} }} } } \right)^{\frac{1}{p}} } \right],\left[ {\prod\limits_{t = 1}^{T} {\left( {\nu_{ij}^{Lt} } \right)^{{\delta_{t} }} } ,\prod\limits_{t = 1}^{T} {\left( {\nu_{ij}^{Ut} } \right)^{{\delta_{t} }} } } \right]} \right) $$
  Then, the comprehensive decision matrix ${\mathbb{Q}}$ can be built as follows:
  32 $$ {\mathbb{Q}}_{t} = \left[ {\begin{array}{*{20}c} {Q_{11} } & {Q_{12} } & \cdots & {Q_{1n} } \\ {Q_{21} } & {Q_{22} } & \cdots & {Q_{2n} } \\ \vdots & \vdots & \ddots & \vdots \\ {Q_{m1} } & {Q_{m2} } & \cdots & {Q_{mn} } \\ \end{array} } \right] $$
  where $Q_{ij}$ represents the integrated evaluating value of alternative $o_{i}$ under attribute $c_{j}$.
Step 3.: Integrating evaluation values of alternative $o_{i}$ under all attributes. The integrated value $Q_{i}$ are computed according to the GIVPQ-ROFWMSM operator as follows:
  33 $$ \begin{gathered} GIVPQ - ROFWMSM^{{\left( {k,\lambda_{1} ,\lambda_{2} ,...,\lambda_{k} } \right)}} \left( {Q_{i1} ,Q_{i2} ,...,Q_{in} } \right) \hfill \\ = \left\{ \begin{gathered} \left( {\frac{1}{{C_{n - 1}^{r} }}\mathop \oplus \limits_{{1 \le i_{1} < ... < i_{r} \le n}} \left( {1 - \sum\limits_{j = 1}^{k} {w_{{i_{j} }} } } \right)\left( {\mathop \otimes \limits_{j = 1}^{k} Q_{{i_{j} }}^{{\lambda_{j} }} } \right)} \right)^{{\frac{1}{{\lambda_{1} + \lambda_{2} + ... + \lambda_{k} }}}} ,1 \le k < n \hfill \\ \left( {\mathop \otimes \limits_{i = 1}^{k} Q_{i}^{{\lambda_{i} + \frac{{1 - nw_{i} }}{n - 1}}} } \right)^{{\frac{1}{{\lambda_{1} + \lambda_{2} + ... + \lambda_{k} }}}} ,k = n \hfill \\ \end{gathered} \right. \hfill \\ \end{gathered} $$
Step 4.: Calculating the projection of $Q_{i}$ on $Q^{ + }$ and $Q_{i}$ on $Q^{ - }$ according to Eqs. (34), (35).
  34 $$ {\text{Proj}}_{{Q^{ + } }} \left( {Q_{i} } \right) = \left| {Q_{i} } \right|\cos \left( {Q_{i} ,Q^{ + } } \right) = \frac{{Q_{i} \cdot Q^{ + } }}{{\left| {Q^{ + } } \right|}} $$
  35 $$ {\text{Proj}}_{{Q^{ - } }} \left( {Q_{i} } \right) = \left| {Q_{i} } \right|\cos \left( {Q_{i} ,Q^{ - } } \right) = \frac{{Q_{i} \cdot Q^{ - } }}{{\left| {Q^{ - } } \right|}} $$
  where $Q^{ + } = \left( {\left[ {\mathop {\max }\limits_{i} \left\{ {\mu_{i}^{L} } \right\},\mathop {\max }\limits_{i} \left\{ {\mu_{i}^{U} } \right\}} \right],\left[ {\mathop {\min }\limits_{i} \left\{ {\nu_{i}^{L} } \right\},\mathop {\min }\limits_{i} \left\{ {\nu_{i}^{U} } \right\}} \right]} \right)$ is the PIP and $Q^{ - } = \left( {\left[ {\mathop {\min }\limits_{i} \left\{ {\mu_{i}^{L} } \right\},\mathop {\min }\limits_{i} \left\{ {\mu_{i}^{U} } \right\}} \right],\left[ {\mathop {\max }\limits_{i} \left\{ {\nu_{i}^{L} } \right\},\mathop {\max }\limits_{i} \left\{ {\nu_{i}^{U} } \right\}} \right]} \right)$ is the NIP.
Step 5.: Calculating the PID and NID of alternative $o_{i}$ based on Eqs. (36), (37).
  36 $$ P_{i}^{ + } { = }\frac{{{\text{Proj}}_{{Q^{ + } }} \left( {Q_{i} } \right)}}{{{\text{Proj}}_{{Q^{ + } }} \left( {Q_{i} } \right) + {\text{Proj}}_{{Q^{ - } }} \left( {Q_{i} } \right)}} $$
  37 $$ P_{i}^{ - } { = }\frac{{{\text{Proj}}_{{Q^{ - } }} \left( {Q_{i} } \right)}}{{{\text{Proj}}_{{Q^{ + } }} \left( {Q_{i} } \right) + {\text{Proj}}_{{Q^{ - } }} \left( {Q_{i} } \right)}} $$
Step 6.: Calculate the regret -rejoice value of alternative $o_{i}$ according to Eqs. (38)–(40).
  38 $$ REG\left( {o_{i} } \right) = \frac{{1 - \exp \left\{ { - \beta \left( {u_{i} - u^{ + } } \right)} \right\} + 1 - \exp \left\{ { - \beta \left( {v^{ + } - v_{i} } \right)} \right\}}}{2} $$
  39 $$ REJ\left( {o_{i} } \right) = = \frac{{1 - \exp \left\{ { - \beta \left( {u_{i} - u^{ - } } \right)} \right\} + 1 - \exp \left\{ { - \beta \left( {v^{ - } - v_{i} } \right)} \right\}}}{2} $$
  40 $$ {\mathbb{R}}\left( {o_{i} } \right) = REG\left( {o_{i} } \right) + REJ\left( {o_{i} } \right) $$
  where $$u_{i} = \left( {P_{i}^{ + } } \right)^{\alpha }$$,$$v_{i} = \left( {P_{i}^{ - } } \right)^{\alpha }$$,$$u^{ + } = \mathop {\max }\limits_{i} \left\{ {\left( {P_{i}^{ + } } \right)^{\alpha } } \right\}$$,$$v^{ + } = \mathop {\min }\limits_{i} \left\{ {\left( {P_{i}^{ - } } \right)^{\alpha } } \right\}$$,$$u^{ - } = \mathop {\min }\limits_{i} \left\{ {\left( {P_{i}^{ + } } \right)^{\alpha } } \right\}$$,$$v^{ - } = \mathop {\max }\limits_{i} \left\{ {\left( {P_{i}^{ - } } \right)^{\alpha } } \right\}$$.
Step 7.: Determining the ranking result of all alternative according to the regret -rejoice value ${\mathbb{R}}\left( {o_{i} } \right)$. The ${\mathbb{R}}\left( {o_{i} } \right)$ of the best alternative should be the maximum, conversely, the ${\mathbb{R}}\left( {o_{i} } \right)$ of the worst alternative should be the minimum.

In this subsection, we introduce novel regret function and rejoice function. Their counterparts in “Regret theory” section only consider the PIP when measuring regret and rejoice values, while ignoring the NIP. The functions proposed in this section consider both the PIP and NIP, which is more comprehensive. For the PIP $Q^{ + }$, $u^{ + }$ is the positive utility value, the smaller the $u_{i} - u^{ + }$, the farther away $u_{i}$ is from $u^{ + }$. On the contrary, $u^{ - }$ is the negative utility value, the bigger the $u_{i} - u^{ - }$, the farther away $u_{i}$ is from $u^{ - }$. For the NIP $Q^{ - }$, $v^{ + }$ is the positive utility value, the smaller the $v^{ + } - v_{i}$, the farther away $v_{i}$ is from $v^{ + }$. Conversely, the larger the $v^{ - } - v_{i}$, the farther away $v_{i}$ is to $v^{ - }$. For the regret function, the regret aversion is unlikely to occur when both $u_{i} - u^{ + }$ and $v^{ + } - v_{i}$ are relatively large. For the rejoice function, the is more likely to happen when both $u_{i} - u^{ - }$ and $v^{ - } - v_{i}$ are relatively large.

### The specific decision procedures for the IVPQ-ROF MAGDM method

**Figure 3:**
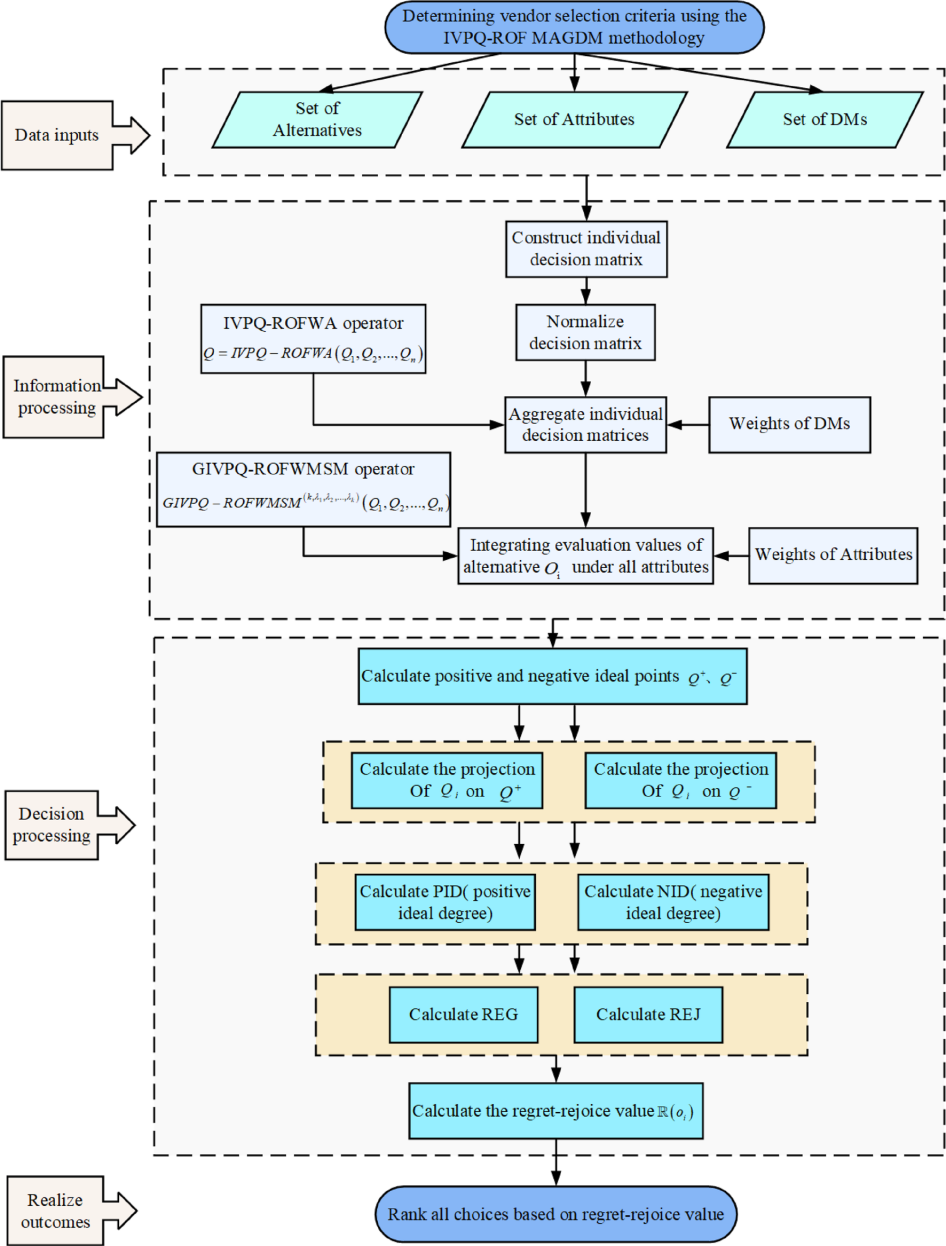
The flowchart of the proposed method.

Based on the above analysis, the specific decision procedures for the IVPQ-ROF MAGDM method are comprehensively reviewed, and we also provide the flowchart in Fig. 3.

Step 1.: Constructing the individual decision matric ${\mathbb{Q}}_{t} = \left[ {Q_{ij}^{t} } \right]_{m \times n}$.
Step 2.: Normalizing and standardize the individual decision matrices.
Step 3.: Aggregating the individual decision matric based on Eq. (31).
Step 4.: Aggregating the evaluation value of alternative $o_{i}$ under all attributes based on GIVPQ-ROFWMSM operator.
Step 5.: Calculating the projection of $Q_{i}$ on $Q^{ + }$ and $Q_{i}$ on $Q^{ - }$ based on Eqs. (34), (35).
Step 6.: Computing the PID and NID of alternative $o_{i}$ according to Eqs. (36), (37).
Step 7.: Calculating the regret-rejoice values of alternative $o_{i}$ based on Eqs. (38)–(40).
Step 8.: Ranking all alternatives according to their regret-rejoice values. The higher the regret-rejoice value, the higher the ranking.

## Case study

At present, Chinese manufacturing enterprises are facing the following enormous transformation and upgrading pressure:

1. It is urgent to establish differentiated competitive advantages due to backward or excess production capacity, fierce homogeneous competition, rapidly rising labor costs and rapid growth of customers' personalized demands.
2. The increase of migrant workers returning to their hometowns to start businesses, the change of the new generation of migrant workers' employment concept, the lack of senior technicians, recruitment difficulties, enterprises urgently need to reduce staff and improve efficiency.
3. Emerging information technology, rapidly changing market conditions, strategic factors of production supply and demand contradiction intensifies, enterprises are facing challenges of digital transformation.

Due to the great initiative and support of the Party and the country, especially the excellent performance of intelligent factories in the prevention and control of COVID-19 epidemic and the resumption of work and production, the manufacturing industry has accelerated the pace of the construction of intelligent factories. Because the construction of intelligent factory needs a lot of equipment and building materials, so the selection of excellent suppliers is of great significance for the construction of intelligent factory.

Suppose an enterprise is planning to build a green factory. The enterprise intends to purchase a lot of equipment and building materials, and there are five sustainable suppliers that can supply them. The five supplier alternatives are denoted by $O = \left\{ {o_{1} ,o_{2} ,o_{3} ,o_{4} ,o_{5} } \right\}$. Four attributes of each supplier are considered in SSS process: $c_{1}$: Green capability; $c_{2}$: Management skill; $c_{3}$:Price; $c_{4}$: Geographical position. To select an appropriate supplier, the enterprise invites three experts denoted by $d = \left\{ {d_{1} ,d_{2} ,d_{3} } \right\}$ to evaluate the five suppliers. The IVPQ-ROFNs are utilized to evaluate the alternative $o_{i} \in O$ with respect to attribute $c_{j} \in C$. The weight vectors of DMs and attributes are $\delta = \left( {0.25,0.35,0.4} \right)$ and $w = \left( {0.21,0.26,0.33,0.2} \right)$.

### Application of the proposed method

To solve the SSS IVPQ-ROF MAGDM problem, we employ the proposed method in Sect. 6. Due to this method involves many important parameters, it is necessary to set the parameters in advance. For parameter $p$ and $q$, we should set them based on the IVPQ-ROFNs gives by DMs. For example, if $\left( {\left[ {0.5,0.9} \right],\left[ {0.6,0.7} \right]} \right)$ is given as an evaluation value, it is not an IVPQ-ROFN when $\max \left\{ {p,q} \right\} \le 3$. But it is an IVPQ-ROFN when $\min \left\{ {p,q} \right\} \ge 4$. Hence, we determine $p = q = 4$ in our methodology. For parameters $\alpha$ and $\beta$, in current studies, they usually determined based on the DMs’ subjective judgement^57,59,60^, so we set $\alpha = 0.88$ and $\beta = 0.3$ in this section. For parameter $k$, as the involvement of 4 attributes in SSS issues above, to reflect the correlation between pairwise indicators, we choose $k = 2$. When there are many attributes, we can select a larger $k$ to reduce the computational burden. When $k$ is selected, the parameters $\lambda_{1}$ and $\lambda_{2}$ can be determined, they also usually determined according to the DMs’ subjective preference^48,49^. Hence, we choose $\lambda_{1} = \lambda_{2} = 1$ for convenience. The values of these parameters are compiled in Table 5. After determine the parameters, the main steps of our method can be presented as follows:

**Table 5:** The values of the parameters involved in this method.

Parameter	Value	Parameter	Value
$p$	4	$\gamma$	1.5
$q$	4	$\alpha$	0.88
$k$	2	$\beta$	0.3
$\lambda_{1}$	1	$\lambda_{2}$	1

Step 1.: Constructing the individual decision matrices. The original decision matrices are shown in Tables 6, 7, 8.

**Table 6:** The original individual decision matrix given by DM $d_{1}$.

	$c_{1}$	$c_{2}$	$c_{3}$	$c_{4}$
$o_{1}$	([0.4,0.4], [0.5,0.6])	([0.3,0.4], [0.3,0.6])	([0.2,0.3], [0.4,0.6])	([0.4,0.6], [0.2,0.3])
$o_{2}$	([0.7,0.8], [0.1,0.2])	([0.6,0.7], [0.1,0.2])	([0.5,0.6], [0.5,0.7])	([0.6,0.8], [0.1,0.2])
$o_{3}$	([0.6,0.7], [0.1,0.2])	([0.7,0.8], [0.1,0.1])	([0.1,0.2], [0.5,0.6])	([0.5,0.9], [0.1,0.1])
$o_{4}$	([0.3,0.4], [0.2,0.2])	([0.5,0.6], [0.2,0.3])	([0.1,0.2], [0.4,0.5])	([0.3,0.7], [0.1,0.2])
$o_{5}$	([0.2,0.5], [0.3,0.4])	([0.2,0.6], [0.2,0.3])	([0.1,0.3], [0.3,0.5])	([0.2,0.8], [0.1,0.2])

**Table 7:** The original individual decision matrix given by DM $d_{2}$.

	$c_{1}$	$c_{2}$	$c_{3}$	$c_{4}$
$o_{1}$	([0.6,0.7], [0.5,0.7])	([0.5,0.6], [0.2,0.5])	([0.5,0.6], [0.5,0.6])	([0.4,0.6], [0.5,0.6])
$o_{2}$	([0.8,0.9], [0.1,0.2])	([0.7,0.9], [0.1,0.3])	([0.5,0.7], [0.5,0.8])	([0.5,0.8], [0.2,0.3])
$o_{3}$	([0.9,0.9], [0.6,0.7])	([0.7,0.8], [0.1,0.3])	([0.2,0.3], [0.3,0.9])	([0.6,0.7], [0.1,0.2])
$o_{4}$	([0.4,0.5], [0.1,0.2])	([0.7,0.7], [0.3,0.4])	([0.3,0.4], [0.5,0.6])	([0.5,0.5], [0.3,0.3])
$o_{5}$	([0.2,0.6], [0.3,0.3])	([0.6,0.8], [0.2,0.5])	([0.5,0.6], [0.2,0.8])	([0.6,0.6], [0.4,0.5])

**Table 8:** The original individual decision matrix given by DM $d_{3}$.

	$c_{1}$	$c_{2}$	$c_{3}$	$c_{4}$
$o_{1}$	([0.2,0.5], [0.6,0.7])	([0.3,0.6], [0.4,0.5])	([0.5,0.5], [0.4,0.4])	([0.4,0.7], [0.3,0.6])
$o_{2}$	([0.8,0.8], [0.1,0.1])	([0.6,0.8], [0.1,0.2])	([0.4,0.6], [0.6,0.8])	([0.5,0.9], [0.3,0.4])
$o_{3}$	([0.6,0.9], [0.4,0.5])	([0.7,0.7], [0.2,0.3])	([0.4,0.6], [0.1,0.8])	([0.1,0.8], [0.1,0.2])
$o_{4}$	([0.5,0.5], [0.3,0.4])	([0.5,0.7], [0.4,0.4])	([0.1,0.4], [0.6,0.8])	([0.3,0.8], [0.2,0.3])
$o_{5}$	([0.4,0.7], [0.6,0.6])	([0.5,0.8], [0.5,0.5])	([0.2,0.3], [0.1,0.9])	([0.5,0.6], [0.1,0.1])
Step 2.: Normalizing the individual decision matrices. As the attribute $c_{3}$ is cost attribute, so we only need to normalize the evaluation value under the attribute $c_{3}$. The normalized results are shown in Tables 9, 10, 11.

**Table 9:** The normalized individual decision matrix given by DM $d_{1}$.

	$c_{1}$	$c_{2}$	$c_{3}$	$c_{4}$
$o_{1}$	([0.4,0.4], [0.5,0.6])	([0.3,0.4], [0.3,0.6])	([0.4,0.6], [0.2,0.3])	([0.4,0.6], [0.2,0.3])
$o_{2}$	([0.7,0.8], [0.1,0.2])	([0.6,0.7], [0.1,0.2])	([0.5,0.7], [0.5,0.6])	([0.6,0.8], [0.1,0.2])
$o_{3}$	([0.6,0.7], [0.1,0.2])	([0.7,0.8], [0.1,0.1])	([0.5,0.6], [0.1,0.2])	([0.5,0.9], [0.1,0.1])
$o_{4}$	([0.3,0.4], [0.2,0.2])	([0.5,0.6], [0.2,0.3])	([0.4,0.5], [0.1,0.2])	([0.3,0.7], [0.1,0.2])
$o_{5}$	([0.2,0.5], [0.3,0.4])	([0.2,0.6], [0.2,0.3])	([0.3,0.5], [0.1,0.3])	([0.2,0.8], [0.1,0.2])

**Table 10:** The normalized individual decision matrix given by DM $d_{2}$.

	$c_{1}$	$c_{2}$	$c_{3}$	$c_{4}$
$o_{1}$	([0.6,0.7], [0.5,0.7])	([0.5,0.6], [0.2,0.5])	([0.5,0.6], [0.5,0.6])	([0.4,0.6], [0.5,0.6])
$o_{2}$	([0.8,0.9], [0.1,0.2])	([0.7,0.9], [0.1,0.3])	([0.5,0.8], [0.5,0.7])	([0.5,0.8], [0.2,0.3])
$o_{3}$	([0.9,0.9], [0.6,0.7])	([0.7,0.8], [0.1,0.3])	([0.3,0.9], [0.2,0.3])	([0.6,0.7], [0.1,0.2])
$o_{4}$	([0.4,0.5], [0.1,0.2])	([0.7,0.7], [0.3,0.4])	([0.5,0.6], [0.3,0.4])	([0.5,0.5], [0.3,0.3])
$o_{5}$	([0.2,0.6], [0.3,0.3])	([0.6,0.8], [0.2,0.5])	([0.2,0.8], [0.5,0.6])	([0.6,0.6], [0.4,0.5])

**Table 11:** The normalized individual decision matrix given by DM $d_{3}$.

	$c_{1}$	$c_{2}$	$c_{3}$	$c_{4}$
$o_{1}$	([0.2,0.5], [0.6,0.7])	([0.3,0.6], [0.4,0.5])	([0.4,0.4], [0.5,0.5])	([0.4,0.7], [0.3,0.6])
$o_{2}$	([0.8,0.8], [0.1,0.1])	([0.6,0.8], [0.1,0.2])	([0.6,0.8], [0.4,0.6])	([0.5,0.9], [0.3,0.4])
$o_{3}$	([0.6,0.9], [0.4,0.5])	([0.7,0.7], [0.2,0.3])	([0.1,0.8], [0.4,0.6])	([0.1,0.8], [0.1,0.2])
$o_{4}$	([0.5,0.5], [0.3,0.4])	([0.5,0.7], [0.4,0.4])	([0.6,0.8], [0.1,0.4])	([0.3,0.8], [0.2,0.3])
$o_{5}$	([0.4,0.7], [0.6,0.6])	([0.5,0.8], [0.5,0.5])	([0.1,0.9], [0.2,0.3])	([0.5,0.6], [0.1,0.1])
Step 3.: Aggregating the normalized individual decision matrix using IVPQ-ROFWA operator. The aggregated results are shown in Table 12.

**Table 12:** The aggregated decision matrix.

	$c_{1}$	$c_{2}$	$c_{3}$	$c_{4}$
$o_{1}$	([0.4943,0.5784], [0.5378,0.6735])	([0.4027,0.5413], [0.2921,0.5233])	([0.4323,0.5413], [0.3976,0.4691])	([0.3843,0.6127], [0.3241,0.5045])
$o_{2}$	([0.7518,0.8285], [0.1000,0.1516])	([0.6249,0.8129], [0.1000,0.2305])	([0.5158,0.7518], [0.4573,0.6333])	([0.5158,0.8153], [0.1978,0.3041])
$o_{3}$	([0.7735,0.8442], [0.3260,0.4473])	([0.6755,0.7518], [0.1320,0.2280])	([0.3704,0.8037], [0.2219,0.3577])	([0.5004,0.7904], [0.1000,0.1682])
$o_{4}$	([0.4049,0.4585], [0.1845,0.2639])	([0.5891,0.6521], [0.3041,0.3722])	([0.4946,0.6499], [0.1469,0.3364])	([0.4027,0.6686], [0.1938,0.2711])
$o_{5}$	([0.2935,0.5944], [0.3959,0.4254])	([0.5011,0.7376], [0.2885,0.4401])	([0.2261,0.6499], [0.2318,0.3824])	([0.5011,0.6686], [0.1625,0.2089])
Step 4.: Aggregating the evaluated values under different attributes using GIVPQ-ROFHWGMSM operator (γ = 1.5). The aggregated results are shown as follows:
  $$Q_{1} = \left( {\left[ {0.0259,0.0816} \right],\left[ {0.6329,0.7612} \right]} \right)$$,
  $$Q_{2} = \left( {\left[ {0.0259,0.0816} \right],\left[ {0.6329,0.7612} \right]} \right)$$,
  $$Q_{3} = \left( {\left[ {0.1182,0.3492} \right],\left[ {0.3769,0.5359} \right]} \right)$$,
  $$Q_{4} = \left( {\left[ {0.0401,0.1130} \right],\left[ {0.3820,0.5262} \right]} \right)$$,
  $$Q_{5} = \left( {\left[ {0.0231,0.1902} \right],\left[ {0.4911,0.6134} \right]} \right)$$.
Step 5.: Calculating the projection of $Q_{i}$ on $Q^{ + }$ and $Q_{i}$ on $Q^{ - }$ based on Eqs. (34), (35). The results are shown as follows:
  $${\text{Proj}}_{{Q^{ + } }} \left( {Q_{1} } \right) = 0.7776$$,$${\text{Proj}}_{{Q^{ + } }} \left( {Q_{2} } \right) = 1.1766$$,$${\text{Proj}}_{{Q^{ + } }} \left( {Q_{3} } \right) = 1.1894$$,
  $${\text{Proj}}_{{Q^{ + } }} \left( {Q_{4} } \right) = 0.7776$$,$${\text{Proj}}_{{Q^{ + } }} \left( {Q_{5} } \right) = 1.0881$$;
  $${\text{Proj}}_{{Q^{ - } }} \left( {Q_{1} } \right) = 0.6220$$,$${\text{Proj}}_{{Q^{ - } }} \left( {Q_{2} } \right) = 0.9066$$,$${\text{Proj}}_{{Q^{ - } }} \left( {Q_{3} } \right) = 0.9147$$,
  $${\text{Proj}}_{{Q^{ - } }} \left( {Q_{3} } \right) = 0.9281$$,$${\text{Proj}}_{{Q^{ - } }} \left( {Q_{5} } \right) = 0.8398$$.
Step 6.: Computing the PID and NID for all *o*_*i*_ ∈ *O* based on Eqs. (36), (37). The results are presented as follows:
  $$P_{1}^{ + } = 0.5556$$,$$P_{2}^{ + } = 0.5648$$,$$P_{3}^{ + } = 0.5653$$,$$P_{4}^{ + } = 0.4559$$,$$P_{5}^{ + } = 0.5644$$,
  $$P_{1}^{ - } = 0.4444$$,$$P_{2}^{ - } = 0.4352$$,$$P_{3}^{ - } = 0.4347$$,$$P_{4}^{ - } = 0.5441$$,$$P_{5}^{ - } = 0.4356$$.
Step 7.: Calculating the regret -rejoice value for all *o*_*i*_∈*O* according to Eqs. (38)–(40). The results are shown as follows:
  $${\mathbb{R}}\left( {o_{1} } \right) = 0.0250$$,$${\mathbb{R}}\left( {o_{2} } \right) = 0.0302$$,$${\mathbb{R}}\left( {o_{3} } \right) = 0.0305$$,
  $${\mathbb{R}}\left( {o_{4} } \right) = - 0.0324$$,$${\mathbb{R}}\left( {o_{5} } \right) = 0.0300$$.
Step 8.: As a result, the ranking order of suppliers is $o_{3} \succ o_{2} \succ o_{5} \succ o_{1} \succ o_{4}$, which indicates that $o_{3}$ is the best alternative.

### Sensitivity analysis

In this part, in order to demonstrate the effectiveness and rationality of the proposed method, we conduct the sensitivity analysis to show the influence of the parameters $p$, $q$, $\alpha$, and $\beta$ on the decision results of all alternatives. For simplicity, the sensitivity analysis of parameters $\lambda_{1}$, $\lambda_{2}$ and $\gamma$ is omitted. Interested readers please refer to literature^35,49^. Since the Hamacher TN and TCN can be simplified into Algebraic TN and TCN when $\gamma = 1$, and Einstein TN and TCN when $\gamma = 2$, we only utilize the Hamacher TN and TCN to represent the family of ATN and ATCN.

**Figure 4:**
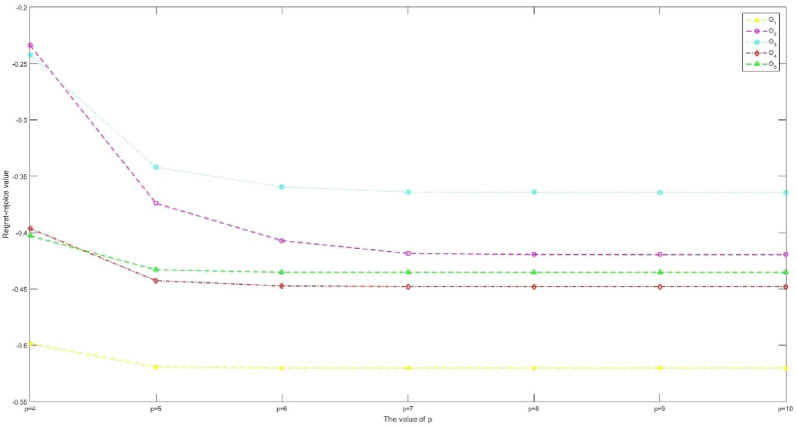
The sensitivity analysis of parameter $p$.

**Figure 5:**
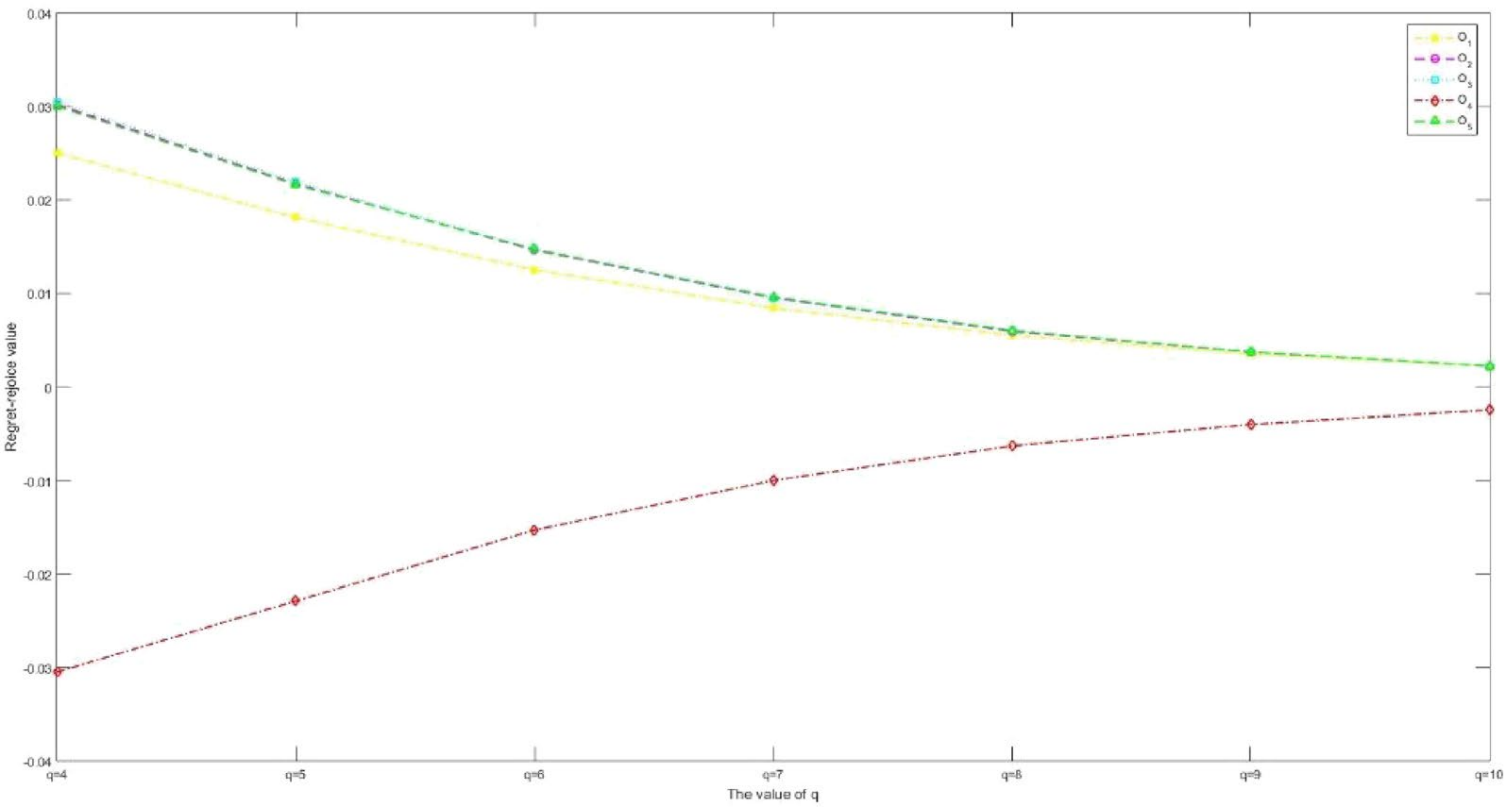
The sensitivity analysis of parameter $q$.

The decision results of the alternatives for influence of the parameters $p$ and $q$ (we set $\alpha = 0.88$, $\beta = 0.3$, $\gamma = 1.5$ and $\lambda_{1} = \lambda_{2} = 1$.) are presented in Tables 13, 14 and Figs. 4, 5. When analyzing parameter $p$, we fix parameter $q$, vice versa. From Tables 13, 14 and Figs. 4, 5, we can obtain some conclusions: (1) The ranking results are not influenced by different parameters $p$, but influenced by different parameter $q$. Parameter $p$ is taken from 4 to 10, and the ranking result remains $o_{3} \succ o_{2} \succ o_{5} \succ o_{1} \succ o_{4}$ without any change. However, for parameter $q$, the ranking results is $o_{3} \succ o_{2} \succ o_{5} \succ o_{1} \succ o_{4}$ when $q$ is equal to 4, and ranking results is $o_{3} = o_{5} \succ o_{2} \succ o_{1} \succ o_{4}$ when $q$ is equal to 6. (2) The regret rejoice value is impacted by both $p$ and $q$. According to Table 13, the regret-rejoice values of $o_{1}$, $o_{4}$ and $o_{5}$ are decreasing with the increase of $p$. While the regret-rejoice values of $o_{2}$ and $o_{3}$ are increasing with the increase of $p$. According to Table 14, the regret-rejoice values of $o_{4}$ are decreasing with the increase of $q$. While other regret-rejoice values are decreasing with the increase of $p$. (3) When parameters $p$ and $q$ are large enough, the score values of all alternative gradually become stable.

**Table 13:** The regret-rejoice values and ranking results with different parameter $p$.

$p$	${\mathbb{R}}\left( {o_{1} } \right)$	${\mathbb{R}}\left( {o_{2} } \right)$	${\mathbb{R}}\left( {o_{3} } \right)$	${\mathbb{R}}\left( {o_{4} } \right)$	${\mathbb{R}}\left( {o_{5} } \right)$	Ranking
$p = 4$	0.0250	0.0302	0.0305	− 0.0305	0.0300	$o_{3} \succ o_{2} \succ o_{5} \succ o_{1} \succ o_{4}$
$p = 5$	0. 0247	0.0304	0.0306	− 0.0326	0.0300	$o_{3} \succ o_{2} \succ o_{5} \succ o_{1} \succ o_{4}$
$p = 6$	0.0246	0.0305	0.0307	− 0.0327	0.0299	$o_{3} \succ o_{2} \succ o_{5} \succ o_{1} \succ o_{4}$
$p = 7$	0.0246	0.0305	0.0307	− 0.0328	0.0299	$o_{3} \succ o_{2} \succ o_{5} \succ o_{1} \succ o_{4}$
$p = 8$	0.0246	0.0305	0.0308	− 0.0328	0.0299	$o_{3} \succ o_{2} \succ o_{5} \succ o_{1} \succ o_{4}$
$p = 9$	0.0246	0.0305	0.0308	− 0.0328	0.0299	$o_{3} \succ o_{2} \succ o_{5} \succ o_{1} \succ o_{4}$
$p = 10$	0.0246	0.0306	0.0308	− 0.0328	0.0299	$o_{3} \succ o_{2} \succ o_{5} \succ o_{1} \succ o_{4}$

**Table 14:** The regret-rejoice values and ranking results with different parameter $q$.

$p$	${\mathbb{R}}\left( {o_{1} } \right)$	${\mathbb{R}}\left( {o_{2} } \right)$	${\mathbb{R}}\left( {o_{3} } \right)$	${\mathbb{R}}\left( {o_{4} } \right)$	${\mathbb{R}}\left( {o_{5} } \right)$	Ranking
$q = 4$	0.0250	0.0302	0.0305	− 0.0305	0.0300	$o_{3} \succ o_{2} \succ o_{5} \succ o_{1} \succ o_{4}$
$q = 5$	0.0181	0.0217	0.0219	− 0.0229	0.0216	$o_{3} \succ o_{2} \succ o_{5} \succ o_{1} \succ o_{4}$
$q = 6$	0.0125	0.0146	0.0147	− 0.0153	0.0147	$o_{3} = o_{5} \succ o_{2} \succ o_{1} \succ o_{4}$
$q = 7$	0.0084	0.0095	0.0095	− 0.0100	0.0096	$o_{5} \approx o_{3} = o_{2} \succ o_{1} \succ o_{4}$
$q = 8$	0.0055	0.0059	0.0060	− 0.0063	0.0060	$o_{3} = o_{5} \approx o_{2} \approx o_{1} \succ o_{4}$
$q = 9$	0.0035	0.0037	0.0037	− 0.0040	0.0037	$o_{3} = o_{2} = o_{5} \approx o_{1} \succ o_{4}$
$q = 10$	0.0022	0.0022	0.0022	− 0.0025	0.0022	$o_{3} = o_{2} = o_{5} = o_{1} \succ o_{4}$

**Figure 6:**
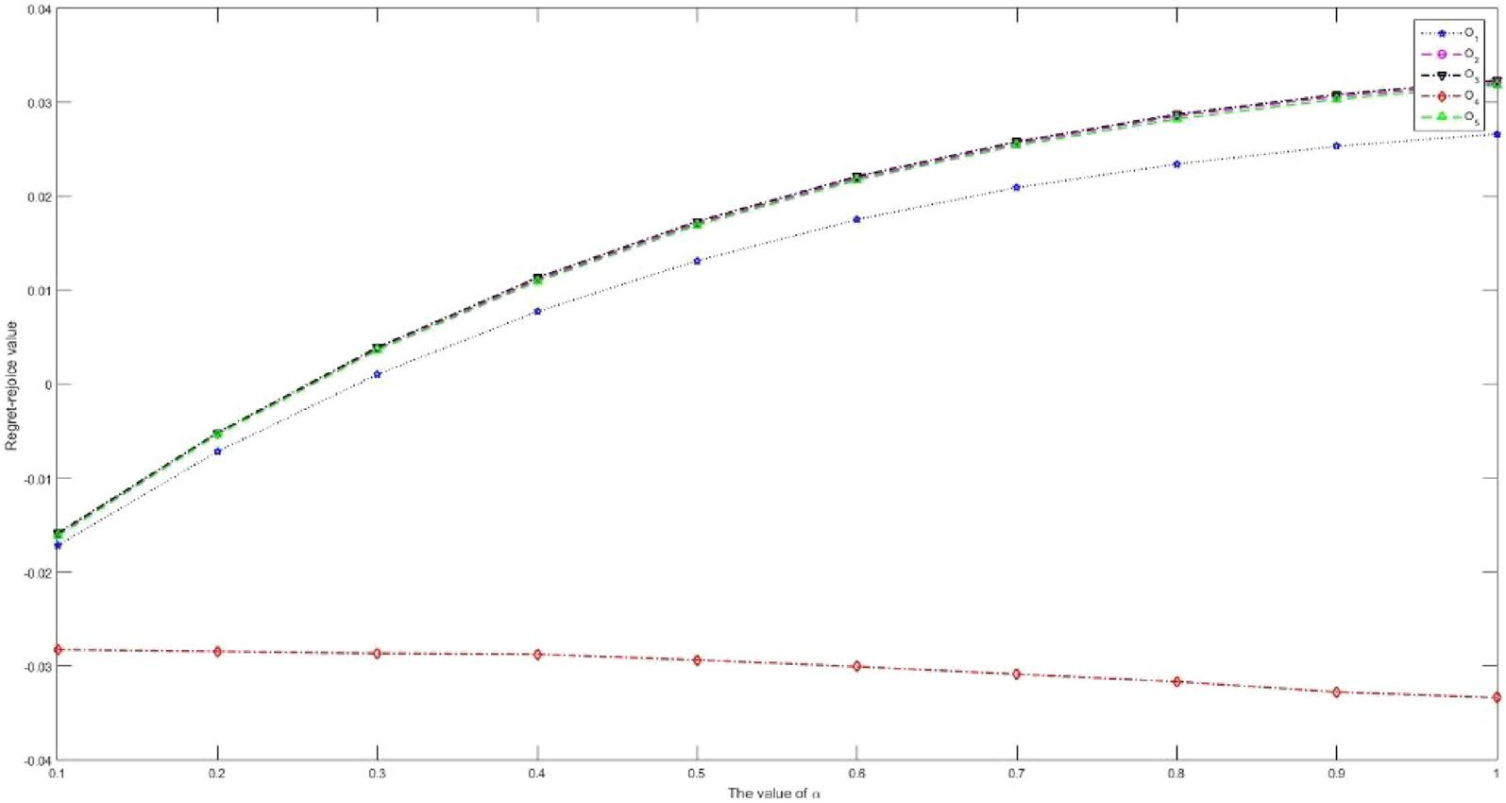
The sensitivity analysis of parameter $\alpha$.

The decision results of the alternatives for influence of the parameters $\alpha$ (we set $p = q = 4$, $\beta = 0.3$, $\gamma = 1.5$ and $\lambda_{1} = \lambda_{2} = 1$.) is shown in Fig. 6 and Table 15. From Fig. 6 and Table 15, we can obtain some conclusions: (1) The ranking results are not influenced by different parameters $\alpha$. The reason is that $1 - e^{{ - \beta \left( {u - u^{ + } } \right)}}$ are monotonically increases with respect to the utility value, and $1 - e^{{ - \beta \left( {x^{*} - x} \right)}}$ monotonically decreases with respect to the utility value. Therefore, the ranking order of five comprehensive utility values is constant. (2) The regret-rejoice value is influenced by parameter $\alpha$. the regret-rejoice values of $o_{4}$ are decreasing with the increase of $\alpha$. While other regret-rejoice values are decreasing with the increase of $\alpha$.

**Table 15:** The regret-rejoice values and ranking results with different parameter $\alpha$.

$\alpha$	${\mathbb{R}}\left( {o_{1} } \right)$	${\mathbb{R}}\left( {o_{2} } \right)$	${\mathbb{R}}\left( {o_{3} } \right)$	${\mathbb{R}}\left( {o_{4} } \right)$	${\mathbb{R}}\left( {o_{5} } \right)$	Ranking
$\alpha = 0.1$	− 0.0172	− 0.0160	− 0.0159	− 0.0283	− 0.0162	$o_{3} \succ o_{2} \succ o_{5} \succ o_{1} \succ o_{4}$
$\alpha = 0.2$	− 0.0072	− 0.0053	− 0.0052	− 0.0285	− 0.0054	$o_{3} \succ o_{2} \succ o_{5} \succ o_{1} \succ o_{4}$
$\alpha = 0.3$	0.0010	0.0037	0.0039	− 0.0287	0.0036	$o_{3} \succ o_{2} \succ o_{5} \succ o_{1} \succ o_{4}$
$\alpha = 0.4$	0.0077	0.0111	0.0113	− 0.0288	0.0109	$o_{3} \succ o_{2} \succ o_{5} \succ o_{1} \succ o_{4}$
$\alpha = 0.5$	0.0131	0.0171	0.0173	− 0.0294	0.0169	$o_{3} \succ o_{2} \succ o_{5} \succ o_{1} \succ o_{4}$
$\alpha = 0.6$	0.0175	0.0219	0.0221	− 0.0301	0.0217	$o_{3} \succ o_{2} \succ o_{5} \succ o_{1} \succ o_{4}$
$\alpha = 0.7$	0.0209	0.0256	0.0258	− 0.0309	0.0254	$o_{3} \succ o_{2} \succ o_{5} \succ o_{1} \succ o_{4}$
$\alpha = 0.8$	0.0234	0.0285	0.0287	− 0.0317	0.0282	$o_{3} \succ o_{2} \succ o_{5} \succ o_{1} \succ o_{4}$
$\alpha = 0.9$	0.0253	0.0306	0.0308	− 0.0328	0.0303	$o_{3} \succ o_{2} \succ o_{5} \succ o_{1} \succ o_{4}$
$\alpha = 1.0$	0.0266	0.0320	0.0323	− 0.0334	0.0318	$o_{3} \succ o_{2} \succ o_{5} \succ o_{1} \succ o_{4}$

**Figure 7:**
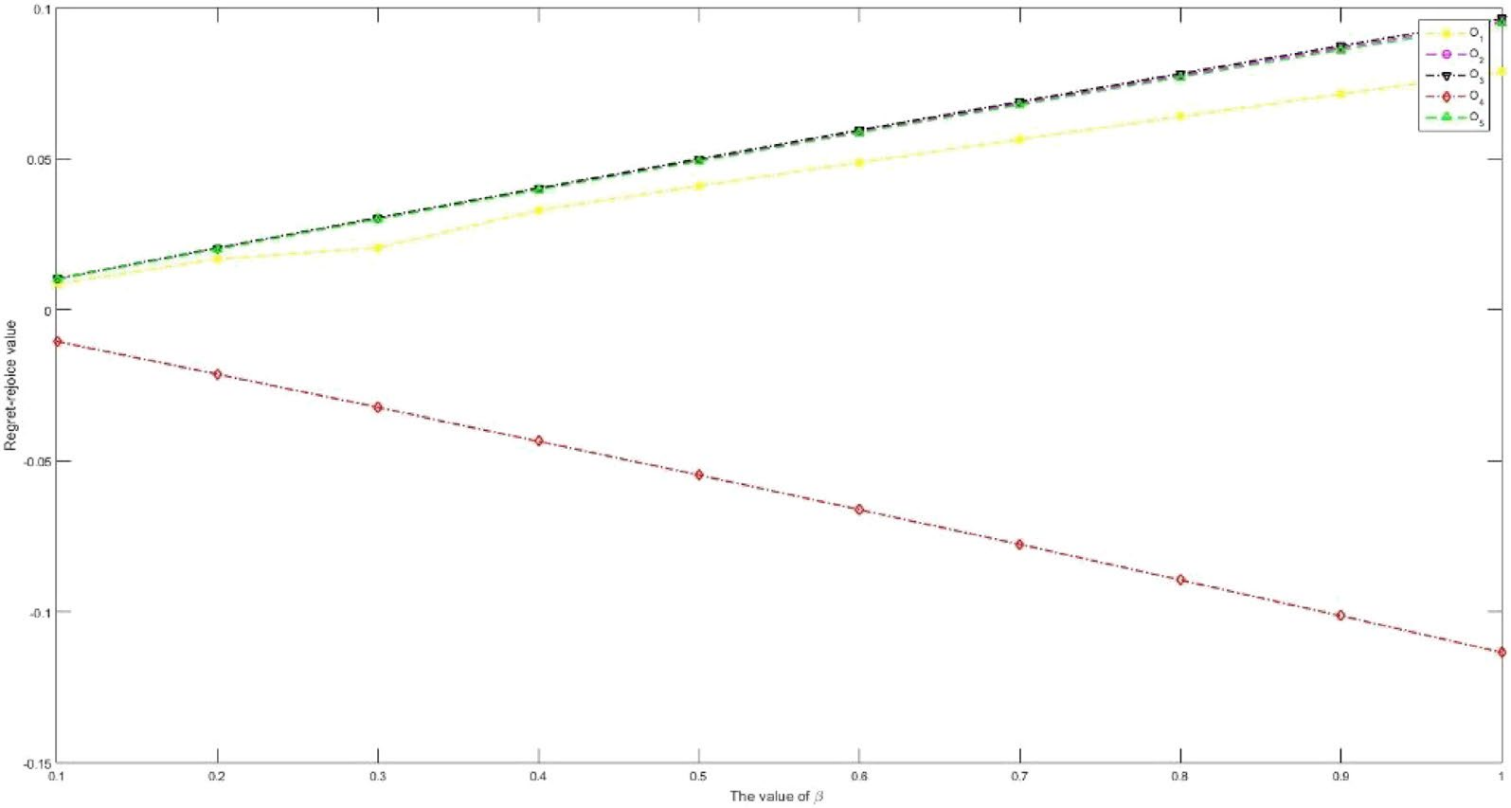
The sensitivity analysis of parameter $\beta$.

The decision results of the alternatives for influence of the parameters $\beta$ (we set $p = q = 4$, $\alpha = 0.88$, $\gamma = 1.5$ and $\lambda_{1} = \lambda_{2} = 1$.) is shown in Fig. 7 and Table 16. From Fig. 7 and Table 16, we can obtain same conclusions with the analysis of parameter $\alpha$. So, the sensitivity analysis of parameter $\beta$ is similar to the sensitivity analysis of parameter $\alpha$.

**Table 16:** The regret-rejoice values and ranking results with different parameter $\beta$.

$\beta$	${\mathbb{R}}\left( {o_{1} } \right)$	${\mathbb{R}}\left( {o_{2} } \right)$	${\mathbb{R}}\left( {o_{3} } \right)$	${\mathbb{R}}\left( {o_{4} } \right)$	${\mathbb{R}}\left( {o_{5} } \right)$	Ranking
$\beta = 0.1$	0.0085	0.0102	0.0103	− 0.0106	0.0101	$o_{3} \succ o_{2} \succ o_{5} \succ o_{1} \succ o_{4}$
$\beta = 0.2$	0.0168	0.0203	0.0205	− 0.0214	0.0201	$o_{3} \succ o_{2} \succ o_{5} \succ o_{1} \succ o_{4}$
$\beta = 0.3$	0.0205	0.0302	0.0305	− 0.0324	0.0300	$o_{3} \succ o_{2} \succ o_{5} \succ o_{1} \succ o_{4}$
$\beta = 0.4$	0.0330	0.0400	0.0403	− 0.0435	0.0397	$o_{3} \succ o_{2} \succ o_{5} \succ o_{1} \succ o_{4}$
$\beta = 0.5$	0.0410	0.0496	0.0500	− 0.0547	0.0492	$o_{3} \succ o_{2} \succ o_{5} \succ o_{1} \succ o_{4}$
$\beta = 0.6$	0.0488	0.0591	0.0596	− 0.0662	0.0587	$o_{3} \succ o_{2} \succ o_{5} \succ o_{1} \succ o_{4}$
$\beta = 0.7$	0.0565	0.0685	0.0690	− 0.0777	0.0679	$o_{3} \succ o_{2} \succ o_{5} \succ o_{1} \succ o_{4}$
$\beta = 0.8$	0.0640	0.0777	0.0783	− 0.0895	0.0771	$o_{3} \succ o_{2} \succ o_{5} \succ o_{1} \succ o_{4}$
$\beta = 0.9$	0.0715	0.0868	0.0875	− 0.1014	0.0861	$o_{3} \succ o_{2} \succ o_{5} \succ o_{1} \succ o_{4}$
$\beta = 1.0$	0.0788	0.0957	0.0965	− 0.1135	0.0950	$o_{3} \succ o_{2} \succ o_{5} \succ o_{1} \succ o_{4}$

## Comparative study

The superiority of the created MAGDM method cannot be fully conveyed by the numerical example presented in “Case study” section. In this part, we evaluate the effectiveness of the proposed MAGDM method to that of several related earlier MAGDM methods. Comparative analysis is mainly divided into two parts. The first part is a comparison with existing operators, such as the interval valued p,q Rung orthopair fuzzy weighted average (IVPQ-ROFWA) operator^20^, interval valued q-Rung orthopair fuzzy weighted Maclaurin symmetric mean (IVQ-ROFWMSM) operator^47^ and the interval valued q-Rung orthopair fuzzy weighted dual Maclaurin symmetric mean (IVQ-ROFWDMSM) operator^47^. The second part is a comparison with traditional decision-making methods, such as the Gao et al.’s VIKOR method^31^, Jeevaraj’s TOPSIS method^23^. The four aforementioned approaches are used to solve the SSS problem. Finally, we further compare the characteristics of our method and other existing popular methods.

### Comparison with existing operators

In this subsection, we will compare the proposed method with some existing operators.

#### Comparison with IVPQ-ROFWA operator

To tackle the SSS problem in “Case study” section, we apply the IVPQ-ROFWA operator^20^ (fixing $p = q = 4$). The main steps are presented as follows:

Step 1.: Aggregating the normalized individual decision matrix in Tables 8, 9, 10 based on Eq. (31). The aggregated results are the same as Table 12. So omitted here.
Step 2.: Aggregating the evaluation values under different attributes according to Eq. (31). The aggregated results are shown as follows:
  $$Q_{1} = \left( {\left[ {0.4336,0.5660} \right],\left[ {0.3753,0.5283} \right]} \right)$$,$$Q_{2} = \left( {\left[ {0.6225,0.7999,0.1893,0.3114} \right]} \right)$$,
  $$Q_{3} = \left( {\left[ {0.6313,0.7998} \right],\left[ {0.1792,0.2867} \right]} \right)$$,$$Q_{4} = \left( {\left[ {0.5017,0.6295} \right],\left[ {0.1968,0.3143} \right]} \right)$$,
  $$Q_{5} = \left( {\left[ {0.4225,0.7217} \right],\left[ {0.2557,0.3594} \right]} \right)$$.
Step 3.: Computing the score values of $Q_{i}$ based on Eq. (41).
  41 $$ S\left( {Q_{i} } \right) = \frac{1}{2}\left( {\left( {\mu_{i}^{U} } \right)^{p} + \left( {\mu_{i}^{L} } \right)^{p} - \left( {\nu_{i}^{U} } \right)^{q} - \left( {\nu_{i}^{L} } \right)^{q} } \right) $$
  The score values are shown as follows:
  $$S\left( {Q_{1} } \right) = 0.0201$$,$$S\left( {Q_{2} } \right) = 0.2745$$,$$S\left( {Q_{3} } \right) = 0.2801$$,$$S\left( {Q_{4} } \right) = 0.1045$$,$$S\left( {Q_{5} } \right) = 0.1411$$.
  The bigger the value of $S\left( {Q_{i} } \right)$, the better the supplier is. Thus, the ranking of the supplier is $o_{3} \succ o_{2} \succ o_{5} \succ o_{4} \succ o_{1}$ and the best supplier is $o_{3}$.

#### Comparison with IVQ-ROFWMSM operator

In this part, we apply the IVPQ-ROFWMSM operator^47^ (fixing $p = q = 4$) to solve the SSS problem. The main steps are presented as follows:

Step 1.: Aggregating the normalized individual decision matrix in Tables 8, 9, 10 based on Eq. (42). The aggregated results are the same as Table 12. So omitted here.
  42 $$ \begin{gathered} Q_{ij} = \left( {\left[ {\left( {1 - \prod\limits_{t = 1}^{T} {\left( {1 - \left( {\mu_{ij}^{Lt} } \right)^{p} } \right)^{{\delta_{t} }} } } \right)^{\frac{1}{p}} ,\left( {1 - \prod\limits_{t = 1}^{T} {\left( {1 - \left( {\mu_{ij}^{Ut} } \right)^{p} } \right)^{{\delta_{t} }} } } \right)^{\frac{1}{p}} } \right],\left[ {\prod\limits_{t = 1}^{T} {\left( {\nu_{ij}^{Lt} } \right)^{{\delta_{t} }} } ,\prod\limits_{t = 1}^{T} {\left( {\nu_{ij}^{Ut} } \right)^{{\delta_{t} }} } } \right]} \right) \\ = \left( {\left[ {\mu_{ij}^{L} ,\mu_{ij}^{U} } \right],\left[ {\nu_{ij}^{L} ,\nu_{ij}^{U} } \right]} \right) \\ \end{gathered} $$
Step 2.: Aggregating the evaluation values under different attributes based on Eq. (43).
  43 $$ Q_{i} = \left( \begin{gathered} \left[ {\left( {\left( {1 - \prod\limits_{{1 \le i_{1} < ... < i_{r} \le n}} {\left( {1 - \left( {\prod\limits_{j = 1}^{r} {\left( {\mu_{{i_{j} }}^{L} } \right)^{{w_{{i_{j} }} }} } } \right)^{q} } \right)^{{\frac{1}{{C_{n}^{r} }}}} } } \right)^{\frac{1}{q}} } \right)^{\frac{1}{r}} ,\left( {\left( {1 - \prod\limits_{{1 \le i_{1} < ... < i_{r} \le n}} {\left( {1 - \left( {\prod\limits_{j = 1}^{r} {\left( {\mu_{{i_{j} }}^{U} } \right)^{{w_{{i_{j} }} }} } } \right)^{q} } \right)^{{\frac{1}{{C_{n}^{r} }}}} } } \right)^{\frac{1}{q}} } \right)^{\frac{1}{r}} } \right], \hfill \\ \left[ {\left( {1 - \left( {1 - \left( {\prod\limits_{{1 \le i_{1} < ... < i_{r} \le n}}^{{}} {\left( {1 - \prod\limits_{j = 1}^{r} {\left( {1 - \left( {\nu_{{i_{j} }}^{L} } \right)^{q} } \right)^{{w_{{i_{j} }} }} } } \right)} } \right)^{{\frac{1}{{C_{n}^{r} }}}} } \right)^{\frac{1}{r}} } \right)^{\frac{1}{q}} ,\left( {1 - \left( {1 - \left( {\prod\limits_{{1 \le i_{1} < ... < i_{r} \le n}}^{{}} {\left( {1 - \prod\limits_{j = 1}^{r} {\left( {1 - \left( {\nu_{{i_{j} }}^{U} } \right)^{q} } \right)^{{w_{{i_{j} }} }} } } \right)} } \right)^{{\frac{1}{{C_{n}^{r} }}}} } \right)^{\frac{1}{r}} } \right)^{\frac{1}{q}} } \right] \hfill \\ \end{gathered} \right) $$
  The aggregated results are shown as follows:
  $$Q_{1} = \left( {\left[ {0.4336,0.5701} \right],\left[ {0.4006,0.5457} \right]} \right)$$, $$Q_{2} = \left( {\left[ {0.6024,0.8016} \right],\left[ {0.2376,0.3701} \right]} \right)$$,
  $$Q_{3} = \left( {\left[ {0.5820,0.7986} \right],\left[ {0.2140,0.3261} \right]} \right)$$,$$Q_{4} = \left( {\left[ {0.4811,0.6131} \right],\left[ {0.2141,0.3193} \right]} \right)$$,
  $$Q_{5} = \left( {\left[ {0.3840,0.7046} \right],\left[ {0.2854,0.3884} \right]} \right)$$.
Step 3.: Computing the score values of $Q_{i}$ based on Eq. (44).
  44 $$ S\left( {Q_{i} } \right) = \frac{1}{2}\left( {\left( {\mu_{i}^{U} } \right)^{q} + \left( {\mu_{i}^{L} } \right)^{q} - \left( {\nu_{i}^{U} } \right)^{q} - \left( {\nu_{i}^{L} } \right)^{q} } \right) $$
  The score values are shown as follows:
  $$S\left( {Q_{1} } \right) = 0.0132$$,$$S\left( {Q_{2} } \right) = 0.2613$$,$$S\left( {Q_{3} } \right) = 0.2541$$,$$S\left( {Q_{4} } \right) = 0.0912$$,$$S\left( {Q_{5} } \right) = 0.1194$$.
  The bigger the value of $S\left( {Q_{i} } \right)$, the better the supplier is. Thus, the ranking of the supplier is $o_{2} \succ o_{3} \succ o_{5} \succ o_{4} \succ o_{1}$ and the best supplier is $o_{2}$.

#### Comparison with IVQ-ROFWDMSM operator

In this subsection, IVPQ-ROFWDMSM operator^47^ is employed to tackle SSS problem (fixing $q = 4$). The main steps are presented as follows:

Step 1.: Aggregating the normalized individual decision matrix in Tables 8, 9, 10 based on Eq. (42). The aggregated results are the same as Table 12. So omitted here.
Step 2.: Aggregating the evaluation values under different attributes based on Eq. (45).
  45 $$ Q_{i} = \left( \begin{gathered} \left[ {\left( {1 - \left( {1 - \left( {\prod\limits_{{1 \le i_{1} < ... < i_{r} \le n}}^{{}} {\left( {1 - \prod\limits_{j = 1}^{r} {\left( {1 - \left( {\mu_{{i_{j} }}^{L} } \right)^{q} } \right)^{{w_{{i_{j} }} }} } } \right)} } \right)^{{\frac{1}{{C_{n}^{r} }}}} } \right)^{\frac{1}{r}} } \right)^{\frac{1}{q}} ,\left( {1 - \left( {1 - \left( {\prod\limits_{{1 \le i_{1} < ... < i_{r} \le n}}^{{}} {\left( {1 - \prod\limits_{j = 1}^{r} {\left( {1 - \left( {\mu_{{i_{j} }}^{U} } \right)^{q} } \right)^{{w_{{i_{j} }} }} } } \right)} } \right)^{{\frac{1}{{C_{n}^{r} }}}} } \right)^{\frac{1}{r}} } \right)^{\frac{1}{q}} } \right] \hfill \\ \left[ {\left( {\left( {1 - \prod\limits_{{1 \le i_{1} < ... < i_{r} \le n}} {\left( {1 - \left( {\prod\limits_{j = 1}^{r} {\left( {\nu_{{i_{j} }}^{L} } \right)^{{w_{{i_{j} }} }} } } \right)^{q} } \right)^{{\frac{1}{{C_{n}^{r} }}}} } } \right)^{\frac{1}{q}} } \right)^{\frac{1}{r}} ,\left( {\left( {1 - \prod\limits_{{1 \le i_{1} < ... < i_{r} \le n}} {\left( {1 - \left( {\prod\limits_{j = 1}^{r} {\left( {\nu_{{i_{j} }}^{U} } \right)^{{w_{{i_{j} }} }} } } \right)^{q} } \right)^{{\frac{1}{{C_{n}^{r} }}}} } } \right)^{\frac{1}{q}} } \right)^{\frac{1}{r}} } \right] \hfill \\ \end{gathered} \right) $$
  The aggregated results are shown as follows:
  $$Q_{1} = \left( {\left[ {0.4308,0.5657} \right],\left[ {0.3888,0.5429} \right]} \right)$$, $$Q_{2} = \left( {\left[ {0.6118,0.7997} \right],\left[ {0.2087,0.3315} \right]} \right)$$,
  $$Q_{3} = \left( {\left[ {0.6064,0.7997} \right],\left[ {0.2087,0.3315} \right]} \right)$$,$$Q_{4} = \left( {\left[ {0.4885,0.6234} \right],\left[ {0.2156,0.3175} \right]} \right)$$,
  $$Q_{5} = \left( {\left[ {0.4002,0.7094} \right],\left[ {0.2713,0.3676} \right]} \right)$$.
Step 3.: Computing the score values of $Q_{i}$ based on Eq. (44). The score values are shown as follows:
  $$S\left( {Q_{1} } \right) = 0.0135$$,$$S\left( {Q_{2} } \right) = 0.2675$$,$$S\left( {Q_{3} } \right) = 0.2655$$,$$S\left( {Q_{4} } \right) = 0.0978$$,$$S\left( {Q_{5} } \right) = 0.1276$$.
  The bigger the value of $S\left( {Q_{i} } \right)$, the better the supplier is. Thus, the ranking of the supplier is $o_{2} \succ o_{3} \succ o_{5} \succ o_{4} \succ o_{1}$ and the best supplier is $o_{2}$.

### Comparison with traditional MAGDM methods

In this section, we will compare the proposed method with some traditional MAGDM methods.

#### Comparison with IVQ-ROF VIKOR method

In this part, the IVQ-ROF VIKOR method^31^ is utilized to tackle the SSS problem (fixing $q = 4$). The specific procedure is shown as follows:

Step 1.: Similar to “Comparison with IVQ-ROFWMSM operator” section, we utilize the IVPQ-ROFWA operator to aggregate the normalized individual decision matrix in Tables 8, 9, 10. The aggregated results are the same as Table 12.
Step 2.: Determine the positive ideal solution (PID) and negative ideal solution (NID) according to the following Eqs. (46) and (47), respectively.
  46 $$ o^{ + } = \left( {Q_{1}^{ + } ,Q_{2}^{ + } ,...,Q_{j}^{ + } ,...,Q_{n}^{ + } } \right) $$
  47 $$ o^{ - } = \left( {Q_{1}^{ - } ,Q_{2}^{ - } ,...,Q_{j}^{ - } ,...,Q_{n}^{ - } } \right) $$
  where $$Q_{j}^{ + } = \left( {\left[ {\mathop {\max }\limits_{i} \left\{ {\mu_{ij}^{L} } \right\},\mathop {\max }\limits_{i} \left\{ {\mu_{ij}^{U} } \right\}} \right],\left[ {\mathop {\min }\limits_{i} \left\{ {\nu_{ij}^{L} } \right\},\mathop {\min }\limits_{i} \left\{ {\nu_{ij}^{U} } \right\}} \right]} \right)$$ and $$Q_{j}^{ + } = \left( {\left[ {\mathop {\min }\limits_{i} \left\{ {\mu_{ij}^{L} } \right\},\mathop {\min }\limits_{i} \left\{ {\mu_{ij}^{U} } \right\}} \right],\left[ {\mathop {\max }\limits_{i} \left\{ {\nu_{ij}^{L} } \right\},\mathop {\max }\limits_{i} \left\{ {\nu_{ij}^{U} } \right\}} \right]} \right)$$.
  The results are shown as follows:
  $$ o^{ + } = \left\{ \begin{gathered} \left( {\left[ {0.7735,0.8442} \right],\left[ {0.1000,0.1516} \right]} \right),\left( {\left[ {0.6755,0.8129} \right],\left[ {0.1000,0.2280} \right]} \right), \hfill \\ \left( {\left[ {0.5158,0.8037} \right],\left[ {0.1469,0.3364} \right]} \right),\left( {\left[ {0.5158,0.8153} \right],\left[ {0.1000,0.1682} \right]} \right) \hfill \\ \end{gathered} \right\} $$
  $$ o^{ - } = \left\{ \begin{gathered} \left( {\left[ {0.2935,0.4585} \right],\left[ {0.5378,0.6735} \right]} \right),\left( {\left[ {0.4027,0.5413} \right],\left[ {0.3041,0.6735} \right]} \right), \hfill \\ \left( {\left[ {0.2261,0.5413} \right],\left[ {0.4573,0.6333} \right]} \right),\left( {\left[ {0.3843,0.6127} \right],\left[ {0.3241,0.5045} \right]} \right) \hfill \\ \end{gathered} \right\} $$
Step 3.: Determine the mean $\mathop I\limits^{\sim }_{i}$ and the worst $\mathop I\limits^{ - }_{i}$ based on Eqs. (48), (49).
  48 $$ \mathop I\limits^{\sim }_{i} = \sum\limits_{j}^{n} {w_{j} \frac{{d\left( {Q_{j}^{ + } ,Q_{ij} } \right)}}{{d\left( {Q_{j}^{ + } ,Q_{j}^{ - } } \right)}}} $$
  49 $$ \mathop I\limits^{\sim }_{i} = \mathop {\max }\limits_{j} \left\{ {w_{j} \frac{{d\left( {Q_{j}^{ + } ,Q_{ij} } \right)}}{{d\left( {Q_{j}^{ + } ,Q_{j}^{ - } } \right)}}} \right\} $$
  where $d$ is the distance measure and $d\left( {Q_{1} ,Q_{2} } \right) = \frac{1}{4}\left( {\left| {\left( {\mu_{1}^{L} } \right)^{q} - \left( {\mu_{2}^{L} } \right)^{q} } \right| + \left| {\left( {\mu_{1}^{U} } \right)^{q} - \left( {\mu_{2}^{U} } \right)^{q} } \right| + \left| {\left( {\nu_{1}^{L} } \right)^{q} - \left( {\nu_{2}^{L} } \right)^{q} } \right| + \left| {\left( {\nu_{1}^{U} } \right)^{q} - \left( {\nu_{2}^{U} } \right)^{q} } \right|} \right)$.
  The results are shown as follows:
  $$\mathop I\limits^{\sim }_{1} = 0.9336$$, $$\mathop I\limits^{\sim }_{2} = 1.0807$$, $$\mathop I\limits^{\sim }_{3} = 1.0024$$, $$\mathop I\limits^{\sim }_{4} = 0.9429$$, $$\mathop I\limits^{\sim }_{5} = 0.9210$$,
  $$\mathop I\limits^{\sim }_{1} = 0.1591$$, $$\mathop I\limits^{\sim }_{2} = 0.1018$$, $$\mathop I\limits^{\sim }_{3} = 0.0725$$, $$\mathop I\limits^{\sim }_{4} = 0.1534$$, $$\mathop I\limits^{\sim }_{5} = 0.1638$$.
Step 4.: Compute the $\Omega_{i}$ according to Eq. (50).
  50 $$ \Omega_{i} = \theta \left( {\frac{{\tilde{I}_{i} - \tilde{I}^{ - } }}{{\tilde{I}^{ + } - \tilde{I}^{ - } }}} \right) + \left( {1 - \theta } \right)\left( {\frac{{\overline{I} - \overline{I}^{ - } }}{{\overline{I}^{ + } - \overline{I}^{ - } }}} \right) $$
  where $$\tilde{I}^{ + } = \mathop {\max }\limits_{i} \left\{ {\tilde{I}_{i} } \right\}$$, $$\tilde{I}^{ - } = \mathop {\min }\limits_{i} \left\{ {\tilde{I}_{i} } \right\}$$, $$\overline{I}^{ + } = \mathop {\max }\limits_{i} \left\{ {\overline{I}_{i} } \right\}$$ and $$\overline{I}^{ - } = \mathop {\min }\limits_{i} \left\{ {\overline{I}_{i} } \right\}$$.
  The results are obtained as follows:
  $$\Omega_{1} = 0.5137$$, $$\Omega_{2} = 0.6607$$, $$\Omega_{3} = 0.2550$$, $$\Omega_{4} = 0.5118$$, $$\Omega_{5} = 0.5000$$.
  The smaller the value of $\Omega_{i}$, the better the supplier is. Then the ranking results of suppliers is $o_{3} \succ o_{5} \succ o_{4} \succ o_{1} \succ o_{2}$ and the best supplier is $o_{3}$.

#### Comparison with IVFF-TOPSIS method

In this section, the IVFF-TOPSIS method^23^ is applied to solve the SSS problem (fixing $q = 3$). The specific procedure is presented as follows:

Step 1.: Similar to IVQ-ROF VIKOR method, the IVQ-ROFWA operator is used to aggregated the normalized individual decision matrixes. The aggregated results are shown in Table 17.

**Table 17:** The aggregated decision matrix.

	$c_{1}$	$c_{2}$	$c_{3}$	$c_{4}$
$o_{1}$	([0.4813,0.5633], [0.5378,0.6735])	([0.3885,0.5314], [0.2921,0.5233])	([0.4249,0.5314], [0.3976,0.4691])	([0.3795,0.6055], [0.3241,0.5045])
$o_{2}$	([0.7457,0.8231], [0.1000,0.1516])	([0.6176,0.8060], [0.1000,0.2305])	([0.5085,0.7457], [0.4573,0.6333])	([0.5085,0.8097], [0.1978,0.3041])
$o_{3}$	([0.7602,0.8381], [0.3260,0.4473])	([0.6696,0.7457], [0.1320,0.2280])	([0.3484,0.7498], [0.2219,0.3577])	([0.4821,0.7827], [0.1000,0.1682])
$o_{4}$	([0.3945,0.4516], [0.1845,0.2639])	([0.5774,0.6453], [0.3041,0.3722])	([0.4887,0.6363], [0.1469,0.3364])	([0.3885,0.6557], [0.1938,0.2711])
$o_{5}$	([0.2765,0.5851], [0.3959,0.4254])	([0.4843,0.7295], [0.2885,0.4401])	([0.2144,0.7703], [0.2318,0.3824])	([0.4843,0.6524], [0.1625,0.2089])
Step 2.: Compute the weighted decision matrix according to Eq. (52). The weighted decision matrix is shown in Table 18.
  51 $$ {\mathbb{N}} = \left[ {w_{j} Q_{ij} } \right]_{m \times n} $$

**Table 18:** The weighted decision matrix.

	$c_{1}$	$c_{2}$	$c_{3}$	$c_{4}$
$o_{1}$	([0.2905,0.3435], [0.8779,0.9204])	([0.2498,0.3459], [0.7261,0.8450])	([0.2962,0.3738], [0.7376,0.7789])	([0.2236,0.3658], [0.7983,0.8721])
$o_{2}$	([0.4738,0.5400], [0.6166,0.6729])	([0.4701,0.5597], [0.5495,0.6828])	([0.3569,0.5451], [0.7724,0.8600])	([0.3029,0.5198], [0.7232,0.7882])
$o_{3}$	([0.4855,0.5542], [0.7903,0.8446])	([0.4459,0.5065], [0.5906,0.6808])	([0.2419,0.5901], [0.6085,0.7123])	([0.2864,0.4695], [0.6310,0.7001])
$o_{4}$	([0.2365,0.2719], [0.7013,0.7560])	([0.3781,0.4275], [0.7338,0.7734])	([0.3424,0.4541], [0.5310,0.6980])	([0.2290,0.4002], [0.7202,0.7702])
$o_{5}$	([0.1648,0.3580], [0.8232,0.8357])	([0.3137,0.4931], [0.7239,0.8078])	([0.1483,0.5673], [0.6173,0.7281])	([0.2877,0.3979], [0.6953,0.7311])
Step 3.: Determine the PIS and NIS based on Eqs. (52), (53).
  52 $$ o^{ + } = \left( {Q_{1}^{ + } ,Q_{2}^{ + } ,...,Q_{j}^{ + } ,...,Q_{n}^{ + } } \right) $$
  53 $$ o^{ - } = \left( {Q_{1}^{ - } ,Q_{2}^{ - } ,...,Q_{j}^{ - } ,...,Q_{n}^{ - } } \right) $$
  where $$Q_{j}^{ + } = \left( {\left[ {\mathop {\max }\limits_{i} \left\{ {\mu_{ij}^{L} } \right\},\mathop {\max }\limits_{i} \left\{ {\mu_{ij}^{U} } \right\}} \right],\left[ {\mathop {\min }\limits_{i} \left\{ {\nu_{ij}^{L} } \right\},\mathop {\min }\limits_{i} \left\{ {\nu_{ij}^{U} } \right\}} \right]} \right)$$ and $$Q_{j}^{ + } = \left( {\left[ {\mathop {\min }\limits_{i} \left\{ {\mu_{ij}^{L} } \right\},\mathop {\min }\limits_{i} \left\{ {\mu_{ij}^{U} } \right\}} \right],\left[ {\mathop {\max }\limits_{i} \left\{ {\nu_{ij}^{L} } \right\},\mathop {\max }\limits_{i} \left\{ {\nu_{ij}^{U} } \right\}} \right]} \right)$$.
  The results are shown as follows:
  $$ o^{ + } = \left\{ \begin{gathered} \left( {\left[ {0.4855,0.5542} \right],\left[ {0.6166,0.6729} \right]} \right),\left( {\left[ {0.4459,0.5597} \right],\left[ {0.5499,0.6808} \right]} \right), \hfill \\ \left( {\left[ {0.3569,0.5901} \right],\left[ {0.5310,0.6980} \right]} \right),\left( {\left[ {0.3029,0.5198} \right],\left[ {0.6310,0.7001} \right]} \right) \hfill \\ \end{gathered} \right\} $$
  $$ o^{ - } = \left\{ \begin{gathered} \left( {\left[ {0.1648,0.2719} \right],\left[ {0.8779,0.9204} \right]} \right),\left( {\left[ {0.2498,0.3459} \right],\left[ {0.7338,0.9204} \right]} \right), \hfill \\ \left( {\left[ {0.1483,0.3738} \right],\left[ {0.4573,0.6333} \right]} \right),\left( {\left[ {0.2236,0.3658} \right],\left[ {0.7983,0.8721} \right]} \right) \hfill \\ \end{gathered} \right\} $$
Step 4.: Calculate the distance between $o_{i}$ and $o^{ + }$, and the distance between $o_{i}$ and $o^{ - }$ based on Eqs. (54), (55).
  54 $$ d\left( {o_{i} ,o^{ + } } \right) = \sum\limits_{j = 1}^{n} {d\left( {Q_{ij} ,Q_{j}^{ + } } \right)} $$
  55 $$ d\left( {o_{i} ,o^{ - } } \right) = \sum\limits_{j = 1}^{n} {d\left( {Q_{ij} ,Q_{j}^{ - } } \right)} $$
  where $d$ is the distance measure and $d\left( {Q_{1} ,Q_{2} } \right) = \frac{1}{4}\left( {\left| {\left( {\mu_{1}^{L} } \right)^{q} - \left( {\mu_{2}^{L} } \right)^{q} } \right| + \left| {\left( {\mu_{1}^{U} } \right)^{q} - \left( {\mu_{2}^{U} } \right)^{q} } \right| + \left| {\left( {\nu_{1}^{L} } \right)^{q} - \left( {\nu_{2}^{L} } \right)^{q} } \right| + \left| {\left( {\nu_{1}^{U} } \right)^{q} - \left( {\nu_{2}^{U} } \right)^{q} } \right|} \right)$.
  The obtained results are presented as follows:
  $$d\left( {o_{1} ,o^{ + } } \right) = 1.0735$$, $$d\left( {o_{2} ,o^{ + } } \right) = 0.5971$$, $$d\left( {o_{3} ,o^{ + } } \right) = 0.6039$$,
  $$d\left( {o_{4} ,o^{ + } } \right) = 0.6614$$, $$d\left( {o_{5} ,o^{ + } } \right) = 0.7573$$,
  $$d\left( {o_{1} ,o^{ - } } \right) = 1.3663$$, $$d\left( {o_{2} ,o^{ - } } \right) = 1.0947$$, $$d\left( {o_{3} ,o^{ - } } \right) = 1.1972$$,
  $$d\left( {o_{4} ,o^{ - } } \right) = 1.1432$$, $$d\left( {o_{5} ,o^{ - } } \right) = 1.2567$$.
Step 5.: Compute the closeness degree of each supplier according to Eq. (56).
  56 $$ CD\left( {o_{i} } \right) = \frac{{d\left( {o_{i} ,o^{ + } } \right)}}{{d\left( {o_{i} ,o^{ + } } \right) + d\left( {o_{i} ,o^{ - } } \right)}} $$
  The obtained results are shown as follows:
  $$CD\left( {o_{1} } \right) = 0.5600$$, $$CD\left( {o_{2} } \right) = 0.6471$$, $$CD\left( {o_{3} } \right) = 0.6647$$, $$CD\left( {o_{4} } \right) = 0.6335$$, $$CD\left( {o_{5} } \right) = 0.6240$$.
  The higher the closeness degree, the better the supplier is. Therefore, the ranking result of all supplier is $o_{3} \succ o_{2} \succ o_{4} \succ o_{5} \succ o_{1}$ and the best supplier is $o_{3}$.

### Results and discussion

Based on the data in Table 19, we offer the following analysis and commentary:

1. The method proposed in this article has a smaller difference in ranking results compared to the IVPQ-ROFWA operator^28^. The optimal supplier is all $o_{3}$. The main difference lies in the ranking of $o_{4}$ and $o_{1}$. According to the ranking results of the IVPQ-ROFWA operator, $o_{4}$ is better than $o_{1}$; According to the results obtained from our method, $o_{1}$ is superior to $o_{4}$. However, the IVPQ-ROFWA operator cannot handle the correlation between attributes, and it also ignores the risk preference and psychological state of decision-makers.
2. The method proposed in this article has a significant difference in ranking results compared to the IVQ-ROFWMSM operator^47^ and IVQ-ROFWDMSM^47^ operator. According to the results of the IVQ-ROFWMSM operator and IVQ-ROFWDMSM operator, the optimal supplier is $o_{2}$, and according to the results of our method, the optimal solution is $o_{3}$. Meanwhile, according to the ranking results of these two methods, $o_{4}$ is superior to scheme $o_{1}$; based on the results obtained from our method, $o_{1}$ is superior to $o_{4}$. However, the operator proposed in this article is based on the GMSM operator, and IVQ-ROFSs are also a special form of IVPQ-ROFSs. The IVQ-ROFWMSM operator and IVQ-ROFWDMSM operator also ignore the risk preferences and psychological states of decision-makers.
3. Compared with the IVFF TOPSIS method^23^, there are also some differences in the ranking results, mainly in the ranking of $o_{1}$, $o_{4}$, and $o_{5}$. Based on the ranking results of IVFF TOPSIS, the ranking order is $o_{4} \succ o_{5} \succ o_{1}$. According to the results of our method, the ranking order is $o_{5} \succ o_{1} \succ o_{4}$. However, the TOPSIS method uses the WA operator in information aggregation, while this method uses the GMSM operator in information aggregation. GMSM can effectively handle the correlation between attributes. Meanwhile, IVFFS is a special form of IVPQ-ROFS. The TOPSIS method also cannot reflect the psychological state and risk preferences of DMs.
4. Compared with the IVQ-ROF VIKOR^31^ method, there are large differences in the ranking results, mainly in the ranking of $o_{1}$, $o_{2}$, $o_{4}$ and $o_{5}$. Based on the ranking results of IVQ-ROF VIKOR, the ranking order is $o_{5} \succ o_{4} \succ o_{1} \succ o_{2}$. According to the results of our method, the ranking order is $o_{2} \succ o_{4} \succ o_{5} \succ o_{1}$. The optimal supplier obtained from both methods is $o_{3}$. Similar to IVFF TOPSIS method, our method has advantages in information aggregation, information representation, and handling decision-makers' risk preferences and psychological states.

**Table 19:** Ranking results of the proposed method and other decision-making methods.

Method	$o_{1}$	$o_{2}$	$o_{3}$	$o_{4}$	$o_{5}$	Ranking
IVPQ-WA operator ($S\left( {Q_{i} } \right)$)	0.0201	0.2705	0.2801	0.1045	0.1411	$o_{3} \succ o_{2} \succ o_{5} \succ o_{4} \succ o_{1}$
IVQ-ROFWMSM operator ($S\left( {Q_{i} } \right)$)	0.0132	0.2613	0.2541	0.0912	0.1194	$o_{2} \succ o_{3} \succ o_{5} \succ o_{4} \succ o_{1}$
IVQ-ROFWDMSM operator ($S\left( {Q_{i} } \right)$)	0.0135	0.2675	0.2655	0.0978	0.1276	$o_{2} \succ o_{3} \succ o_{5} \succ o_{4} \succ o_{1}$
IVQ-ROF VIKOR ($\Omega_{i}$)	0.5137	0.6607	0.2550	0.5118	0.5000	$o_{3} \succ o_{5} \succ o_{4} \succ o_{1} \succ o_{2}$
IVQ-FF TOPSIS ($CD\left( {o_{i} } \right)$)	0.5600	0.6471	0.6647	0.6335	0.6240	$o_{3} \succ o_{2} \succ o_{4} \succ o_{5} \succ o_{1}$
The proposed method (${\mathbb{R}}\left( {o_{i} } \right)$)	0.0250	0.0302	0.0305	− 0.0305	0.0300	$o_{3} \succ o_{2} \succ o_{5} \succ o_{1} \succ o_{4}$

Based on the analysis in Table 20, the main merit of our method can be organized as follows:

1. For information representation, compared with the methods in literatures^23,31,47,66^, we utilize the IVPQ-ROFS to represent the evaluation information, which is a generalized form of IVPFS, IVFFS and IFQ-ROFS.
2. For the operational laws of FS, compared with the Algebraic laws in literatures^23,28,31,47,66^, the operational laws of IVPQ-ROFS based on ATN and ATCN are more generalized.
3. For information aggregation, compared with the weighted average (WA) operator in literatures^23,28,31,66^, the GMSM operator could effectively handle the interrelation between attributes. Compared with the weighted Maclaurin symmetric mean (WMSM) operator in literatures^47^, the GMSM operator is a more generalized form.
4. For describing the psychological state and the risk preference of DMs, we modify the regret function and rejoice function, which consider both PIP and NIP.
5. For the interrelation between attributes, we introduce the IVPQ-ROFGMSM operator and IVPQ-ROFWMSM operator. Some special cases and valuable properties are provided.
6. For the similarity measure, compared with the distance measure in^23,28,31,47,66^, we introduce the projection of IVPQ-ROFNs, which consider both distance and angle between IVPQ-ROFNs.

**Table 20:** A comparison of the characteristics and differences for different methods.

Methods	Information representation	Operational laws	Information aggregation	Psychological state and risk attitude	Interrelation between attributes	Similarity measure
IVPQ-ROFWA operator^28^	IVPQ-ROFS	Algebraic TN and TCN	WA operator	No	No	Distance
IVQ-ROFWMSM operator^47^	IVQ-ROFS	Algebraic TN and TCN	WMSM operator	No	Yes	None
IVQ-ROFWDMSM operator^47^	IVQ-ROFS	Algebraic TN and TCN	WDMSM operator	No	Yes	None
IVFF TOPSIS^23^	IVFFS	Algebraic TN and TCN	WA operator	No	No	Distance
IVQ-ROF VIKOR^31^	IVQ-ROFS	Algebraic TN and TCN	WA operator	No	No	Distance
IVPQ-ROF TODIM^28^	IVPQ-ROFS	Algebraic TN and TCN	WA operator	Yes	No	Distance
IVPF TODIM^66^	IVPFS	Algebraic TN and TCN	WA operator	Yes	No	Distance
The proposed method	IVPQ-ROFS	ATN and ATCN	GMSM operator	Yes	Yes	Projection

However, there are also some shortcomings in our method

1. Compared with the IVPQ-ROF TODIM^28^ method, we have not designed a method to solve the weight of indicators.
2. In the MAGDM problem, the level of consensus among DMs and the process of reaching consensus are also important^67,68^, and we did not study these issues in this article
3. The method proposed in this article can only rank the alternatives and select the optimal one. When the number of alternatives is large, it is often necessary to classify them^69,70^, and the clustering and classification problems in MAGDM method are also not studied in this article.

### Ethical approval

This study did not involve human or animal subjects, and thus, no ethical approval was required. The study protocol adhered to the guidelines established by the journal.

### Consent to participate

All participants are consent to participate in this research.

## Conclusions and future work

Considering that IVPQ-ROFSs are extended forms of IFSs, IVIFSs, PFSs, IVPFSs, Q-ROFSs and IVQ-ROFSs, and have certain research value. This article proposes a new IVPQ-ROF MAGDM method for SSS problem. The main conclusions of this paper are organized as follows:

1. Based on the research results in literature^28^, we have improved the operation rules of IVPQ-ROFSs. This paper first proposes the Archimedean operation rules of IVPQ-ROFSs, and proves the effectiveness and rationality of this operation rule through some examples. The Archimedean operation rules of IVPQ-ROFSs proposed in this paper are a more generalized form, and the Algebraic operation rules of IVPQ-ROFSs in literature^28^ are its special form.
2. Inspired by literature^65^, this paper proposes the modulus and cosine of IVPQ-ROFSs, and based on this, proposes the projection measure of IVPQ-ROFSs, thus PID and NID can be defined. Compared to the distance measure in literature^28^, the projection measure proposed in this paper comprehensively considers the distance and angle between IVPQ-ROFNs. At the same time, PID and NID based on projection measurement comprehensively consider the PIP and NIP, reflecting information more comprehensively.
3. Considering that in the aggregation stage of attribute information, the weighted average operator in literature^28^ assumes that each attribute is independent of each other and cannot handle the problem of correlation between attributes, this study extends the GMSM operator to IVPQ-ROF environments. Compared with the weighted average operator, it is more effective in handling the correlation relationships between attributes.
4. Based on the research results in literature^57–60^, we propose new regret and rejoice functions. When calculating regret and rejoice values, the new regret and rejoice functions consider both the optimal and worst solutions. Compared with traditional regret and rejoice functions, the new regret and rejoice functions proposed in this paper are more effective in measuring DMs' risk preferences and psychological states.
5. This article successfully applies the proposed method to the SSS problem, and proves its superiority and rationality through case analysis, sensitivity analysis, and comparative analysis.

Future efforts will aim to address the demerits explained in “Results and discussion” section. In addition, the practical problem addressed in this article is the issue of SSS, and there are many MAGDM issues worth studying in reality, such as: evaluation of sustainable strategies for urban parcel delivery^71^, efficient city supply chain management^72^, factory location selection^73^, evaluation of green-house effect^74^ and economic condition analysis^75^.

## Supplementary Information

Supplementary Information.

## Data Availability

The data used to support the findings of this study are available from the corresponding author upon request.
