# Supplementary material for: A generalized interval-valued p,q Rung orthopair fuzzy Maclaurin symmetric mean and modified regret theory based sustainable supplier selection method
Source: Sci Rep. 2024 Jun 28;14:14942. doi: 10.1038/s41598-024-64765-3 (PMC11761347; doi:10.1038/s41598-024-64765-3)
Supplement: Supplementary file 1 — Supplementary Information. [file 41598_2024_64765_MOESM1_ESM.docx]

**Appendix A (The proof of Theorem 1)**

According to the operational laws for IVPQ-QOFNs in Definition 5, we have

$$Q_{i_{j}}^{\lambda_{j}}=\left(\left[g^{-1}\left(\lambda_{j}g\left(\mu_{i_{j}}^{L}\right)\right),g^{-1}\left(\lambda_{j}g\left(\mu_{i_{j}}^{U}\right)\right)\right],\left[h^{-1}\left(\lambda_{j}h\left(\nu_{i_{j}}^{L}\right)\right),h^{-1}\left(\lambda_{j}h\left(\nu_{i_{j}}^{U}\right)\right)\right]\right)$$

$$\otimes_{j=1}^{k} Q_{i_{j}}^{\lambda_{j}}=\left(\left[g^{-1}\left(\sum_{j=1}^{k} \lambda_{j}g\left(\mu_{i_{j}}^{L}\right)\right),g^{-1}\left(\sum_{j=1}^{k} \lambda_{j}g\left(\mu_{i_{j}}^{U}\right)\right)\right],\left[h^{-1}\left(\sum_{j=1}^{k} \lambda_{j}h\left(\nu_{i_{j}}^{L}\right)\right),h^{-1}\left(\sum_{j=1}^{k} \lambda_{j}h\left(\nu_{i_{j}}^{U}\right)\right)\right]\right)$$

$$\oplus_{1\leq i_{1}<...<i_{k}\leq n} \left(\otimes_{j=1}^{k} Q_{i_{j}}^{\lambda_{j}}\right)=\left(\begin{matrix} \left[h^{-1}\left(\sum_{1\leq i_{1}<...<i_{k}\leq n} h\left(g^{-1}\left(\sum_{j=1}^{k} \lambda_{j}g\left(\mu_{i_{j}}^{L}\right)\right)\right)\right),h^{-1}\left(\sum_{1\leq i_{1}<...<i_{k}\leq n} h\left(g^{-1}\left(\sum_{j=1}^{k} \lambda_{j}g\left(\mu_{i_{j}}^{U}\right)\right)\right)\right)\right] \\ \left[g^{-1}\left(\sum_{1\leq i_{1}<...<i_{k}\leq n} g\left(h^{-1}\left(\sum_{j=1}^{k} \lambda_{j}h\left(\nu_{i_{j}}^{L}\right)\right)\right)\right),g^{-1}\left(\sum_{1\leq i_{1}<...<i_{k}\leq n} g\left(h^{-1}\left(\sum_{j=1}^{k} \lambda_{j}h\left(\nu_{i_{j}}^{U}\right)\right)\right)\right)\right] \end{matrix}\right)$$

$$\frac{1}{C_{n}^{k}}\oplus_{1\leq i_{1}<...<i_{k}\leq n} \left(\otimes_{j=1}^{k} Q_{i_{j}}^{\lambda_{j}}\right)=\left(\begin{matrix} \left[h^{-1}\left(\frac{1}{C_{n}^{k}}\sum_{1\leq i_{1}<...<i_{k}\leq n} h\left(g^{-1}\left(\sum_{j=1}^{k} \lambda_{j}g\left(\mu_{i_{j}}^{L}\right)\right)\right)\right),h^{-1}\left(\frac{1}{C_{n}^{k}}\sum_{1\leq i_{1}<...<i_{k}\leq n} h\left(g^{-1}\left(\sum_{j=1}^{k} \lambda_{j}g\left(\mu_{i_{j}}^{U}\right)\right)\right)\right)\right] \\ \left[g^{-1}\left(\frac{1}{C_{n}^{k}}\sum_{1\leq i_{1}<...<i_{k}\leq n} g\left(h^{-1}\left(\sum_{j=1}^{k} \lambda_{j}h\left(\nu_{i_{j}}^{L}\right)\right)\right)\right),g^{-1}\left(\frac{1}{C_{n}^{k}}\sum_{1\leq i_{1}<...<i_{k}\leq n} g\left(h^{-1}\left(\sum_{j=1}^{k} \lambda_{j}h\left(\nu_{i_{j}}^{U}\right)\right)\right)\right)\right] \end{matrix}\right)$$

$$\left(\frac{1}{C_{n}^{k}}\oplus_{1\leq i_{1}<...<i_{k}\leq n} \left(\otimes_{j=1}^{k} Q_{i_{j}}^{\lambda_{j}}\right)\right)^{\frac{1}{\lambda_{1}+\lambda_{2}+...+\lambda_{n}}}=\left(\begin{matrix} \left[\begin{matrix} g^{-1}\left(\frac{1}{\lambda_{1}+\lambda_{2}+...+\lambda_{k}}g\left(h^{-1}\left(\frac{1}{C_{n}^{k}}\sum_{1\leq i_{1}<...<i_{k}\leq n} h\left(g^{-1}\left(\sum_{j=1}^{k} \lambda_{j}g\left(\mu_{i_{j}}^{L}\right)\right)\right)\right)\right)\right), \\ g^{-1}\left(\frac{1}{\lambda_{1}+\lambda_{2}+...+\lambda_{k}}g\left(h^{-1}\left(\frac{1}{C_{n}^{k}}\sum_{1\leq i_{1}<...<i_{k}\leq n} h\left(g^{-1}\left(\sum_{j=1}^{k} \lambda_{j}g\left(\mu_{i_{j}}^{U}\right)\right)\right)\right)\right)\right) \end{matrix}\right], \\ \left[\begin{matrix} h^{-1}\left(\frac{1}{\lambda_{1}+\lambda_{2}+...+\lambda_{k}}h\left(g^{-1}\left(\frac{1}{C_{n}^{k}}\sum_{1\leq i_{1}<...<i_{k}\leq n} g\left(h^{-1}\left(\sum_{j=1}^{k} \lambda_{j}h\left(\nu_{i_{j}}^{L}\right)\right)\right)\right)\right)\right), \\ h^{-1}\left(\frac{1}{\lambda_{1}+\lambda_{2}+...+\lambda_{k}}h\left(g^{-1}\left(\frac{1}{C_{n}^{k}}\sum_{1\leq i_{1}<...<i_{k}\leq n} g\left(h^{-1}\left(\sum_{j=1}^{k} \lambda_{j}h\left(\nu_{i_{j}}^{U}\right)\right)\right)\right)\right)\right) \end{matrix}\right] \end{matrix}\right)$$

Then, the proof is completed.

**Appendix B (The proof of Property 1)**

1. For the membership part, we have

$$\begin{matrix} a^{⋄}=g^{-1}\left(\frac{1}{\lambda_{1}+\lambda_{2}+...+\lambda_{k}}g\left(h^{-1}\left(\frac{1}{C_{n}^{k}}\sum_{1\leq i_{1}<...<i_{k}\leq n} h\left(g^{-1}\left(\sum_{j=1}^{k} \lambda_{j}g\left(\mu_{i_{j}}^{⋄}\right)\right)\right)\right)\right)\right) \\ =g^{-1}\left(\frac{1}{\lambda_{1}+\lambda_{2}+...+\lambda_{k}}g\left(h^{-1}\left(\frac{1}{C_{n}^{k}}C_{n}^{k}h\left(g^{-1}\left(\sum_{j=1}^{k} \lambda_{j}g\left(\mu^{⋄}\right)\right)\right)\right)\right)\right) \\ =g^{-1}\left(\frac{1}{\lambda_{1}+\lambda_{2}+...+\lambda_{k}}g\left(h^{-1}\left(h\left(g^{-1}\left(\sum_{j=1}^{k} \lambda_{j}g\left(\mu^{⋄}\right)\right)\right)\right)\right)\right) \\ =g^{-1}\left(\frac{1}{\lambda_{1}+\lambda_{2}+...+\lambda_{k}}\sum_{j=1}^{k} \lambda_{j}g\left(\mu^{⋄}\right)\right)=\mu^{⋄} \end{matrix}$$

Similarly, for the non-membership part, we can get $b^{⋄}=\nu^{⋄}$. So (1) is proved.

1. For convenience, $Q=\left(\left[\mu^{L},\mu^{U}\right],\left[\nu^{L},\nu^{U}\right]\right)$ and $Q^{*}=\left(\left[\mu^{L*},\mu^{U*}\right],\left[\nu^{L*},\nu^{U*}\right]\right)$ are used to denote the aggregated results of the two operators, namely

$$GIVPQ-QOFMSM^{\left(k,\lambda_{1},\lambda_{2},...,\lambda_{k}\right)}\left(Q_{1},Q_{2},...,Q_{n}\right)=\left(\left[\mu^{L},\mu^{U}\right],\left[\nu^{L},\nu^{U}\right]\right)$$

$$GIVPQ-QOFMSM^{\left(k,\lambda_{1},\lambda_{2},...,\lambda_{k}\right)}\left(Q_{1}^{*},Q_{2}^{*},...,Q_{n}^{*}\right)=\left(\left[\mu^{L*},\mu^{U*}\right],\left[\nu^{L*},\nu^{U*}\right]\right)$$

For membership part, since $\mu_{i}^{U}\leq\mu_{i}^{U*}$, $\mu_{i}^{L}\leq\mu_{i}^{L*}$, we can get $\sum_{j=1}^{k} \lambda_{j}g\left(\mu_{i_{j}}^{^{⋄}}\right)\geq\sum_{j=1}^{k} \lambda_{j}g\left(\mu_{i_{j}}^{^{⋄*}}\right)$ and $g^{-1}\left(\sum_{j=1}^{k} \lambda_{j}g\left(\mu_{i_{j}}^{⋄}\right)\right)\leq g^{-1}\left(\sum_{j=1}^{k} \lambda_{j}g\left(\mu_{i_{j}}^{⋄*}\right)\right)$.

Then we have

$$h\left(g^{-1}\left(\sum_{j=1}^{k} \lambda_{j}g\left(\mu_{i_{j}}^{^{⋄}}\right)\right)\right)\leq h\left(g^{-1}\left(\sum_{j=1}^{k} \lambda_{j}g\left(\mu_{i_{j}}^{^{⋄*}}\right)\right)\right)$$

$$\frac{1}{C_{n}^{k}}\sum_{1\leq i_{1}<...<i_{k}\leq n} h\left(g^{-1}\left(\sum_{j=1}^{k} \lambda_{j}g\left(\mu_{i_{j}}^{⋄}\right)\right)\right)\leq\frac{1}{C_{n}^{k}}\sum_{1\leq i_{1}<...<i_{k}\leq n} h\left(g^{-1}\left(\sum_{j=1}^{k} \lambda_{j}g\left(\mu_{i_{j}}^{⋄*}\right)\right)\right)$$

Further we can obtain

$$h^{-1}\left(\frac{1}{C_{n}^{k}}\sum_{1\leq i_{1}<...<i_{k}\leq n} h\left(g^{-1}\left(\sum_{j=1}^{k} \lambda_{j}g\left(\mu_{i_{j}}^{⋄}\right)\right)\right)\right)\leq h^{-1}\left(\frac{1}{C_{n}^{k}}\sum_{1\leq i_{1}<...<i_{k}\leq n} h\left(g^{-1}\left(\sum_{j=1}^{k} \lambda_{j}g\left(\mu_{i_{j}}^{⋄*}\right)\right)\right)\right)$$

$$\begin{matrix} \frac{1}{\lambda_{1}+\lambda_{2}+...+\lambda_{k}}g\left(h^{-1}\left(\frac{1}{C_{n}^{k}}\sum_{1\leq i_{1}<...<i_{k}\leq n} h\left(g^{-1}\left(\sum_{j=1}^{k} \lambda_{j}g\left(\mu_{i_{j}}^{⋄}\right)\right)\right)\right)\right) \\ \geq\frac{1}{\lambda_{1}+\lambda_{2}+...+\lambda_{k}}g\left(h^{-1}\left(\frac{1}{C_{n}^{k}}\sum_{1\leq i_{1}<...<i_{k}\leq n} h\left(g^{-1}\left(\sum_{j=1}^{k} \lambda_{j}g\left(\mu_{i_{j}}^{⋄}\right)\right)\right)\right)\right) \end{matrix}$$

Finally, we have

$$\begin{matrix} g^{-1}\left(\frac{1}{\lambda_{1}+\lambda_{2}+...+\lambda_{k}}g\left(h^{-1}\left(\frac{1}{C_{n}^{k}}\sum_{1\leq i_{1}<...<i_{k}\leq n} h\left(g^{-1}\left(\sum_{j=1}^{k} \lambda_{j}g\left(\mu_{i_{j}}^{⋄}\right)\right)\right)\right)\right)\right) \\ \leq g^{-1}\left(\frac{1}{\lambda_{1}+\lambda_{2}+...+\lambda_{k}}g\left(h^{-1}\left(\frac{1}{C_{n}^{k}}\sum_{1\leq i_{1}<...<i_{k}\leq n} h\left(g^{-1}\left(\sum_{j=1}^{k} \lambda_{j}g\left(\mu_{i_{j}}^{⋄*}\right)\right)\right)\right)\right)\right) \end{matrix}$$

Similarly, for non-membership part, as $\nu_{i}^{U}\geq\nu_{i}^{U*}$ and $\nu_{i}^{L}\geq\nu_{i}^{L*}$, we can obtain

$$\begin{matrix} h^{-1}\left(\frac{1}{\lambda_{1}+\lambda_{2}+...+\lambda_{k}}h\left(g^{-1}\left(\frac{1}{C_{n}^{k}}\sum_{1\leq i_{1}<...<i_{k}\leq n} g\left(h^{-1}\left(\sum_{j=1}^{k} \lambda_{j}h\left(\nu_{i_{j}}^{⋄}\right)\right)\right)\right)\right)\right) \\ \geq h^{-1}\left(\frac{1}{\lambda_{1}+\lambda_{2}+...+\lambda_{k}}h\left(g^{-1}\left(\frac{1}{C_{n}^{k}}\sum_{1\leq i_{1}<...<i_{k}\leq n} g\left(h^{-1}\left(\sum_{j=1}^{k} \lambda_{j}h\left(\nu_{i_{j}}^{⋄*}\right)\right)\right)\right)\right)\right) \end{matrix}$$

Based on the score function and comparison rules of IVPQ-QOFNs in Definition 6 and Definition 7 respectively, we have $S\left(Q\right)\leq S\left(Q^{*}\right)$.

1. If $S\left(Q\right)<S\left(Q^{*}\right)$, then $Q<Q^{*}$
2. If $S\left(Q\right)=S\left(Q^{*}\right)$, then $\left[\mu^{L},\mu^{U}\right]=\left[\mu^{L*},\mu^{U*}\right]$ and $\left[\nu^{L},\nu^{U}\right]=\left[\nu^{L*},\nu^{U*}\right]$, hence $A\left(Q\right)=A\left(Q^{*}\right)$, which testifies $Q=Q^{*}$.

Thus, we can get $Q\leq Q^{*}$.

1. It can be easily deduced based on (1) and (2).

**Appendix C (The proof of Theorem 2)**

1. When $1\leq k<n$, according to the operational laws for IVPQ-QOFNs in Definition 5, we have

$$Q_{i_{j}}^{\lambda_{j}}=\left(\left[g^{-1}\left(\lambda_{j}g\left(\mu_{i_{j}}^{L}\right)\right),g^{-1}\left(\lambda_{j}g\left(\mu_{i_{j}}^{U}\right)\right)\right],\left[h^{-1}\left(\lambda_{j}h\left(\nu_{i_{j}}^{L}\right)\right),h^{-1}\left(\lambda_{j}h\left(\nu_{i_{j}}^{U}\right)\right)\right]\right)$$

$$\otimes_{j=1}^{k} Q_{i_{j}}^{\lambda_{j}}=\left(\left[g^{-1}\left(\sum_{j=1}^{k} \lambda_{j}g\left(\mu_{i_{j}}^{L}\right)\right),g^{-1}\left(\sum_{j=1}^{k} \lambda_{j}g\left(\mu_{i_{j}}^{U}\right)\right)\right],\left[h^{-1}\left(\sum_{j=1}^{k} \lambda_{j}h\left(\nu_{i_{j}}^{L}\right)\right)\text{，}h^{-1}\left(\sum_{j=1}^{k} \lambda_{j}h\left(\nu_{i_{j}}^{U}\right)\right)\right]\right)$$

$$\left(1-w_{i_{j}}\right)\otimes_{j=1}^{k} Q_{i_{j}}^{\lambda_{j}}=\left(\begin{matrix} \left[h^{-1}\left(\left(1-w_{i_{j}}\right)h\left(g^{-1}\left(\sum_{j=1}^{k} \lambda_{j}g\left(\mu_{i_{j}}^{L}\right)\right)\right)\right),h^{-1}\left(\left(1-w_{i_{j}}\right)h\left(g^{-1}\left(\sum_{j=1}^{k} \lambda_{j}g\left(\mu_{i_{j}}^{U}\right)\right)\right)\right)\right], \\ \left[g^{-1}\left(\left(1-w_{i_{j}}\right)g\left(h^{-1}\left(\sum_{j=1}^{k} \lambda_{j}h\left(\nu_{i_{j}}^{L}\right)\right)\right)\right),g^{-1}\left(\left(1-w_{i_{j}}\right)g\left(h^{-1}\left(\sum_{j=1}^{k} \lambda_{j}h\left(\nu_{i_{j}}^{U}\right)\right)\right)\right)\right] \end{matrix}\right)$$

$$\oplus_{1\leq i_{1}<...<i_{k}\leq n} \left(1-w_{i_{j}}\right)\left(\otimes_{j=1}^{k} Q_{i_{j}}^{\lambda_{j}}\right)=\left(\begin{matrix} \left[\begin{matrix} h^{-1}\left(\sum_{1\leq i_{1}<...<i_{k}\leq n} \left(1-w_{i_{j}}\right)h\left(g^{-1}\left(\sum_{j=1}^{k} \lambda_{j}g\left(\mu_{i_{j}}^{L}\right)\right)\right)\right), \\ h^{-1}\left(\sum_{1\leq i_{1}<...<i_{k}\leq n} \left(1-w_{i_{j}}\right)h\left(g^{-1}\left(\sum_{j=1}^{k} \lambda_{j}g\left(\mu_{i_{j}}^{U}\right)\right)\right)\right) \end{matrix}\right], \\ \left[\begin{matrix} g^{-1}\left(\sum_{1\leq i_{1}<...<i_{k}\leq n} \left(1-w_{i_{j}}\right)g\left(h^{-1}\left(\sum_{j=1}^{k} \lambda_{j}h\left(\nu_{i_{j}}^{L}\right)\right)\right)\right), \\ g^{-1}\left(\sum_{1\leq i_{1}<...<i_{k}\leq n} \left(1-w_{i_{j}}\right)g\left(h^{-1}\left(\sum_{j=1}^{k} \lambda_{j}h\left(\nu_{i_{j}}^{U}\right)\right)\right)\right) \end{matrix}\right] \end{matrix}\right)$$

$$\frac{1}{C_{n-1}^{k}}\oplus_{1\leq i_{1}<...<i_{k}\leq n} \left(1-w_{i_{j}}\right)\left(\otimes_{j=1}^{k} Q_{i_{j}}^{\lambda_{j}}\right)=\left(\begin{matrix} \left[\begin{matrix} h^{-1}\left(\frac{1}{C_{n-1}^{k}}\sum_{1\leq i_{1}<...<i_{k}\leq n} \left(1-w_{i_{j}}\right)h\left(g^{-1}\left(\sum_{j=1}^{k} \lambda_{j}g\left(\mu_{i_{j}}^{L}\right)\right)\right)\right)\text{,} \\ h^{-1}\left(\frac{1}{C_{n-1}^{k}}\sum_{1\leq i_{1}<...<i_{k}\leq n} \left(1-w_{i_{j}}\right)h\left(g^{-1}\left(\sum_{j=1}^{k} \lambda_{j}g\left(\mu_{i_{j}}^{U}\right)\right)\right)\right) \end{matrix}\right] \\ \left[\begin{matrix} g^{-1}\left(\frac{1}{C_{n-1}^{k}}\sum_{1\leq i_{1}<...<i_{k}\leq n} \left(1-w_{i_{j}}\right)g\left(h^{-1}\left(\sum_{j=1}^{k} \lambda_{j}h\left(\nu_{i_{j}}^{L}\right)\right)\right)\right), \\ g^{-1}\left(\frac{1}{C_{n-1}^{k}}\sum_{1\leq i_{1}<...<i_{k}\leq n} \left(1-w_{i_{j}}\right)g\left(h^{-1}\left(\sum_{j=1}^{k} \lambda_{j}h\left(\nu_{i_{j}}^{U}\right)\right)\right)\right) \end{matrix}\right] \end{matrix}\right)$$

$$\begin{matrix} \left(\frac{1}{C_{n-1}^{k}}\oplus_{1\leq i_{1}<...<i_{k}\leq n} \left(1-w_{i_{j}}\right)\left(\otimes_{j=1}^{k} Q_{i_{j}}^{\lambda_{j}}\right)\right)^{\frac{1}{\lambda_{1}+\lambda_{2}+...+\lambda_{k}}} \\ =\left(\begin{matrix} \left[\begin{matrix} g^{-1}\left(\frac{1}{\lambda_{1}+\lambda_{2}+...+\lambda_{k}}g\left(h^{-1}\left(\frac{1}{C_{n-1}^{k}}\sum_{1\leq i_{1}<...<i_{k}\leq n} \left(1-w_{i_{j}}\right)h\left(g^{-1}\left(\sum_{j=1}^{k} \lambda_{j}g\left(\mu_{i_{j}}^{L}\right)\right)\right)\right)\right)\right), \\ g^{-1}\left(\frac{1}{\lambda_{1}+\lambda_{2}+...+\lambda_{k}}g\left(h^{-1}\left(\frac{1}{C_{n-1}^{k}}\sum_{1\leq i_{1}<...<i_{k}\leq n} \left(1-w_{i_{j}}\right)h\left(g^{-1}\left(\sum_{j=1}^{k} \lambda_{j}g\left(\mu_{i_{j}}^{U}\right)\right)\right)\right)\right)\right) \end{matrix}\right], \\ \left[\begin{matrix} h^{-1}\left(\frac{1}{\lambda_{1}+\lambda_{2}+...+\lambda_{k}}h\left(g^{-1}\left(\frac{1}{C_{n-1}^{k}}\sum_{1\leq i_{1}<...<i_{k}\leq n} \left(1-w_{i_{j}}\right)g\left(h^{-1}\left(\sum_{j=1}^{k} \lambda_{j}h\left(\nu_{i_{j}}^{L}\right)\right)\right)\right)\right)\right), \\ h^{-1}\left(\frac{1}{\lambda_{1}+\lambda_{2}+...+\lambda_{k}}h\left(g^{-1}\left(\frac{1}{C_{n-1}^{k}}\sum_{1\leq i_{1}<...<i_{k}\leq n} \left(1-w_{i_{j}}\right)g\left(h^{-1}\left(\sum_{j=1}^{k} \lambda_{j}h\left(\nu_{i_{j}}^{U}\right)\right)\right)\right)\right)\right) \end{matrix}\right] \end{matrix}\right) \end{matrix}$$

1. When $k=n$, according to the operational laws for IVPQ-QOFNs in Definition 5, we have

$$Q_{i}^{\lambda_{i}+\frac{1-nw_{i}}{n-1}}=\left(\begin{matrix} \left[g^{-1}\left(\left(\lambda_{i}+\frac{1-nw_{i}}{n-1}\right)g\left(\mu_{i}^{L}\right)\right),g^{-1}\left(\left(\lambda_{i}+\frac{1-nw_{i}}{n-1}\right)g\left(\mu_{i}^{U}\right)\right)\right], \\ \left[h^{-1}\left(\left(\lambda_{i}+\frac{1-nw_{i}}{n-1}\right)h\left(\nu_{i}^{L}\right)\right),h^{-1}\left(\left(\lambda_{i}+\frac{1-nw_{i}}{n-1}\right)h\left(\nu_{i}^{U}\right)\right)\right] \end{matrix}\right)$$

$$\otimes_{i=1}^{k} Q_{i}^{\lambda_{i}+\frac{1-nw_{i}}{n-1}}=\left(\begin{matrix} \left[g^{-1}\left(\sum_{i=1}^{k} \left(\lambda_{i}+\frac{1-nw_{i}}{n-1}\right)g\left(\mu_{i}^{L}\right)\right),g^{-1}\left(\sum_{i=1}^{k} \left(\lambda_{i}+\frac{1-nw_{i}}{n-1}\right)g\left(\mu_{i}^{U}\right)\right)\right], \\ \left[h^{-1}\left(\sum_{i=1}^{k} \left(\lambda_{i}+\frac{1-nw_{i}}{n-1}\right)h\left(\nu_{i}^{L}\right)\right),h^{-1}\left(\sum_{i=1}^{k} \left(\lambda_{i}+\frac{1-nw_{i}}{n-1}\right)h\left(\nu_{i}^{U}\right)\right)\right] \end{matrix}\right)$$

$$\left(\otimes_{i=1}^{k} Q_{i}^{\lambda_{i}+\frac{1-nw_{i}}{n-1}}\right)^{\frac{1}{\lambda_{1}+\lambda_{2}+...+\lambda_{k}}}=\left(\begin{matrix} \left[\begin{matrix} g^{-1}\left(\frac{1}{\lambda_{1}+\lambda_{2}+...+\lambda_{k}}\sum_{i=1}^{k} \left(\lambda_{i}+\frac{1-nw_{i}}{n-1}\right)g\left(\mu_{i}^{L}\right)\right), \\ g^{-1}\left(\frac{1}{\lambda_{1}+\lambda_{2}+...+\lambda_{k}}\sum_{i=1}^{k} \left(\lambda_{i}+\frac{1-nw_{i}}{n-1}\right)g\left(\mu_{i}^{U}\right)\right) \end{matrix}\right] \\ \left[\begin{matrix} h^{-1}\left(\frac{1}{\lambda_{1}+\lambda_{2}+...+\lambda_{k}}\sum_{i=1}^{k} \left(\lambda_{i}+\frac{1-nw_{i}}{n-1}\right)h\left(\nu_{i}^{L}\right)\right), \\ h^{-1}\left(\frac{1}{\lambda_{1}+\lambda_{2}+...+\lambda_{k}}\sum_{i=1}^{k} \left(\lambda_{i}+\frac{1-nw_{i}}{n-1}\right)h\left(\nu_{i}^{U}\right)\right) \end{matrix}\right] \end{matrix}\right)$$

Then, the proof is completed.

**Appendix D. The symbols used in our method**

| **Symbol** | **Means** |
| --- | --- |
| $\mathop{Q}\limits^{\sim}$ | the IVPQ-ROFS |
| $Q$ | the IVPQ-ROFN |
| $\mu^{U}$ | the upper bound of MD |
| $\mu^{L}$ | the lower bound of MD |
| $\nu^{U}$ | the upper bound of NMD |
| $\nu^{L}$ | the lower bound of NMD |
| $p,q$ | the parameters in IVPQ-ROFS |
| $k$ | the least common multiple of $p$ and $q$. |
| $g$ | the monotonic decreasing function |
| $h$ | the monotonic increasing function |
| $T\left(x,y\right)$ | the T-norm |
| $S\left(x,y\right)$ | the T-conorm |
| $k,\lambda_{1},\lambda_{2},...,\lambda_{k}$ | the parameters in GMSM operator |
| $u$ | the utility function |
| $\alpha$ | the risk aversion coefficient |
| $s$ | the evaluation value in utility function |
| $R\left(\Delta u\right)$ | the regret-rejoice function |
| $\beta$ | the regret aversion coefficient |
| $REG$ | the regret value |
| $REJ$ | the rejoice value |
|  | the regret-rejoice value |
| $\gamma$ | the parameter in Hamacher TN and TCN |
| $\tau$ | the parameter in Frank TN and TCN |
| $\left\vert Q\right\vert$ | the module of $Q$ |
| $\cos$ | The cosine between two IVPQ-ROFNs |
| $Q^{+}$ | the PID |
| $Q^{-}$ | the NID |
| $\Pr\text{oj}$ | the projection measure of IVPQ-ROFNs |
| $P^{+}$ | the PID |
| $P^{-}$ | the NID |
| $O=\left\{o_{1},o_{2},...,o_{m}\right\}$ | the set of alternatives |
| $C=\left\{c_{1},c_{2},...,c_{n}\right\}$ | the set of attributes |
| $D=\left\{d_{1},d_{2},...,d_{T}\right\}$ | the set of DMs |
| $W=\left\{w_{1},w_{2},...,w_{n}\right\}$ | the vector of attributes |
| $\delta=\left(\delta_{1},\delta_{2},...,\delta_{T}\right)$ | the vector of DMs |
|  | the decision matrix |
